# A Review on the Vagus Nerve and Autonomic Nervous System During Fetal Development: Searching for Critical Windows

**DOI:** 10.3389/fnins.2021.721605

**Published:** 2021-09-20

**Authors:** Francesco Cerritelli, Martin G. Frasch, Marta C. Antonelli, Chiara Viglione, Stefano Vecchi, Marco Chiera, Andrea Manzotti

**Affiliations:** ^1^Research and Assistance for Infants to Support Experience Lab, Foundation Center for Osteopathic Medicine Collaboration, Pescara, Italy; ^2^Department of Obstetrics and Gynecology and Center on Human Development and Disability, University of Washington, Seattle, WA, United States; ^3^Facultad de Medicina, Instituto de Biología Celular y Neurociencia “Prof. E. De Robertis”, Universidad de Buenos Aires, Buenos Aires, Argentina; ^4^Department of Obstetrics and Gynecology, Klinikum Rechts der Isar, Technical University of Munich, Munich, Germany; ^5^Department of Pediatrics, Division of Neonatology, “V. Buzzi” Children's Hospital, Azienda Socio-Sanitaria Territoriale Fatebenefratelli Sacco, Milan, Italy; ^6^Research Department, Istituto Osteopatia Milano, Milan, Italy

**Keywords:** fetal development, autonomic nervous system, vagus nerve, cholinergic anti-inflammatory pathway, heart rate variability, critical window, maternal health

## Abstract

The autonomic nervous system (ANS) is one of the main biological systems that regulates the body's physiology. Autonomic nervous system regulatory capacity begins before birth as the sympathetic and parasympathetic activity contributes significantly to the fetus' development. In particular, several studies have shown how vagus nerve is involved in many vital processes during fetal, perinatal, and postnatal life: from the regulation of inflammation through the anti-inflammatory cholinergic pathway, which may affect the functioning of each organ, to the production of hormones involved in bioenergetic metabolism. In addition, the vagus nerve has been recognized as the primary afferent pathway capable of transmitting information to the brain from every organ of the body. Therefore, this hypothesis paper aims to review the development of ANS during fetal and perinatal life, focusing particularly on the vagus nerve, to identify possible “critical windows” that could impact its maturation. These “critical windows” could help clinicians know when to monitor fetuses to effectively assess the developmental status of both ANS and specifically the vagus nerve. In addition, this paper will focus on which factors—i.e., fetal characteristics and behaviors, maternal lifestyle and pathologies, placental health and dysfunction, labor, incubator conditions, and drug exposure—may have an impact on the development of the vagus during the above-mentioned “critical window” and how. This analysis could help clinicians and stakeholders define precise guidelines for improving the management of fetuses and newborns, particularly to reduce the potential adverse environmental impacts on ANS development that may lead to persistent long-term consequences. Since the development of ANS and the vagus influence have been shown to be reflected in cardiac variability, this paper will rely in particular on studies using fetal heart rate variability (fHRV) to monitor the continued growth and health of both animal and human fetuses. In fact, fHRV is a non-invasive marker whose changes have been associated with ANS development, vagal modulation, systemic and neurological inflammatory reactions, and even fetal distress during labor.

## Introduction

The autonomic nervous system (ANS) is one of the main biological systems that regulates the body's physiology: in particular, the interaction between its two branches—the sympathetic nervous system (SNS) and the parasympathetic nervous system (PNS)—allows the organism to efficiently cope with endogenous and exogenous stressors (Goldstein, 2006; Bonaz et al., 2021).

Regarding the PNS, the vagus nerve has been recognized as a fundamental afferent pathway for sensing the milieu interior in peripheral tissues, thus allowing the organism to respond by activating complex networks involving the whole ANS, endocrine, metabolic, innate, and adaptive immune systems (Frasch et al., 2018b; Bonaz et al., 2021).

One of such responses, able to perform homeokinetic adjustments, is the cholinergic anti-inflammatory pathway (CAP). Briefly, through the vagus nerve, the brain receives information about the body's inflammatory milieu and, thus, activates neuroimmune responses to dampen the detected inflammation (Garzoni et al., 2013; Frasch, 2020; Bonaz et al., 2021). Very little is known, however, about the central representation, the information processing network, of the vagus nerve. An idea of neuroimmunological homunculus has been proposed and requires further research (Conway et al., 2006; Tracey, 2007; Diamond and Tracey, 2011; Frasch et al., 2018a; Castel et al., 2020).

The ANS regulatory capacity begins before birth as the sympathetic and parasympathetic activity contributes significantly to the fetus' development (Hoyer et al., 2017; Mulkey and du Plessis, 2018; Zizzo et al., 2020). Indeed, several studies have shown the vagus nerve is involved in many vital processes during fetal, perinatal, and postnatal life: from the regulation of inflammation through the CAP, which may affect each organ (Herry et al., 2019; Frasch, 2020), to the production of hormones involved in bioenergetic metabolism (Bystrova, 2009).

On the other hand, the ANS activity, as measured through heart rate variability (HRV)—a non-invasive biomarker evaluating the variability of the time intervals between successive heartbeats—is affected by the developmental and health conditions of both fetus and infant. Heart rate variability analysis can give paramount hints about the fetus' or newborn's well-being and allow clinicians to predict the occurrence of even deadly complications (Table 1) (Stone et al., 2013; Al-Shargabi et al., 2017; Massaro et al., 2017; Chiera et al., 2020; Frasch, 2020). Indeed, the first findings relating HRV alterations to fetal death, thus hinting to a potential predictive role of HRV, date back to the early sixties of the twentieth century (Hon and Lee, 1963).

**Table 1:** Summary of the HRV metrics cited in the main text (Brändle et al., 2015; Massaro et al., 2017; Oliveira et al., 2019; Patural et al., 2019; Frasch et al., 2020a).

**Parameter**	**Definition**
**TIME-DOMAIN**	
SDNN	Standard deviation of NN intervals. Related to both SNS and PNS activity and equivalent to Poincaré SD2.
RMSSD	Root mean square of consecutive RR interval differences. Related to vagal modulation and equivalent to Poincaré SD1.
**FREQUENCY-DOMAIN**	
LF	Low-frequency band: 0.04–0.2 Hz for newborns and 0.04–0.15 Hz for infants. It can be expressed as LF peak, LF power or LFn (normalized power in relation to total power). Related to both sympathetic and vagal modulation and baroreceptor reflexes.
HF	High-frequency band: 0.20–2.00 Hz for newborns and 0.20–1.40 Hz for infants. It can be expressed as HF peak, HF power or HFn (normalized power in relation to total power). Related to both vagal modulation and respiratory cycle.
LF/HF	Ratio of LF-to-HF power. Related to both SNS and PNS activity.
TP	Total power of the electrocardiography spectrogram.
**NON-LINEAR**	
SD1	Poincaré plot standard deviation perpendicular to the line of identity. Equivalent to RMSSD.
SD2	Poincaré plot standard deviation along the line of identity. Equivalent to SDNN.
CSI	Cardiac Sympathetic Index (ratio of SD2-to-SD1).
HRV Index	Number of all RR intervals divided by the number of RR intervals at the highest point of the RR histogram.
Parseval Index	Ratio between the square root of the sum of LF and HF powers and the value of SDNN
PE	Permutation entropy.
DFA α1	Detrended fluctuation analysis, which describes short-term fluctuations. Usually used to assess brain injury or pathology severity.
DFA α2	Detrended fluctuation analysis, which describes long-term fluctuations. Usually used to assess brain injury or pathology severity.
RMS1	Root mean square from detrended fluctuation analysis, which describes short-term fluctuations. Usually used to assess brain injury or pathology severity.
RMS2	Root mean square from detrended fluctuation analysis, which describes long-term fluctuations. Usually used to assess brain injury or pathology severity.

Nonetheless, the importance of ANS and vagus nerve regulation in fetal and neonatal development is still underrepresented (Sadler, 2012; Moore et al., 2016). Moreover, except the third trimester (Mulkey and du Plessis, 2018, 2019), literature usually lacks an analysis of potential “critical windows” in the perinatal period that may give information about ANS development. Defining such critical windows during gestation could help clinicians know exactly when to monitor fetuses to effectively assess the developmental status of both ANS and vagus nerve.

Indeed, several animal studies and even some human studies have shown that many factors (e.g., malnutrition, high level of stress, toxic exposure) during early, mid, and late gestation can impair brain and ANS development/regulation, thus inducing biological and behavioral alterations. Moreover, some studies have highlighted that the same factors can differently affect development based on the exposure period (i.e., early vs. late gestation) (Schulz, 2010; Gartstein and Skinner, 2018; Kasahara et al., 2020, 2021). As a consequence, by relying on appropriate animal models, several authors have defined potential critical windows related to human brain development (see, for instance, Figure 1 in Morrison et al., 2018).

Therefore, the present paper aims to review the fetal and perinatal development by focusing on the development of the ANS and, in particular, of the vagus nerve to identify possible critical windows that could impact their maturation. This paper will also focus on which factors—i.e., fetal characteristics and behaviors, maternal lifestyle and pathologies, placental health and dysfunction, labor, incubator conditions, and drug exposure—may affect the development of the vagus.

## The Fetal ANS Development: The Search of “Critical Windows”

As the ANS is controlled by several brain areas (e.g., amygdala, hypothalamus, insula, cingulate cortex, and several brainstem nuclei) that constitute the central autonomic network (CAN), paying attention to their development is fundamental for studying the fetal ANS. In the same way, due to the CAP, it is also relevant to review the development of the organs potentially connected to the vagus nerve (Sklerov et al., 2019; Frasch, 2020). As the vagus nerve affects the body metabolism in a pleiotropic manner, such a review could help better understand the networks underlying this effect and how to harness them for improving fetal and neonatal health (Castel et al., 2020).

**Figure 1:**
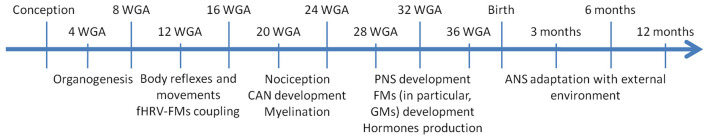
Timeline of fetal and postnatal development that shows the main events related to ANS development in order to define the main critical windows clinicians should pay attention to. ANS, autonomic nervous system; CAN, central autonomic network; fHRV, fetal heart rate variability; FM, fetal movement; GM, general movement; PNS, parasympathetic nervous system; WGA, weeks gestational age.

Since this review focuses on the role of ANS in human fetuses and newborns, the discussed time spans regarding gestational age are related to human development. As aids for the readers, Table 1 contains the meaning of the several HRV metrics cited in the following sections, whereas Figure 1 and Table 2 summarize the main findings of the current review.

**Table 2:** Summary of the finding of the present review regarding ANS development and HRV.

	**1st−2nd trimester**	**3rd trimester**	**Birth**	**Postnatal period**
**Physiological development**	The onset of fetal breathing movements increases RMSSD and HF power and decreases LH/HF power. Variations are also observed for SDNN, SDNN/RMSSD, LFn, and HFn. As behavioral states become more recognizable, fHRV-FMs coupling increases. From the 2nd trimester, HRV metrics analysis can estimate fetal autonomic brain age.	Vagus nerve myelination and development of vagal control by the nucleus ambiguus. Appearance of short-term variability in frequency-domain HRV metrics (e.g., HF) and reduction of long-term variability metrics (e.g., LF). fHRV patterns after VAS stimulation (after 32 WGA) or transabdominal fetal stimulation with halogen light can be used to assess ANS and vagal development. As revealed by specific metrics (e.g., AsymI, SLDE α, dlmax, dlmean, pL, sgridAND), *in utero* hypoxia decreases short-term predictability of ifHRV and increases its long-range similarity.	Steep rise in PNS tone around 37–38 WGA. Right before birth, SNS outflow from CAN greatly increases to support the fetus during labor. Before and after birth, the vagus and CAP functionality can be assessed through specific HRV metrics (e.g., AsymI, DFA α1, KLPE, and SDLE α). These metrics can also predict whether fetuses may recover after surgery and whether inflammation is under control.	Several HRV metrics (e.g., SDNN, CSI, HFn, LFn, TP, SD2, HRV Index, and Parseval Index) increase after birth, with the PNS modulation that takes predominance (e.g., lower LF/HF and higher RMSSD and HF) during the following 2 years. Measuring HRV during different sleep states helps assess ANS development: quiet sleep is characterized by PNS predominance and active sleep is characterized by SNS predominance. Social interactions, including skin-to-skin contact, increase ANS maturation.
**Environmental factors affecting ANS development**	Sex-specific differences in the non-linear properties of HRV, especially in the weeks before birth. Lower fHR, altered Fhrv, and more fHR decelerations, which point out to impaired vagal development, sympatho-vagal balance, and neurodevelopment can be due to: • maternal stress; • maternal pain (through higher cytokine production); • maternal malnutrition; • maternal sleep disorders (which reduce fetal breathing movements); • maternal pathologies (chronic hypoxia, infections, autoimmune, metabolic, neuroendocrine, and mood disorders); • placental dysfunction (due to neuroendocrine, immune, and cardiovascular alterations). Many fHRV are reduced by nicotine and/or alcohol exposure, starting from the 1st trimester. These perturbations last after birth, given the fact that alcohol, in particular, impairs CAN development. Pathogens such as Zika virus can hinder the communication between CAN and the cardiac pacemaker cells. Extreme temperature variations alter cerebral blood flow and, thus, fHRV. Noise, light, pollution, and EMF can affect brain and ANS development. Among drugs: • sGCs transiently induce SNS suppression, but can affect neurodevelopment (e.g., altered R-R interval variability lasting until adulthood); • in the 1st trimester, ACE inhibitors may induce cardiovascular and CNS malformations; • in the 3rd trimester, SSRIs reduce FMs and fHRV reactivity after VAS; • opioids increase many fHRV metrics throughout pregnancy and impair FMs and fetal breathing movements in the 3rd trimester; • prolonged administration of MgSO4, especially with sGCs, reduce fetal breathing movements and affect fHRV dose-dependently. IUGR reduces FMs and increases HR decelerations.		sOT may alter vagal control since it can bind to neurons in the vagal dorsal motor nucleus. Full lateral position of the mother in case of epidural analgesia seems to change fHRV less than wedged supine position. A difficult labor is revealed by alterations in fHR and fHRV, in particular, in PNS-related metrics. Compared to CS, spontaneous labor without analgesia increases PNS-related metrics and decreases SNS-related ones. Difficult delivery or CS without labor may induce hypoxia and oxidative stress that can cause cell death in the ANS brainstem centers. Hyperactive uterine contractions, perinatal hypoxia, and fetal acidemia greatly increase RMSSD and induce HR decelerations due to hypotensive pattern of pathological arterial blood pressure and cardiovascular decompensation. Preterm birth or lack of labor reduce cortisol release, which may alter the CAP development.	Sudden increase in RMSSD could pinpoint to an acute inflammatory response. Neuroinflammation alters global ANS functioning as revealed by entropic HRV metrics. Sepsis, NEC, and other diseases, which can be predicted by HRV metrics such as RR intervals, sample asymmetry and SampEn, impair ANS regulation centers through cytokines. Brain injury affects ANS postnatal adaptation, with several DFA metrics (i.e., α1, α2, RMS1, and RMS2) associated with specific brain injuries. Postnatal complications induce lower ANS tone. The incubator may also affect HRV: • warm incubators tend to increase SNS tone and reduce PNS activity, whereas the opposite is true for incubators 2°C colder than newborns' temperature; • excessive noise (>45 dB) can alter ANS development; • excessive light due to incubators not properly covered can heighten SNS tone; • EMF coming from the incubator power increases LF/HF and decreases HF; • lack of sleep due to medical procedures can dampen vagal modulation; • prone position increases PNS-related HRV metrics but decreases short-term variability. Supine and side position seem to induce more stable HR and oxygenation.
**Consequences of ANS development disruption**	Impairment in FMs and uncoupling between FMs and fHRV can reveal altered fetal sensorimotor capacities. Impaired vagal balance, as indexed by low PNS-related metrics (e.g., RMSSD), can induce alteration in stress response and inflammation regulation (impairment in CAP development). Impaired neurodevelopment and myelination and altered neurotransmitter signaling throughout the CNS. Lower non-linear HRV metrics reveal higher risk of morbidity and death during or after birth. Reduced fetal growth.		Several pathologic conditions: apnoea, hypoxemia, sleep disruption, decreased growth, immunity impairment, and neuroendocrine alterations. Impaired autonomic regulation and brain development revealed through DFA metrics, i.e., α1, α2, RMS1, and RMS2. Alteration in CAP development and difficulties in regulating postnatal inflammation (e.g., NEC) as revealed by lower RMSSD and altered non-linear metrics. Whereas low RMSSD, SDANN, and SDNN may reveal poor neurological development, it seems neurological complications (e.g., cerebral palsy) and behavioral alterations (e.g., autism) can be investigated (even predicted) by specific HRV patterns. HRV alteration (lower HR, SNS-, and PNS-related metrics) could also reveal risk of SIDS. Hypertension, metabolic impairment, immune disorders, depression, and behavioral disorders in infancy, adolescence, and adult life as revealed by HRV metrics too low or high compared to peers.	
**TECHNICAL CONSIDERATIONS REGARDING HRV ANALYSES**				
The reproducibility of HRV properties in a given physiological setting depends on various factors that—due to their variety—are often neglected when comparisons and generalizations are made, such as: 1) The biosensors used to acquire the raw signal (e.g., PPG, BCG, ECG), 2) The sampling rate of the signal (for best precision in capturing HRV, we advise 1,000 Hz for the derivation of the raw RRI signal), 3) Pre-processing pipeline steps (preceding actual HRV computation): a) RRI → HRV computation window length, 5 min is the standard, but this number varies widely in publications; b) Sliding window settings impact smoothing HRV dynamics; when several 5 min segments are analyzed and the HRV values averaged, this can be non-overlapping or overlapping to variable degrees, usually 50% overlap; c) Other filter settings such as frequency bands chosen to reflect VLF, LF, and HF bands of HRV or embedding dimensions, time delays, and time scales for non-linear metrics.				

*ACE, angiotensin-converting enzyme; α1, a short-term fluctuations metric in detrended fluctuation analysis; α2, a long-term fluctuations metric in detrended fluctuation analysis; ANS, autonomic nervous system; AsymI, multiscale time irreversibility asymmetry index; BCG, ballistocardiogram; CAN, central autonomic network; CAP, cholinergic anti-inflammatory pathway; CNS, central nervous system; CS, cesarean section; CSI, cardiac sympathetic index; dlmax, max length of diagonal structures in recurrence quantification analysis; dlmean, average length of the diagonal structures in recurrence quantification analysis; ECG, electrocardiogram; DFA, detrended fluctuation analysis; EMF, electromagnetic field; fHR, fetal heart rate; fHRV, fetal heart rate variability; FM, fetal movement; GM, general movement; HF, high frequency; HFn, high frequency normalized power; HR, heart rate; HRV, heart rate variability; IUGR, intrauterine growth restriction; KLPE, Kullback-Leibler permutation entropy; LF, low frequency; LFn, high frequency normalized power; NEC, necrotizing enterocolitis; pL, percentage of laminarity; PNS, parasympathetic nervous system; PPG, photoplethysmogram; RMSSD, root mean square of consecutive RR interval differences; RMS1, root mean square from detrended fluctuation analysis; RMS2, root mean square from detrended fluctuation analysis; RRI, R-R interval; SampEN, sample entropy; SDANN, standard deviation of the average NN; SDLE α, scale-dependent Lyapunov exponent slope; SDNN, standard deviation of NN intervals; SD2, Poincaré plot standard deviation along the line of identity; sGC, synthetic glucocorticoid; SIDS, sudden infant death syndrome; SNS, sympathetic nervous system; sOT, synthetic oxytocin; SSRI, selective serotonin reuptake inhibitor; TP, total power; VAS, vibroacoustic stimulation; VLF, very-low frequency; WGA, weeks gestational age*.

### A Review of ANS Development

#### The Development During the 1st Trimester

The nervous system differentiates from the ectoderm approximately around 3 weeks gestational age (WGA), when part of the ectoderm develops into the neural plate. At 4 WGA, neurulation occurs: the neural folds arise from the neural plate and begin to fuse in both cranial and caudal direction, thus forming the neural tube, that is, the future central nervous system (CNS). During neurulation, some neuroectodermal cells detach from the neural folds and differentiate into the neural crests (NCs), the specialized cells that will form the future ANS (Moore et al., 2016).

By 4 WGA, the forebrain, midbrain, and hindbrain (the primary brain vesicles) are already visible, as are the spinal ganglia that come from the NCs. In the next weeks, the nervous system rapidly grows: the five secondary brain vesicles, along with the four ventricles and the meninges form, while the glioblasts (the glial cells precursors) start to differentiate and migrate throughout the developing nervous system. The neural tube also begins to thin to form the central canal of the spinal cord (Moore et al., 2016).

During this period (4–8 WGA), from the NCs several derivatives arise: the dorsal root ganglia, the sympathetic ganglia, the renal, celiac, and intestinal ganglia and plexus, the afferent ganglia of cranial nerves, and the suprarenal medulla (Newbern, 2015; Moore et al., 2016). In particular, by 6 WGA, the first parasympathetic ganglia can be detected (Müller and O'Rahilly, 1989) and, by 10 WGA, the sympathetic trunks have appeared throughout the vertebral column (Kruepunga et al., 2021) and, apart from the colonic loop, the extrinsic innervation of the enteric nervous system (ENS) is complete (Kruepunga et al., 2020). Neural crest cells also contribute to spleen development (Barlow-Anacker et al., 2017). The spleen begins to develop at 5 WGA as a lobulated organ, appears clearly around 8 WGA, and initially functions as a hematopoietic center; then, during fetal development, it gradually loses both this role and its lobules (Moore et al., 2016; Hill, 2021a).

Among all the NC cells, the vagal NC cells are of particular interest due to their derivatives. Vagal NC cells emerge adjacent to the somites 1–7, rostral to the head NCs and caudal to the trunk and sacral ones, and are the ENS precursors (Hutchins et al., 2018; Simkin et al., 2019). But these cells also contribute to the formation of organs such as the heart (especially, the intrinsic cardiac nervous system), thyroid and parathyroids, thymus, lung, and pancreas ganglia. Vagal NC cells then give rise to the parasympathetic, sympathetic, and sensory ganglia (Hutchins et al., 2018; Simkin et al., 2019; Fedele and Brand, 2020).

Several animal studies in vertebrates and mammals show that vagal NC cells ablation heavily impairs the development and function of the aforementioned tissues. For example, neurons and glial cells in the pancreas do not appear and the thymus loses both its vasculature and its capacity of developing T-cells (Hutchins et al., 2018).

At 5–6 WGA, the 12 cranial nerves appear (Moore et al., 2016). As the parasympathetic system is constituted by the oculomotor, facial, glossopharyngeal, and vagus nerves, and since the trigeminal, facial, glossopharyngeal, spinal accessory, and vagus nerves lay the foundations for social interaction especially in newborns—these nerves are central to sensing environmental stimuli, including the human voice, and to carrying out behaviors such as head-turning, smiling, sucking, and swallowing—(Porges, 2011), the cranial nerves maturation may be paramount for an optimal ANS growth and, thus, for developing regulatory adaptive responses. Regarding the vagus nerve, at this stage, the dorsal motor nucleus is the predominantly active effector center (Porges, 2011).

At 4–6 WGA the primitive diaphragm appears (Hill, 2021d) and, by 7 WGA, the four chambers of the heart have formed, thus connecting the heart to the aorta and the pulmonary trunk. By 10 WGA, fetal circulation passively exchanges gas mainly with the placenta and breathing begins. As gestation proceeds, blood entering the pulmonary circulation increases (Morton and Brodsky, 2016; Tan and Lewandowski, 2020).

From 7 to 8 WGA gray and white matter differentiate and, by the end of the 1st trimester, after the embryo has become a fetus, it is possible to recognize all the main brain structures, including the choroid plexus, thalamus, hypothalamus, amygdala, basal ganglia, corpus callosum, brainstem nuclei, cerebellum, and the neural plate of the insula (Moore et al., 2016; Hill, 2021b). Around the transition between the 1st and 2nd trimester, even the circumventricular organs, in particular, the area postrema, appear: they are organs devoid of the brain-blood barrier that, hence, allow the brain to be directed informed about the biochemical status of the internal milieu (Gokozan et al., 2016).

Interestingly, at 6 WGA, the embryo begins to show spontaneous movements and can “respond” to touch and light, as the motor spinal nerve fibers have appeared around 4 WGA (Moore et al., 2016): indeed, at 8 WGA the embryo is capable of activating a spinal reflex in response to touch, and between 7 and 11 WGA all facial reflexes have developed (Borsani et al., 2019). At 10 WGA the corticospinal connectivity begins to develop, together with an increase in synaptogenesis, and, as a result, body reflexes in the upper limbs start to appear (Borsani et al., 2019). Since fetal movements (FMs) tend to synchronize with fetal heart rate variability (fHRV), their detection can give important insights on ANS development, even in the 1st trimester (DiPietro et al., 2007; Zizzo et al., 2020). From 12 to 13 WGA, fHRV-FMs coupling becomes heavily affected by external factors: therefore, its analysis could shed more light on how the fetus is affected by maternal and environmental factors (DiPietro et al., 2015).

During this period, yawning develops (Borsani et al., 2019). Since yawning has been related to thermoregulatory behaviors and the ability of “switching” between arousal and rest states, both of which are strictly dependent on ANS functionality, the detection of yawning at this early age could represent a useful indicator of ANS development or, as suggested, brainstem functionality (Walusinski, 2006, 2010, 2014; Gallup and Eldakar, 2013). This hypothesis could be reinforced by the evidence that yawning seems to rely on interoception, the inner sense that constantly monitors the internal milieu and for which the vagus nerve plays a central role, and to be processed by the CAN (in particular, insula, hypothalamus, locus coeruleus, and reticular activating system) (Walusinski, 2006). Lastly, yawning amount and duration seem to correlate with neuronal development in mammals (Gallup et al., 2016).

#### The Development During the 2nd Trimester

At the beginning of the 2nd trimester, the cerebral cortex creates more and more sulci and gyri to increase its surface area and allow brain development. In particular, the subplate (a central hub for cortical connectivity) becomes visible and begins to receive connection from the thalamus, basal forebrain, and brainstem. Around 17–19 WGA, the cerebellum development can be assessed through magnetic resonance imaging (Hill, 2021d), the corticospinal tracts toward the whole body appear, and at 20 WGA the spinothalamic tract is completed (Borsani et al., 2019).

At 18–19 WGA, nociceptive neurons and the typical hormonal response to pain appear (Borsani et al., 2019; Chen et al., 2021), thus hinting that primitive pain perception might begin at this stage. Although thalamocortical connections, which strongly increase around 20–22 WGA, are usually required for pain experience and the hemodynamic-behavioral responses to nociceptive stimuli are usually detected around 26 WGA (Verriotis et al., 2016; Chen et al., 2021), several authors argue that fetuses could feel pain at least from 12 WGA, as the first superficial thalamic-subplate connections seem to be functionally equivalent to the later thalamocortical ones (Derbyshire and Bockmann, 2020).

Around 20 WGA and for the rest of the 2nd trimester, myelination occurs (Borsani et al., 2019). From the NCs, neurilemma cells arise and form the myelin sheaths that wrap both the axons of somatic motor neurons and the preganglionic autonomic motor neurons (Moore et al., 2016). Also, fetal microglia from the bone marrow invade the CNS, while gray and white brain matters keep on developing: the cerebral lobes with all their sulci and gyri, cortexes such as the insula, the cerebellum, the pons, and many other structures increasingly resemble their adult counterparts (Moore et al., 2016). From 17 to 24 WGA, the spleen almost completely develops its reticular framework (Satoh et al., 2009; Hill, 2021a). Regarding the ANS, brainstem, and hypothalamic centers form several connections with the other CAN structures, that is, insula, anterior cingulate gyrus, hippocampus, hypothalamus, and amygdala (Mulkey and du Plessis, 2018; Schlatterer et al., 2021).

During the 2nd trimester, the body becomes covered with lanugo, a fine downy hair that plays a paramount role in neurodevelopment. Indeed, through lanugo, the flow of amniotic fluid and FMs stimulate the low-threshold unmyelinated C afferent fibers (Bystrova, 2009), which have been found to be essential for both nociception and interoception. This stimulation reaches the brainstem, in particular the nucleus of the solitary tract—the main afferent nucleus of the vagus nerve—but also other nuclei including the parabrachial ones, and then more cortical structures such as the hypothalamic paraventricular nucleus and the posterior insular cortex. As a result, several motor and neuroendocrine responses are induced: for instance, gastrointestinal and anabolic hormones such as cholecystokinin, insulin, and insulin-like growth factor-1 are released. On the other hand, the hypothalamic-pituitary-adrenals (HPA) axis and vagal activation become more finely tuned (Bystrova, 2009).

Regarding sensorimotor development, around 15 WGA a complex set of movements (e.g., breathing movements, general body movements, isolated limb, head, and neck movements, startle and twitch movements, and swallowing) are available, whereas, by 18 WGA, stable handedness can be detected. Then, as gestation progresses, the amount of movement, both spontaneous and in response to vibroacoustic stimuli, gradually increases: indeed, mothers begin to feel their babies' movements (Moore et al., 2016; Borsani et al., 2019). At about 18–20 WGA, rapid eye movements (REM) can be detected (Borsani et al., 2019) as well as quiet and active sleeping states (Dereymaeker et al., 2017), although fetal behavioral states do not show particular coherence with ANS tone (Brändle et al., 2015).

As several vital systems are forming around the end of the 2nd trimester (e.g., alveolar ductal development and surfactant secretion begin at 24 WGA), babies born prematurely before 26 WGA have a higher chance of dying or developing neurodevelopmental disability (Moore et al., 2016; Morton and Brodsky, 2016; Hill, 2021c). Nevertheless, neonatal intensive care units have improved so much in the years that babies born as early as 20–22 weeks can survive if properly treated (Moore et al., 2016).

It is worth noting that, from the 2nd trimester, HRV metrics can successfully estimate fetal autonomic brain age (Hoyer et al., 2012, 2013b, 2015, 2017, 2019). Interestingly, gestational age can also be precisely estimated by measuring the opening and closing time of fetal cardiac valves (Marzbanrad et al., 2017), finding that could highlight the contribution of intrinsic fHRV or, at least, heart activity, to global HRV.

#### The Development During the 3rd Trimester

At 26–29 WGA, fetuses become able to efficiently control body temperature (Moore et al., 2016). By 29 WGA, all the major corticospinal, spinothalamic, and spinocortical tracts as well their myelination are completed, whereas from 24 WGA functional connectivity patterns in the fetal brain are recognizable. Indeed, at 32 WGA the cerebral cortex resembles the adult one and, in the next week, synchronicity between the hemispheres develops, as well as complex connections among several brain areas and networks (Moore et al., 2016; Borsani et al., 2019). As the 3rd trimester advances, specific nociceptive event-related potentials in the brain increasingly develop until 35–37 WGA (Verriotis et al., 2016).

From 25 WGA until birth, the vagus nerve myelination, the vagal control by the nucleus ambiguus, and, thus, the PNS rapidly develop, as testified by the appearance of short-term variability in frequency-domain HRV metrics (e.g., HF) and the proportional reduction of long-term variability metrics (e.g., LF), more related to SNS and baroreflex functions (Mulkey and du Plessis, 2018, 2019; Schlatterer et al., 2021). In particular, a steep rise in PNS tone can be detected around 37–38 WGA and basal fetal cardiovascular function becomes more influenced by the PNS. Nevertheless, from 30 WGA, cortisol, thyroxine, and catecholamines levels rise to prepare the fetus for birth. In the case of preterm birth, the ANS remains predominantly affected by both the SNS and the primitive vagal dorsal motor nucleus, with consequences on the stress response regulation toward environmental stimuli (e.g., excessive tachycardia or bradycardia) (Morton and Brodsky, 2016; Mulkey et al., 2018; Mulkey and du Plessis, 2019).

Lastly, whereas general movements (GMs) tend to increase until 32 WGA and then decrease as birth approaches, facial movements increase more and more (Borsani et al., 2019). During the 3rd trimester, distinct fetal behavioral states (i.e., quiet sleep, active sleep, quiet awake, and active awake) become increasingly recognizable through the analysis of heart rate (HR), eye movement, and GMs, as corticothalamic and brainstem areas develop more complex interactions (Brändle et al., 2015; Borsani et al., 2019). Interestingly, the analysis of specific HRV metrics, for instance, SDNN (more related to global neuroautonomic regulation), RMSSD (more related to vagal modulation), and PE, can help identify the actual behavioral state and, therefore, detect potential problems in neurodevelopment whether incoherence between HRV and body movements is detected (Schneider et al., 2008; Brändle et al., 2015).

#### The Development During Labor

Birth, as the transition from fetal to neonatal physiology, is a major challenge for the fetus and requires an ANS able to efficiently adapt the cardiovascular, respiratory, thermoregulatory, and metabolic (e.g., glycemic regulation) systems to the new environment. For this reason, before birth neuroendocrine signaling (e.g., catecholamines, cortisol, thyroid hormones, and renin-angiotensin) surges, and SNS outflow from CAN (e.g., hypothalamus, cortical, and brainstem centers) greatly increases. During labor, however, the nervous system is highly vulnerable to injuries due to hypoxia, energy deprivation, or oxidative stress (Morton and Brodsky, 2016; Mulkey and du Plessis, 2018; Mulkey et al., 2021).

Compared to cesarean section (CS), spontaneous labor without analgesia seems to improve cardiovagal modulation as shown by HF increase and LF decrease, despite several HRV metrics tend to increase in the postnatal days regardless of delivery mode (Kozar et al., 2018). A well-developed ANS responds efficiently to umbilical cord clamping by inducing the fetus to commence spontaneous breathing. Otherwise, especially in case of a difficult delivery or CS without labor, the fetus may experience hypoxia and have difficulties in secreting pulmonary surfactant, thus developing respiratory pathologies that can backfire, in particular, on ANS brainstem centers through oxygen deprivation and hinder autonomic regulation (Hillman et al., 2012; Morton and Brodsky, 2016; Mulkey and du Plessis, 2018).

It is noteworthy that all the adaptations required to pass from intrauterine to extrauterine life seem to rely on cortisol, whose release is markedly reduced in case of preterm birth or lack of labor (Hillman et al., 2012; Morton and Brodsky, 2016). Since cortisol is an efferent agent in anti-inflammatory neural reflexes like the CAP (Bonaz et al., 2021), its reduced production may alter the CAP development itself. Indeed, low cortisol levels could fail in regulating postnatal inflammation, e.g., necrotizing enterocolitis (NEC), which could induce cell death in the vagal brainstem centers (Fritze et al., 2014).

#### The Development in the Postnatal Life

After labor, pulmonary vascular resistance decreases compared to systemic vascular resistance as lungs inflate and fetal circulation becomes active (Tan and Lewandowski, 2020), whereas the nervous system integrates sensory, motor, and regulatory functions in more complex behaviors (Moore et al., 2016). In particular, centers that are paramount to neurotransmitters (e.g. noradrenaline) biosynthesis and autonomic functions (e.g., temperature, breathing, and satiety regulation) including the area postrema greatly develop around birth (Gokozan et al., 2016).

As revealed by HRV analysis, the ANS constantly develops. Indeed, several metrics related to both SNS and PNS (e.g., SDNN, CSI, HFn, LFn, TP, SD2, HRV Index, and Parseval Index) change right after birth and in the following months, with the PNS modulation that takes predominance (e.g., lower LF/HF and higher RMSSD and HF) during the following 2 years (Oliveira et al., 2019; Patural et al., 2019) to better regulate the increasingly social interactions the infant encounters (Porges, 2011).

It is worth remembering that the two ANS branches are anything but antagonists: in fact, the regulation of many organic functions (Goldstein, 2006), including the immune and inflammatory responses (Bonaz et al., 2021), strictly depends on the synergy between the two branches. Should one of them be damaged, the other one alone could not regulate the body's homeostasis efficiently anymore.

At birth, the higher cortical networks controlling the ANS increase their connectivity (Schlatterer et al., 2021) and, in low-risk newborns at around 1 day of age, coherence between electrocortical activity and brainstem-mediated autonomic tone, especially parasympathetic, can be easily detected (Mulkey et al., 2021). Social interactions, including skin-to-skin contact between mother and newborns, help increase ANS maturation as well as stress responsivity and circadian rhythms, in particular in preterm infants (Ulmer Yaniv et al., 2021).

As newborns spend most of their time sleeping, ANS development correlates also with sleep development, with quiet sleep (absence of movements and slow rhythmic breathing) characterized by PNS predominance and active sleep (myoclonic twitching, facial, eye, and head movements, and irregular heart and breath rates) characterized by SNS predominance. Hence, measuring HRV during different sleep states can help better assess ANS development (Yiallourou et al., 2012; Dereymaeker et al., 2017).

### How Disruptions in “Critical Windows” Can Affect ANS Development

In the previous section, we described fetal development with particular attention to ANS, CAN, sensorimotor development, and some organs. We have shown how alterations in CAN activity and sensorimotor behaviors can give hints about the developing ANS function. Similar hints can also be gained by monitoring organ development due to the research regarding the CAP and the systemic interactions of the vagus nerve (Bonaz et al., 2021).

However, the intrinsic spatiotemporal complexity of ANS development and the complex species differences in the timelines of neural development from which inferences are often drawn to human development make it difficult to identify specific or exclusive critical windows, although we might point to particular periods: 4–8 WGA, due to organogenesis; 12 WGA, due to subplate and fHRV-FMs coupling appearance; 18–24 WGA, due to nociception, CAN, and spleen development and myelination; the 3rd trimester, due to PNS development and change in FMs and hormones production; and birth, due to ANS adaptation to interact directly with the external environment.

Therefore, the next two sections will discuss short-term and long-term consequences of disruptions in ANS development.

#### Short-Term Consequences

Fetal or birth complications affecting ANS development may have consequences on organic functions and mortality right after birth and in the following months.

Autonomic nervous system impairment occurring *in utero* due to congenital heart disease, chronic hypoxemia, fetal growth restriction, acidemia, the trial of labor, bacterial, and viral infections or maternal factors can lead to labor complications (even death) and prematurity (Mulkey and du Plessis, 2018). Since the ANS requires 37 weeks *in utero* to fully develop, preterm newborns can experience difficulties in regulating their internal milieu in the face of environmental factors (Praud et al., 2017). Moreover, preterm birth often involves invasive treatments (e.g., invasive respiratory support) or drugs that can stress even more the immature ANS. Indeed, ANS responsiveness seems to remain quite low even when preterm infants reach term-equivalent ages compared to full-term newborns (Mulkey et al., 2018; Patural et al., 2019; Schlatterer et al., 2021).

Health conditions may, however, affect postnatal ANS maturation more than gestational age at birth: in the 3 months after birth, infants with a higher number of postnatal complications (e.g., infections, patent ductus arteriosus requiring treatment, mechanical ventilation) show a lower ANS tone measured through DFA metrics, i.e., α1, α2, RMS1, and RMS2. Whereas, α1 differs already at birth, the other metrics show different trajectories after 30–40 days, hinting at different ANS development due to the experienced postnatal comorbidities (Schlatterer et al., 2021). Asymptomatic toddlers born to mothers infected with Zika virus (ZIKV) during pregnancy, show alterations in their non-linear HRV measures reflecting chaotic behavior and recurrence plot properties. These changes were suggested to result from the effects of chronic hypoxia—which ZIKV infection is known to cause—on the interactions between the ANS and the cardiac pacemaker cell development which generates intrinsic HRV (Frasch et al., 2020a; Herry et al., 2021).

It is widely known that comorbidities such as NEC or hypoxic-ischemic encephalopathy (HIE) can negatively affect ANS (Schlatterer et al., 2021). For instance, stroke can easily alter both sympathetic and parasympathetic tone, thus impairing the ANS regulatory capacities (Reich et al., 2019). Specific kinds of brain injuries seem also to specifically affect ANS: for instance, DFA revealed that white matter injury, watershed stroke, basal ganglia injury, or global injury can show characteristic HRV signatures (Metzler et al., 2017). Prematurity increases the risk of these adverse events (Mulkey and du Plessis, 2018). Fetal gut injury due to inflammation in a fetal sheep model of NEC etiology leaves a specific HRV signature and preterm neonates show abnormalities in HRV 1–3 days before clinical symptoms (Stone et al., 2013; Liu et al., 2016).

However, we have yet to fully understand ANS development, even after birth. Despite knowing that the CAN coordinates autonomic function and that left and right hemispheres modulate PNS and SNS tone, respectively (Mulkey et al., 2021), we have conflicting results on how the brain precisely controls ANS in newborns. For instance, whereas hypoxic-ischemic injuries in the right hemisphere seem to depress SNS tone, vaso-occlusive strokes in the same hemisphere appear to increase SNS tone. Whereas, vaso-occlusive strokes in the left hemisphere seem to increase PNS tone, hypoxic-ischemic injuries seem to have no effect on PNS tone. In some cases, insular injuries do not significantly change ANS tone; or, despite the effects on ANS tone, systemic hemodynamic meters (e.g., diastolic and systolic blood pressure) do not seem affected by brain injuries (Schneebaum Sender et al., 2017; Reich et al., 2019).

Many postnatal pathologies—e.g., hyperbilirubinemia, congenital heart disease, respiratory distress syndrome, respiratory syncytial virus, NEC, and sepsis—can lead to ANS impairment, probably due to inflammatory cytokines and toxins that can bypass the brain-blood barrier and induce neural death in areas paramount to ANS control (Fritze et al., 2014; Al-Shargabi et al., 2017; Bonaz et al., 2021).

As pathologies can affect ANS development, altered ANS function can make newborns more vulnerable to those same pathologies, due to failure in activating efficient anti-inflammatory reflexes including the CAP (Yiallourou et al., 2012; Mulkey et al., 2018; Bonaz et al., 2021). Indeed, dysfunctional CAP can fail in regulating inflammation, thus letting the immune system go unchecked and free to induce tissue damage, shock, and even death, whereas, on the other hand, a functional CAP can prevent microglia activation, thus showing a neuroprotective effect (Garzoni et al., 2013; Frasch et al., 2016).

Autonomic nervous system impairment, maybe through impaired cardiocirculatory and ventilatory regulation, can increase the risk of apparent life-threatening events or sudden infant death syndrome (SIDS) and, thus, of death in the first 6–12 months of postnatal life. Indeed, newborns that died from SIDS seemed to have brainstem and cerebellar impairments and to fail in arousing from sleep, which could highlight the importance of assessing HRV development during pregnancy (Yiallourou et al., 2012; Horne, 2018; Mulkey et al., 2018).

It is interesting that fetal ANS impairment can also have consequences on infant temperament at both 3 and 6 months (Howland et al., 2020).

#### Long-Term Consequences

Autonomic nervous system impairment may have consequences on the global development that persist during infancy, adolescence, and adult life.

Although preterm infants may reach good ANS maturation after 2–3 years (De Rogalski Landrot et al., 2007), ANS impairment tends to remain even later in life and to be accompanied by SNS and HPA regulation impairment, both during wakefulness and sleep (Haraldsdottir et al., 2018; Urfer-Maurer et al., 2018).

Autonomic nervous system dysfunction in the first year of life could have consequences on cardiovascular regulation even during adolescence, which makes paramount monitoring ANS development in the 1st and 2nd year to discover infants at risk. Indeed, cardiovascular regulation impairment could lead to higher mortality due to cardiovascular events (Patural et al., 2019). An immature ANS due to inflammation, prematurity, or fetal growth restriction can also lead to other pathologies such as type 2 diabetes (Mulkey and du Plessis, 2018). These and other metabolic or immune pathologies that could arise later in life might have their origin in alteration of CAP functioning, that is, in inflammation regulation by the ANS (Pavlov and Tracey, 2012; Burgio, 2015; Bonaz et al., 2021). Furthermore, ANS alterations in newborns can lead to neurological dysfunctions including cerebral palsy at 2 years of age (Dimitrijević et al., 2016).

Since ANS development is coupled with FMs development, ANS alterations can heavily affect CNS and neurobehavioral growth: on the one hand, FMs are usually considered the base for the fight-or-flight response, thus influencing the stress response (Schmidt et al., 2014); on the other hand, ANS immaturity correlates with altered cerebral and psychomotor development (Hoyer et al., 2014; Schmidt et al., 2014; Schneider et al., 2018), and even with altered cognitive, language, playing, and social skills (Doussard-Roosevelt et al., 1997, 2001; Bornstein et al., 2002; DiPietro et al., 2007; Siddiqui et al., 2017).

Autonomic nervous system immaturity due to fetal or postnatal complications (e.g., maternal pathologies, birth difficulties) could then impair the whole brain development and, therefore, behavior, stress response, and mood regulation, with negative consequences—even serious neurological or psychological pathologies—in infants, adolescent, and adult life (Mulkey et al., 2018; Mulkey and du Plessis, 2019). Even painful procedures during hospitalization seem to induce alteration in stress regulation and brain development in newborns (Holsti et al., 2006). Maternal stress during pregnancy alters fetal ANS by entraining fetal heart rate (fHR) with maternal HR decelerations during respiratory effort (Lobmaier et al., 2020). Eight-years old children with autism spectrum disorders (ASD), depression or conduct disorder showed, respectively, distinct signatures of HRV compared to controls and it has been proposed that, at least for ASD etiology, such abnormalities in HRV may be detected as early as *in utero* where they may be induced by asymptomatic inflammatory events altering ANS, among other systems (Frasch et al., 2019, 2021).

Due to the negative consequences ANS impairment can have later in life, it is paramount to rely on any protective factor that can sustain ANS maturation, starting from taking care of maternal health and lifestyle, which can greatly affect fetal development and, then, breastfeeding. In the same way, stress relief during pregnancy, skin-to-skin contact, and social interactions between newborns and parents/caregivers can help newborns tune their ANS, reduce stress, increase resilience, and improve their neural plasticity (Horne, 2018; Mulkey and du Plessis, 2018; Manzotti et al., 2019; Antonelli et al., 2021).

## The Environmental Factors Affecting the ANS Development

Whereas, in the previous section we described the fetal development in relation to ANS maturation, showing potential short-term and long-term alteration in case something should negatively affect pregnancy or labor, this section will now detail what factors can alter ANS development and in which ways.

### Prenatal Life Influence on the ANS

#### Fetal Factors

##### Incorrect Fetal Movements

Around 8–9 WGA, it is possible to clearly record GMs (Prechtl, 1990). General movements analysis can differentiate specific movement patterns, concerning a specific part of the fetal body, from non-specific movement patterns, involving more than one body segment (de Vries et al., 1982, 1985). General movements quality, as well as their variability and modulation in intensity and velocity, could be valid indicators of fetal neurodevelopment, in particular concerning supra-spinal control on motor activity (Moreira Neto and Porovic, 2018). Different methods could be used to evaluate the quantity and quality of FMs, such as mother perception, acceleration, and variability at cardiotocography (CTG), real-time ultrasound, myography, actocardiography (Zizzo et al., 2020), and the Non-stress Test, which relates FMs to fHR accelerations (Bryant et al., 2020).

Both gross motor activity and breathing FMs, particularly during early gestation, represent an important evaluative parameter for fetal neurobehavior and to predict future neurodevelopmental disorders. Indeed, respiratory sinus arrhythmia appears to be strictly related and modulated by efferent vagal activity. Fetal motor activity, therefore, results closely tied to breathing and cardiovascular patterns, which are modulated by the ANS (Zizzo et al., 2020).

According to a recent systematic analysis, FMs can affect the ANS as revealed through modifications in the time-domain and frequency-domain fHRV metrics. The onset of fetal breathing movements, in particular, induces a variation in the main vagal nerve activity parameters: RMSSD and HF power increase, while LH/HF power decreases. Variations are also observed for SDNN, SDNN/RMSSD, LFn, and HFn (Zizzo et al., 2020). In this sense, monitoring the quality and the evolutionary pattern of FMs could reveal useful insight regarding ANS, in particular, vagal maturation.

Fetal movements patterns could be limited as a consequence of an inappropriate ratio between fetal and uterine dimension (Shea et al., 2015; Verbruggen et al., 2018), as well as a consequence of an inadequate amount of amniotic fluid (Sival et al., 1990). Since we have seen that amniotic flow and fetal growth restriction can affect vagal development, their effects could then be mediated by FMs. Indeed, fetuses with intrauterine growth restriction (IUGR) frequently present an oxygenation impairment, with a decrease of breathing movements and GMs, and an increasing number of HR decelerations (Bekedam et al., 1991).

Fetal motor capacities, through the implied neuronal activity, is paramount for differentiation, migration, synaptogenesis, and development of neuronal networks. Environmental stimuli, *per se*, induce motor reactions, as the sensory system maturation proceeds. General movements allow fetuses to experience their bodies and the surrounding space, hence exploring the consequences their movements have on their bodies and environment (Fagard et al., 2018). In summary, the FMs make it possible to start developing a primitive body map. The repetitive movements, with the consequent repetitive sensory afferents induced, produce habituation in the fetus, that is manifested also in fHR changes: the ANS trains to adapt the body systems' response to environmental stimuli, even through the movements (Lecanuet et al., 1992; Kisilevsky and Low, 1998).

It is therefore legitimate to argue that a decreased possibility/capacity to express movements by the fetus, that is related to an impairment of the sensory stimulation, could interfere with the normal neurodevelopmental pattern of ANS and vagus nerve itself.

Maternal perception of FMs, in conclusion, is one of the factors that could influence in a relevant way the emotional state of the mother. Despite the fact that there are contrasting results about the sensitivity of fetal activity reported by the mother (Sorokin et al., 1982; Schmidt et al., 1984), it appears that there is an association between the increase of force and frequency of perceived movements and a reduced risk of stillbirth and negative outcomes (Heazell et al., 2018). In some cases, the healthcare providers recommend around 28 WGA the “count to 10” method: the mother counts the number of FMs every day, at the same hour, verifying that they occur not <10 times in a 2–3-h period (Bryant et al., 2020). Moreover, it seems that monitoring FMs could contribute to creating a primitive maternal–child bonding, even before birth (Flenady et al., 2019). Diminished perception of this motor activity, could therefore be worrisome for the mother, leading to an increased amount of stress, which in turn may have an impact on fetal neurodevelopment (Bryant et al., 2020).

##### Fetuses' Sex

A variety of different ANS and vagus nerve pattern activities are now well-established between different sexes, which can be well-represented by HRV tracking even during fetal life. Regarding vagal activity in fetuses, not all the authors agree in observing significative sex-related differences (Oguch and Steer, 1998; DiPietro et al., 2004; Lange et al., 2005; Bhide and Acharya, 2018), even when the same methodologies were used. The discrepancy between these studies may be partly due to the different experimental variables.

Male fetuses show a more reactive ANS and less complexity in control systems than female ones, with a more linear and significantly less complex fHR activity (Bernardes et al., 2008). Moreover, an exploratory study conducted on twins, highlighted that sex-specific differences in fetuses can be noticed both in linear and non-linear fHR metrics. This seems to be significantly influenced also by the combination between twins of different sexes, both in intrapair average and absolute differences (Tendais et al., 2015).

The regulatory patterns of sympathovagal balance show sex-specific differences as pregnancy progresses (Buss et al., 2009). During the 1st trimester, there are no significant differences in HRV track between male and female fetuses (McKenna et al., 2006). Both sexes demonstrate an evolutionary pattern with increased entropy indexes until 34 WGA, with a slight decrease after 37 WGA. With the passing of the weeks, however, sex-specific differences in the sympathovagal balance start to be detected. From 34th WGA, female fetuses show indeed a higher mean fHR and entropy, as well as a lower short-term variability and sympathovagal balance, compared to the males (Gonçalves et al., 2017). This could suggest a different developmental pattern of the vagus nerve, both sex- and time-specific.

In this sense, hormones may play a crucial etiological role, as literature shows that gonadal hormones have a significant impact on the sex-dependent differentiation of the entire nervous system (Chung and Auger, 2013). During pregnancy, the maturation pattern of fetal gonads must be taken into account. In the first weeks, these glands are undifferentiated between male and female fetuses and complete the cellular differentiation process around the 44th post-conceptional day (Baker and Scrimgeour, 1980). The production of testosterone in male fetuses begins during the 2nd month of pregnancy and reaches the maximum in the 2nd trimester (Chung and Auger, 2013), just before when vagal tone starts to rise (Mulkey and du Plessis, 2019).

The different patterns of ANS development could also be due both to a different sex-specific susceptibility to environmental stimuli and a different neurodevelopmental trajectory of male and female fetuses, which could lead to sex-specific developmental intervals of maximum susceptibility to environmental exposures (Buss et al., 2009; Bale, 2016).

Moreover, considering the role of the vagus nerve in the CAP, which involves immune cells such as macrophages (Borovikova et al., 2000; Tracey, 2007; Fairchild et al., 2010; Frasch et al., 2016), and the sex-related neurodevelopment impact of microglial functions (Hanamsagar and Bilbo, 2016), the different characteristics of the developmental pattern of vagus nerve between male and female fetuses could predispose for sex-specific susceptibility and outcomes in many neurodevelopmental disorders (Berntson and Cacioppo, 2004).

#### Maternal Factors

##### Maternal Distress

The definition of stress has evolved through time, but it is now accepted that a stressor is an uncontrollable environmental demand that exceeds the physiological imbalance of the organism (Selye, 1950; Koolhaas et al., 2011). Several uncontrollable situations such as natural disasters, relational or financial problems, bereavement, and/or stressful daily hassles can threaten a woman's life during her pregnancy. These situations increase the risk of impairment of fetal brain development resulting in emotional, behavioral, and/or cognitive problems in later life (Glover, 2015; Antonelli et al., 2021).

Historically, research findings oriented to understand the mediators that underlie the basis of fetal programming pointed out the HPA axis disregarding the role of ANS. However, more recently, several papers brought to light the importance of the ANS highlighting the fact that the early developmental disruption of the connections between the ANS and the limbic system might restrict the capacity of the ANS to respond to the environment and can be later implicated in neuropsychiatric disorders of the infant (Montagna and Nosarti, 2016; Frasch et al., 2018a, 2020b). Since, according to the Polyvagal theory (Beauchaine et al., 2007), the social/emotional development is related to the vagal system, any insult such as maternal distress suffered during the autonomic trajectory development will interfere with the maturation of the cerebral cortices structures and of the ANS disrupting the development of the Social Engagement System of the child (Porges and Furman, 2011).

Moreover, an impairment in vagal balance, particularly a decreased tone, might be implicated in depression, anxiety, post-traumatic stress disorder, and schizophrenia (Thayer and Brosschot, 2005). We have recently demonstrated that maternal stress affects the coupling of maternal HR to fHR showing that the fetuses of stressed mothers show significant decreases in fHR, probably representing a fetal stress memory that may serve as a novel non-invasive biomarker (Lobmaier et al., 2020).

##### Maternal Pain

Maternal pain during pregnancy is a symptom that should be seriously taken into account by clinicians, since it could underlie severe pathological conditions, which, in turn, can lead to negative outcomes, both for the mother and the fetus (Brown and Johnston, 2013). Many pathologies, even pre-existing before conception, may induce an acute exacerbation of the chronic pain during the pregnancy, including rheumatoid arthritis, sickle cell disease, Ehlers-Danlos syndrome, and vulvodynia, tuberous sclerosis, and irritable bowel syndrome (Ray-Griffith et al., 2018).

The most frequently observed symptoms are: low back pain, headache, pelvic girdle pain (PGP), leg cramps, breast tenderness, abdominal pain, and ligament pain (Davis, 1996; Jarrell, 2017; Lutterodt et al., 2019). Some symptoms, such as PGP and low back pain, could be physiological consequences of the musculoskeletal adaptations of the maternal body, due to an increasing uterine volume (Davis, 1996; Vermani et al., 2010; Casagrande et al., 2015; Sehmbi et al., 2017; Lutterodt et al., 2019). This could be a potential causing factor of the time-dependent onset of pain symptoms during the pregnancy.

Continued pain and discomfort throughout the pregnancy can induce both a sensitization of the maternal nervous system, with a lower pain threshold (Gintzler, 1980; Cogan and Spinnato, 1986; Eid et al., 2019), and a dysregulation in the inflammatory response, mediated by the CAP via the vagus nerve (Garzoni et al., 2013).

The patterns of cytokine response and the neuroimmune physiology of the fetus and the placenta mirror the maternal ones throughout pregnancy (Sherer et al., 2017, 2018; Prins et al., 2018; Peterson et al., 2020). These patterns are altered by pain and could hence not only be pre-emptive for negative outcomes for labor and birth (Goldenberg et al., 2008), but also suggest their influence on the neurodevelopment of the fetal vagal system itself (Borovikova et al., 2000; Tracey, 2009; Frasch et al., 2016).

The maternal pain perception, moreover, can undermine her emotional state, contributing to increased stress and worries (Persson et al., 2013; Clarkson and Adams, 2018; Mackenzie et al., 2018; Lutterodt et al., 2019), and this could additionally interfere with the vagus nerve development of the fetus.

Despite the evidence of positive effects of non-surgical and non-pharmacological treatments, such as physiotherapeutic interventions, acupuncture, or osteopathic manipulations (Gutke et al., 2015; Gallo-Padilla et al., 2016; Ray-Griffith et al., 2018; Smith et al., 2018; Cerritelli et al., 2020), the use of drugs for pain relief (e.g., low-dose aspirin, acetaminophen, non-steroidal anti-inflammatory drugs, and opioids) during pregnancy is still very common (Ray-Griffith et al., 2018). These therapies can affect fetal neurodevelopment: the increase prescriptions for opioids during pregnancy, for example, correlates to an increased incidence of neonatal abstinence syndrome (NAS), resulting in sleeping and feeding difficulties, as well as a dysregulation in newborns' CNS (Pryor et al., 2017; Ray-Griffith et al., 2018). Hence, further research is needed about these drugs' impact on fetal vagus nerve development.

##### Maternal Nutrition, Microbiota, and Metabolic Pathologies

Maternal nutrition is a substantial part of the “developmental or metabolic programming” of newborns (Koletzko et al., 2018, 2019), with short- and long-term consequences on health and a relevant impact on fetoplacental growth patterns (Morrison and Regnault, 2016).

The inadequate intake or absorption of nutrients, such as polyunsaturated fatty acids, folic acid, and vitamin B12, may result in altered fetal neurodevelopment (Starling et al., 2015; Wang et al., 2015), and lead to important consequences on neurobehaviour during infancy (Gernand et al., 2016; Bordeleau et al., 2021). Maternal malnutrition has an impact also on the endocrine system development: the fetus is overexposed to maternal cortisol, due to the lack of placental control systems (Allen, 2001; Seckl and Meaney, 2004), and the fetal HPA axis can be impaired, possibly leading to a dysregulation of sympathovagal balance and inflammatory set point (Christian and Stewart, 2010).

Even if further research is needed, maternal vitamin D intake deficit correlates with altered metabolic and contractile development of the heart and the control mechanisms of blood pressure in fetuses (Morris et al., 1995; Gale et al., 2008). Similarly, calcium, zinc, and iron assumption during pregnancy seems to affect fetal cardiovascular development and autonomic regulatory function (Christian and Stewart, 2010). Therefore, it may be assumed that vagus nerve development itself could be affected by maternal malnutrition.

Inadequate maternal nutrition could also lead to excessive weight gain and gestational diabetes, with negative outcomes for the fetus (AlSaif et al., 2015; Edlow, 2017; Peterson et al., 2020; Tong and Kalish, 2020). Maternal obesity, in particular, both before conception and during pregnancy, is associated with impaired fHRV and poorer ANS development (Christifano et al., 2020).

Maternal metabolic dysregulation seems to be related to gut dysbiosis (Hasain et al., 2020; Tong and Kalish, 2020) and, thanks to the direct link due to the feto-placenta-maternal unit (Nuriel-Ohayon et al., 2016; Laursen et al., 2017; Bordeleau et al., 2021), it could negatively influence the development of fetal microbiota (Zhou and Xiao, 2018; Tong and Kalish, 2020). Maternal metabolic dysfunctions and dysbiosis can affect the systemic inflammatory response of the mother and the fetus (Hasain et al., 2020; Peterson et al., 2020). Moreover, considering the correlation between the gut-brain-microbiome axis and immune system via neuroendocrine signaling (Aidy et al., 2015; Vitetta et al., 2018; Fuhler, 2020), these maternal affections can affect fetal neurodevelopment and potentially lead to adverse health outcomes later in life (Borre et al., 2014; Abdel-Haq et al., 2018; Tong and Kalish, 2020; Bordeleau et al., 2021).

Lastly, this feto-maternal pro-inflammatory state could interfere with the set point of the fetal vagal regulatory system through CAP dysregulation, thus impairing fetal intestinal permeability and integrity (Garzoni et al., 2013).

##### Circadian Clock and Vagal Activity

During the 3rd trimester of pregnancy, emerging fetal sleep states are detectable, with the onset of the functional organization of the sleep cycle around 28–30 WGA, and these patterns gradually consolidate around 36 WGA (Van den Bergh and Mulder, 2012). At this developmental stage, the fetus shows alternating behavioral states, passing through non-REM sleep and REM sleep, which lasts about 70–90 min (Visser et al., 1992), with usually a wake state duration under 10%.

Sleep cycles are essential for fetal neurodevelopment (Graven and Browne, 2008). The deprivation of the REM sleep phase during uterine life can lead to altered cortical and brainstem development and to the supersensitivity to the noradrenaline of the pyramidal cells in the hippocampus, impairing the receptors' sensitivity (Mirmiran et al., 2003). Consequently, the fetal and infant capacity to homeostatically adapt to the environment could be impaired (Van den Bergh and Mulder, 2012).

Fetal heart rate patterns, together with GMs and REM recording, are the main factors to define the passage through fetal sleep states (Van den Bergh and Mulder, 2012), pointing out the direct relationship between vagal activity and circadian rhythm.

Although the ANS and vagus nerve activity appear to have an endogenous circadian rhythm (Hilton et al., 2000; Kentish et al., 2013), with a pacemaker system that regulates its behavior regardless of sleep-awake states (Malpas and Purdie, 1990; Hilton et al., 2000), a circadian variation pattern of vagal activity can be recorded via HR variations.

The circadian dynamics of fHR is synchronized with the maternal rhythm of the activity at rest, HR, cortisol, melatonin, and body temperature. It is possible, therefore, to assume that the mother, considering the limited light-dark variation during intrauterine life, entrains the developing circadian rhythm of the fetus to this cycle (Mirmiran et al., 2003). In this sense, dysregulations in mother circadian rhythm, due to disturbed sleep-awake patterns, could interfere with the development of circadian control systems of the fetus and, consequently, with the circadian profile of the vagus nerve, impacting the physiologic vagal neurodevelopment itself.

Several factors can normally affect the sleep states of the mother during pregnancy: the hormonal, biomechanical, and behavioral changes that occur during the three trimesters, often lead to increased sleep disturbances, particularly in the last trimester (Sahota et al., 2003; Pien and Schwab, 2004; Facco et al., 2010; Mindell et al., 2015).

It has been reported in these cases a correlation with deficit in fetal growth, adverse outcomes for labor and birth, and even preterm birth (Lee and Gay, 2004; Micheli et al., 2011; Zafarghandi et al., 2012; Warland et al., 2018). Especially in the 3rd trimester, it is frequently reported the onset of sleep-disordered breathing (SDB) (Santiago et al., 2001; Edwards et al., 2002; Pien and Schwab, 2004; Venkata and Venkateshiah, 2009). Sleep-disordered breathing can disturb not only the physiological patterns of maternal sleep, but it could also lead to nocturnal hypoxia, which produces an enhanced activation of the maternal ANS (Pien and Schwab, 2004; Sahin et al., 2008). There are also repercussions on the fetus, in response to these apneic events: Fetal heart rate decelerations and decreased breathing movements, which could affect neonates' neurobehavior, even if currently there are not enough data to support definitive conclusions about long-term outcomes (Pien and Schwab, 2004).

Lastly, maternal sleep deprivation seems to be related to the depressive state (Jarczok et al., 2018) and leads to increased systemic inflammation, with the increment in the production of pro-inflammatory serum cytokines (Chang et al., 2010). In this context, further research is needed to better clarify the relationship between maternal neuroendocrine dysregulation, due to chronobiological impairments, and the impact on fetal neurodevelopment, particularly on the CAP.

##### Maternal Diseases

Maternal pathologies influence the onset of altered neurodevelopment patterns during fetal life, which could lead to actual neurologic disorders during childhood (Faa et al., 2016; Vohr et al., 2017). In particular, metabolic and cardiovascular pathologies, like obesity, diabetes, hypertension, and preeclampsia, can disrupt vagus nerve development (Brown et al., 2008; Russell et al., 2016; Moors et al., 2020).

The pathologies that induce chronic hypoxemia in the mother, including SBD (Pien and Schwab, 2004) and anemia, may induce dysregulations in the vagal control systems. Such hypoxic states can also be secondary to infections, including chorioamnionitis, leading to several hemodynamic abnormalities, and cardiovascular compromise (Frasch, 2018). The endotoxemia, through lipopolysaccharide molecules, seems to interfere with the cardiac pacemaker synchronization system, altering the fHRV pattern (Frasch and Giussani, 2020).

Many viral infections, such as cytomegalovirus and other TORCH organisms or ZIKV infection, can lead to preterm birth and induce severe neurodevelopmental alterations in the fetus (Silasi et al., 2015; Vohr et al., 2017). These pathologies are associated with fetal inflammatory response syndrome, which manifests as increased pro-inflammatory markers, including IL-6, IL-1, and TNF-α (Gotsch et al., 2007). As a result, oligodendrocytes and their progenitors are damaged during a critical period of brain development, thus altering brain myelination and leading even to periventricular leukomalacia (Kadhim et al., 2001). The increase in cytokine production consequent to the inflammatory response can enhance the risk for cardiovascular disease, impairing CAN, autonomic reactivity (Harrison et al., 2013), and, therefore, affecting the vagal development. Moreover, a recent pilot study described that *in utero* ZIKV-affected babies display HRV metrics abnormalities. Even early prenatal exposure to the virus, indeed, seems to create a chronic hypoxic environment to the fetus, affecting vagus nerve developmental patterns, and imprinting postnatally the ANS cardiac pacemaker activity (Herry et al., 2021).

The implications of these infection-associated immunological events on fetal neurodevelopment seem to be time-dependent, thus supporting the clinical relevance of carefully evaluating the interfering factors throughout the entire pregnancy (Meyer et al., 2007).

Similarly, even autoimmune diseases can involve a dysregulation in maternal inflammatory response (Gordon, 2004; Lu-Culligan and Iwasaki, 2020; Han et al., 2021). Systemic lupus erythematosus and antiphospholipid antibody syndrome, for instance, entail the presence of IgG antiphospholipid antibodies, which can cross the placenta, interfering with the brain development and impacting the cardiovascular system of the fetus (Buyon, 1998; Nalli et al., 2017). These pathologies can dysregulate the neuroendocrine axis, altering, in particular, the cortisol production (Wilder, 1998; Sheng et al., 2020) and, thus, affecting sympathovagal balance development.

Even conditions that induce secondary hypertension or HPA dysregulation, such as pheochromocytoma, hyperaldosteronism, or Cushing syndrome, can affect fetal neurodevelopment (Morsi et al., 2018; Corsello and Paragliola, 2019).

Lastly, maternal mood disorders and depression seem to strongly impact the fetal neuroendocrine systems, in particular the amygdala (McEwen et al., 2016) and the hippocampus (Nemoda et al., 2015), strongly involved in the CAN (Harrison et al., 2013; Thome et al., 2017). Moreover, maternal psychopathology influences HRV tracking also after birth, showing a higher mean HR and lower vagal modulation and confirming the impact of this prenatal disorder on vagal neurodevelopment (Dierckx et al., 2009).

##### Nicotine/Alcohol Exposure

Smoke and alcohol exposure can induce in premature newborns higher sympathetic function, lower parasympathetic function, and had less cardiac autonomic adaptability (Mulkey and du Plessis, 2019). During the 1st trimester, there are significant negative correlations between the amount of maternal cigarette smoking and fHRV metrics, highlighting the disrupted temporal organization of autonomic regulation before birth (Zeskind and Gingras, 2006; Kapaya et al., 2015).

Prenatal nicotine exposure (PNE) is associated with higher systolic blood pressure and altered HRV after birth (Nordenstam et al., 2019). Furthermore, the diminished vagal cardiovascular capacity to respond to hypoxic and hypercapnic states in the fetus, due to the abolishment of the serotonergic neurotransmission to cardiac vagal neurons caused by PNE, has been hypothesized as a likely link to SIDS (Kamendi et al., 2006, 2009).

The effects of prenatal alcohol exposure (PAE) on fetuses are collectively known as fetal alcohol spectrum disorders (FASD). Of the various organ systems affected by FASD, the brain is the most severely impacted: even if the hippocampus seems to be spared, other CAN structures show an impaired development, including the thalamus, hypothalamus, and prefrontal cortex, and also the spinal cord (Caputo et al., 2016).

Alcohol easily crosses the placenta and can disrupt maternal–fetal hormonal interactions, leading to long-term impairments on neuroendocrine and immune competence (Zhang et al., 2005). Prenatal alcohol exposure also impacts the limbic-HPA axis development and functioning, leading to disrupted cortisol response (Ouellet-Morin et al., 2011). Prenatal alcohol exposure, therefore, induces a dysregulated fetal immune response, increasing the risk for infections, chorioamnionitis, and/or placental abruption, which can favor premature delivery (Reid et al., 2019). The consequences on the long-term capacity of CAP inflammatory response are worthy of further investigations.

Lastly, early pain reactivity measured through HRV in alcohol-exposed newborns seems to be blunted, suggesting different responsiveness to environmental stimuli even after birth (Oberlander et al., 2010). This seems to confirm the PAE effects on HPA axis stress reactivity, possibly via changes in central serotonin levels and/or HPA axis functions, and on the vagus nerve development itself (Haley et al., 2006).

#### Placenta

##### Placental Dysfunctions

A growing body of evidence suggests the placenta's role in modulating fetal development. The placenta can be defined as a neuro-immune-endocrine secretive organ, with the essential role of conveying nutrients and developing modulatory signals to fetuses, limiting their exposure to any factors that could alter their physiologic developmental patterns.

Many factors can affect placental physiology, such as maternal nutrition, nicotine or alcohol exposure, drugs, pathologies, and stress (Nugent and Bale, 2015). This results in an overexposure of the fetus to maternal cortisol, that in turn may produce an overactive fetal cortisol response and may be a predisposing factor to develop disorders related to elevated HPA axis activity, which could lead to impairments in environmental stimuli adaptability by ANS in newborns (Nugent and Bale, 2015).

The dysregulation of placental functions can induce an insufficient intake of nutrients for the fetus, thus, especially during mid and late gestation, impairing the development of various brain areas, including the hypothalamus that seems to be particularly sensitive to the nutrient deficiency (Nugent and Bale, 2015).

Moreover, through the production of corticotropin-releasing hormone (CRH), the placenta stimulates adrenocorticotropic hormone release in the anterior pituitary of the fetus, affecting both fetal HPA axis and amygdala development (Seckl and Meaney, 2004). A dysregulation in CRH production, secondary to various maternal conditions, such as hypoxia, increased inflammatory cytokines and glucocorticoids, stress, preeclampsia, and eclampsia, may lead to altered neurodevelopmental patterns of these fetal structures (Allen, 2001; Bale, 2016; Sheng et al., 2020). It could also affect the role of placenta as the immune pacemaker of pregnancy, increasing the risk of preterm birth (Allen, 2001; Peterson et al., 2020) and thus impairing vagal development during the last weeks of gestation.

Recently, placenta calcifications samples collected at delivery have been speculated to act as a memory of prenatal maternal stress and diseases exposure, hence highlighting the potential role of placenta in regulating the offspring's future cardiovascular and metabolic health, even through the *in utero* modulation of the developing ANS (Wallingford et al., 2018).

In addition, even mild inflammatory states in the mother might affect the placental conversion of maternal tryptophan to serotonin upstream of the fetal brain (Goeden et al., 2016). This affection ultimately interferes with cerebral fetal neurodevelopment, including the nucleus accumbens and hippocampus, that are involved in CAN (Seckl and Meaney, 2004).

In conclusion, many diseases can impact the placental function, which in turn seems to have a role in regulating the neurodevelopment of the main actors that interact with the fetal vagus nerve, and the monitoring of placenta secretome could be a useful biomarker of diseases and potential developmental disruptions of the ANS in the fetus (Aplin et al., 2020).

##### Placental Microbiome

Although the womb was usually considered sterile, recently it has been suggested that the placenta harbors a unique microbiome, composed of non-pathogenic commensal microbes (Aagaard et al., 2014). The placental microbiota seems to display an evolutionary pattern during pregnancy and appears to be diversified in the various areas of the placenta (Aagaard et al., 2014; Cao et al., 2014; Pelzer et al., 2017).

Many authors substantiated the materno-fetal transmission of the microbiome through the placenta during prenatal life, identifying the presence of the microbiota both in the peripartum placenta (Zheng et al., 2015; Pelzer et al., 2017) and in the meconium (Walker et al., 2017), partially regardless of the delivery mode.

Placenta dysbiosis can be related to antepartum infections (Aagaard et al., 2014; Doyle et al., 2017; Pelzer et al., 2017), maternal nutrition (Aagaard et al., 2014), maternal weight gain (Antony et al., 2015; Zheng et al., 2015; Benny et al., 2019), gestational diabetes mellitus (Bassols et al., 2016; Pelzer et al., 2017), use of probiotics/antibiotics (Pelzer et al., 2017), and periodontal pathogens (Fischer et al., 2019), and can lead to several adverse outcomes, such as preeclampsia and chorioamnionitis (Amarasekara et al., 2015; Doyle et al., 2017; Fischer et al., 2019; Olaniyi et al., 2020).

Moreover, it has been observed a correlation between placenta dysbiosis and preterm birth (Aagaard et al., 2014; Antony et al., 2015; Bassols et al., 2016; Prince et al., 2016; Pelzer et al., 2017; Fischer et al., 2019). More than the infections themselves, it would seem it is the dysregulation of the placental inflammatory response that could affect the production of placental CRH and stimulate the fetal HPA axis, leading to preterm labor and birth (Parris et al., 2021). The high immunoreactivity of the premature infants' gut could also lead to neurodevelopmental disorders (Cao et al., 2014).

The fetoplacental transmission of the placental microbiome may occur through the ingestion of the amniotic fluid and via umbilical cord blood supply (Cao et al., 2014; Walker et al., 2017). The matches collected between the placental and fetal gut and lung microbiomes seem to be associated with neonatal airway complications and enterocolitis (Al Alam et al., 2020; Parris et al., 2021), possibly affecting the ANS-mediated control mechanisms of these systems. Since the placental microbiome could also affect metabolic pathways, thus leading to a pro-inflammatory environment (Gomez-Arango et al., 2017; Fischer et al., 2019), it could also affect the ANS and vagus development of the fetus.

Despite this evidence, other authors claim that placental microbiome does not exist—the aforementioned results would be just due to methodological errors—or that there is only extremely low biomass, probably leading to minor clinical effects (Lauder et al., 2016; Leiby et al., 2018; Kuperman et al., 2020; Parris et al., 2021).

Nevertheless, the placental microbiome could be an interesting factor to be taken into account by the clinicians as a potential regulator of vagus nerve development.

#### Exogenous (Non-maternal) Factors

##### Environmental Stimuli

The variations in ambient and core temperature appear to impact newborns HRV (Massaro et al., 2017): in particular, extreme variations seem to affect the autoregulatory capacities of the cardiac system and, consequently, HRV, possibly due to a dysregulated release of excitatory neurotransmitters (Tsuda et al., 2018). Particularly warmer- or colder-than-average temperatures seem to be associated with increased systemic inflammation and alteration in placental blood flow (Martens et al., 2019; Sun et al., 2019), leading to dysregulation of metabolic and immune substrates of the fetus, and possibly influencing the regulatory functions of ANS.

Exposure to acoustic stimuli, through vibroacoustic stimulation (VAS), is one of the most used benchmarks for the assessment of fetal distress, since VAS induces a somato-cardiac effect mediated by the fetal behavioral states (Busnel et al., 1992). This cardiac-orienting response does not seem to be detectable before the functional maturity of the fetal autonomic system, which occurs around 32 WGA (Krueger and Garvan, 2019). The fetal responsiveness recorded via fHRV patterns after VAS stimulation (Abrams and Gerhardt, 2000) is refined as the pregnancy proceeds, being a useful parameter to evaluate the maturation of ANS and vagus nerve (Buss et al., 2009). As the nervous system of the fetus develops, around 36–39 WGA a different fHRV pattern is detectable according to different acoustic stimuli, as for example human speech (Lecanuet et al., 1992; Krueger and Garvan, 2019).

Even if the light-dark cycle does not seem to significantly affect the fetal circadian rhythm, there are age-dependent fHRV patterns after transabdominal fetal stimulation with halogen light, similarly to the VAS (Peleg and Goldman, 1980), which seem increasingly definite in the later stages of pregnancy, when the fetal optic pathways and ANS control on the cardiac activity reach maturity (Thanaboonyawat et al., 2006).

The persistent exposure to photic stimuli or high noise during pregnancy, for instance, due to the mother's occupation, seems to correlate with reduced fetal growth (Selander et al., 2019), increased incidence of congenital heart diseases (Gong et al., 2017), impaired development of cortical areas (Zhang et al., 1992; Wilson et al., 2008), and potential disruption of the vagus nerve maturation.

The occupational and environmental exposure to pollution may be another important disrupting factor for fetal ANS development. A growing body of evidence suggests that chemicals exposure can impact fetal growth (Dejmek et al., 1999; Snijder et al., 2012; Desrosiers et al., 2015) and is a risk factor for congenital heart diseases (Gong et al., 2017), preterm birth and severe neurodevelopmental abnormalities (Zhang et al., 1992; Lacasaña et al., 2006; Langlois et al., 2012; Yurdakök, 2012). Even if the effects on the vagus nerve are still to be defined, the hazards exposure may affect the fetal motor activity (DiPietro et al., 2014) and be related to delayed neurobehavioural development in neonates (Handal et al., 2008). It is instead well known that methylmercury from seafood impairs the higher centers of cardiac autonomic function (Karita et al., 2018), leading to severe ANS abnormalities (Sørensen et al., 1999; Grandjean et al., 2004; Murata et al., 2006; Gribble et al., 2015).

Lastly, even electromagnetic field (EMF) radiations during pregnancy, due to overexposure to mobile phones, diagnostic instruments, or occupation, seem to be a risk factor for preterm delivery (Roşu et al., 2016) and to have effects on ANS development (Rezk et al., 2008).

##### Drugs During Pregnancy and Labor

In the course of pregnancy, mother and fetus could undergo a variety of drug administrations, many of which can cross the placenta affecting fetal development in several ways (Miljković et al., 2001).

###### Drugs to Support Fetal Growth

Synthetic glucocorticoids (sGCs) are the therapy of choice to support fetal maturation for the risk of preterm delivery. Usually, it is administered between 24 and 34 WGA, mainly to ensure optimal fetal pulmonary development (Mulder et al., 2004; Committee on Fetus and Newborn and Section on Anesthesiology and Pain Medicine, 2016).

As excessive maternal cortisol, sGCs override the placenta mediator system (Sheng et al., 2020) and induce transient HRV modification toward sympathetic suppression (Multon et al., 1997; Senat et al., 1998; Subtil et al., 2003; Mulder et al., 2004; Schneider et al., 2010). These modifications seem to last at worst 4 days after the first dose (Mulder et al., 2004; Verdurmen et al., 2013) and the influence on ANS modulation seems minor (Verdurmen et al., 2018; Noben et al., 2019). However, the long-term impact is currently under investigation. There appears to be an association between multiple courses of sGCs prenatal administrations and infant neurodevelopmental abnormalities (Spinillo et al., 2004). Nevertheless, the presence of many confounding factors and the small sample sizes make unclear the mechanism of action of glucocorticoids on fHRV and consequently the impact on ANS development (Verdurmen et al., 2013).

Prenatal exposure to sGC may interfere both with the fetal endogenous cortisol sensitivity, which involves the hippocampus, amygdala, and HPA axis and with the fetal cardiovascular and metabolic regulatory systems (Seckl and Meaney, 2004). This could lead to profound epigenetic changes in the fetal nervous system development and specifically in the vagal functionality, with possible long-term consequences (Crudo et al., 2013; Chang, 2014). This *in utero* programming effect on the fetal HPA axis could last until adulthood: there are recent observations of altered R-R interval variability in adult offspring exposed to elevated fetal sGCs (Sheng et al., 2020), a result that deserves further investigations to better understand the sGCs potential role in neurodevelopmental altered pathways.

###### Drugs for Maternal Pathologies and Drug Abuse.

Most of the medication administered to the mother during pregnancy can cross the placental barrier and reach the fetus.

Selective serotonin reuptake inhibitors (SSRIs) seem to induce alterations in the fetal limbic system (Lattimore et al., 2005), disrupting the central serotonin signaling (Oberlander et al., 2009). Exposure to SSRIs during the 3rd trimester is associated with blunted FMs and reactivity to VAS, as well as with reduced fHRV at 36 WGA (Oberlander et al., 2009; Nguyen et al., 2019). The fetuses, moreover, appear to have an increased risk of preterm birth (Morrison et al., 2005; Suri et al., 2007; Oyebode et al., 2012) and poor neonatal adaptation syndrome (Lattimore et al., 2005; Sivojelezova et al., 2005).

Neonates affected by *in utero* exposure to SSRIs display fewer HRV rhythms and changes in behavioral states, based on the time drugs were administered (Zeskind and Stephens, 2004). They may show altered pain reactivity and parasympathetic cardiac modulation during recovery after an acute noxious event (De las Cuevas and Sanz, 2006), which lasts up to two months, and also altered HPA stress response patterns and hippocampal plasticity (Morrison et al., 2005; Oberlander et al., 2009; Gemmel et al., 2019).

Among antihypertensive drugs, methyldopa does not seem to particularly affect fetal well-being. In contrast, the first-trimester exposure to angiotensin-converting enzyme inhibitors is associated with the greater incidence of malformations of the cardiovascular and CNS (Podymow and August, 2008, 2011), and the timing of exposure seems to be particularly relevant (Caton Alissa et al., 2009). The use of nifedipine concurrently with magnesium sulfate (MgSO4) seems associated with severe hypotension, neuromuscular blockade, and cardiac depression in the fetus (Khedun et al., 2000).

The effects of prenatal opioids exposure on the fetus are widely described. Although there are not evident depressive effects on non-stress test reactivity or variability in the case of daily opioid use for chronic pain (Brar et al., 2020), the current guidelines recommend a prescription at the lowest effective dose (Ray-Griffith et al., 2018).

The opioid prenatal overexposure has instead neurotoxic consequences on several fetal systems: cardiovascular, respiratory, neurobehavioral, metabolic, and neuroendocrine (Conradt et al., 2018). Early exposure of buprenorphine induces higher levels of fHRV, whereas at 32–36 GWA fetuses display less suppression of motor activity (Jansson et al., 2011; Conradt et al., 2018), indicating the involvement of the vagus nerve system. Opioid dependence, moreover, is associated with the increased risk for neonatal opioid withdrawal syndrome (NOWS) (McCarthy et al., 2017; Conradt et al., 2018, 2019).

After birth neonates with NOWS show increased levels of norepinephrine, corticotropin, noradrenaline, and acetylcholine, and lower dopamine and serotonin levels (Conradt et al., 2018), thus suggesting a possible disruption in vagal and CAP activity. Exposure to opioids prenatally could result in programming effects on stress response systems, leading also to long-term consequences (Conradt et al., 2018, 2019).

Opioids are often used as analgesic therapy during labor. There are many observed effects of this medication on fHRV and fetal breathing patterns, mostly transient and dose-/time-dependent (van Geijn et al., 1980; Kariniemi and ÄMmälä, 1981; Low et al., 1981; Viscomi et al., 1990; Capogna, 2001; Mattingly et al., 2003; Shekhawat et al., 2020). To better understand the real implications of intrapartum opioids administration on fetal vagus nerve development, however, it is essential that future research distinguishes its effects from that produced by other substances and associated environmental stressors, illustrating the role of the timing of exposure in specific windows of fetal neurodevelopment, as well as the potential long-term outcomes (Conradt et al., 2018, 2019).

It is also in common use during the peripartum period to administer the mother antibiotic prophylaxis. This seems to be associated with an increased risk for antibiotic-resistant neonatal sepsis (Mercer et al., 1999; Hantoushzadeh et al., 2020). Moreover, prophylactic antibiotic use during vaginal or cesarean delivery may indirectly lead to epigenetic changes in the fetus, with possible implications in the immune system and stress response (Tribe et al., 2018), consequently involving vagus nerve development and suggesting, therefore, the utility to consider potential alternative approaches (Ledger and Blaser, 2013).

###### Drugs Used to Manage the Timing of Delivery.

MgSO4, which is often administered between 23 and 27 WGA or intrapartum for its tocolytic effect to delay labor onset in pregnancy at risk for preterm birth (Ramsey and Rouse, 2001; Ingemarsson and Lamont, 2003), does not seem to show a significant change in fHRV (Stallworth et al., 1981; Nensi et al., 2014). These results do not vary in co-administration with opioids (Cañez et al., 1987), whereas the association with sGC should deserve greater attention by the clinicians (Verdurmen et al., 2017). Moreover, the prolonged administration of MgSO4 seems to be associated with a change in fetal breathing movements (Peaceman et al., 1989) and to affect fHRV dose-dependently (Cardosi and Chez, 1998; Kamitomo et al., 2000).

### The Labor/Delivery Influence on the ANS

Labor is divided into three stages. The 1st stage begins when significant and regular contractions start and ends when there is a full cervical dilation of 10 cm. The 2nd stage of labor begins with complete cervical dilation and ends with the delivery of the fetus. The 3rd stage commences when the fetus is delivered and ends with the delivery of the placenta (afterbirth) (Hutchison et al., 2021).

In this section, we will review the influences that the events that can occur in the first two stages of labor can have on the development of the vagus nerve and ANS. In particular, we will study what events can interfere with the correct development of these two systems by analyzing the evidence from preclinical and clinical studies in this regard, with the aim of preventing potential complications with long-term consequences.

Changes in fHR and fHRV during labor can reveal alterations in fetal reserve to survive the trial of labor and in ANS development, specifically, also in vagus nerve modulation (Chiera et al., 2020). However, it is important to highlight that so far fHR monitoring has not been enough to prevent fetal injury during labor and, consequently, long-term complications. In fact, the role of fHR monitoring (electronic fetal monitoring, EFM, to be precise), despite being used in over 90% of hospitals during delivery, remains controversial (Schifrin, 2020). There is no clarity on its usefulness in decreasing perinatal mortality or cerebral palsy when measured by a CTG which has been explained by the incorrect focus of EFM on prediction of acidemia, a poor correlate to fetal injury, instead of predicting fetal cardiovascular decompensation *per se* as well as the limitations of CTG technologies in capturing the short-term time scale fluctuations of HRV reflecting vagal modulations (Alfirevic et al., 2017; Frasch et al., 2017; Frasch, 2018; Gold and Frasch, 2021). Consequently, EFM fails to identify fetuses at risk of brain injury; this is because there is no clear correlation between fetal brain injury and acidemia (Westerhuis et al., 2007; Cahill et al., 2017). Additional modalities of EFM have been proposed such as intrapartum maternal transabdominal electrocardiography (ECG) and electroencephalography (EEG) which will increase the ability of EFM to predict fetal brain injury. Fetal EEG, while feasible, has not yet made its way into clinical practice. Fetal scalp electrodes can also be used to directly measure pH during childbirth, but this technique, being even more invasive, is not yet fully accepted and also has some complications (Sabir et al., 2010).

#### Preclinical Studies

Through preclinical studies on animals and using fHRV, which remains the best method to monitor the fetus, researchers are trying to find solutions to improve and increase the prevention of specific complications. The preclinical studies analyzed in this review were selected from among those carried out on the ovine species. Sheep are in fact an important model for simulating human pregnancy. Research on the ovine species, reproducing the conditions of human labor, is indeed leading to the discovery and evidence of different algorithms to prevent pathological complications (Frasch et al., 2011; Wang et al., 2014).

In this paragraph, we will consider the studies that simulate the 1st stage of labor.

During this stage, uterine contractions expose the fetus to continuous and significant hypoxic stress. Perinatal hypoxia contributes significantly to the possibility of perinatal brain injury (Gotsch et al., 2007). Statistically, severe fetal acidemia (pH < 7.00) has been shown to occur in between 0.5 and 10% of human births. Of the neonates in this series, 20% will have neurological consequences including cerebral palsy and HIE (Goldaber et al., 1991; DuPont et al., 2013).

Among the possible fHRV metrics, RMSSD is a metric that allows researchers to assess what influence labor may have on the vagus nerve. Indeed, it is known to increase with worsening acidemia (Task Force of the European Society of Cardiology and the North American Society of Pacing and Electrophysiology, 1996; Frasch et al., 2007, 2009a,b; Durosier et al., 2014).

Gold et al. hypothesized, through the use of an algorithm based on RMSSD, the possibility of identifying the precise moment, during labor, when fetuses begin to lose maintenance of cardiac output, that is, when they show a hypotensive pattern of pathological arterial blood pressure (ABP). This hypothesis is highly clinically relevant because, in the case of repetitive umbilical cord occlusions with worsening acidemia, fetuses may easily develop cardiovascular decompensation, a phenomenon that occurs through ABP responses to HR decelerations. Such a failure in maintaining cardiac output during labor could facilitate brain injury in newborns and can be detected on the individual basis using fHRV analysis from EFM (Gold et al., 2021; Roux et al., 2021).

The above-mentioned studies show us what impact labor can have on the ANS and the vagus nerve. In fact, the brain, through the CAN, controls the sympathetic and parasympathetic preganglionic motoneurons and regulates the activity of the vagus nerve (Benarroch, 1993). Consequently, a cerebral alteration, caused by a brain injury, affects the ANS including the vagus. This is the reason why a measure of vagal modulation like RMSSD could help predict a similar event.

Another common complication during labor is given by cardiac repercussions that can affect the fetus. Hypoxia can induce heart failure which is revealed by accelerations and decelerations in fHRV (Rivolta et al., 2014). This event shows how the fetal ANS quickly reacts in the face of a stressor. Further research is required to tie these findings with the recent discovery of the intrinsic fHRV produced by the heart's sino-atrial node (Frasch et al., 2020a). The evidence so far shows that the intrinsic fHRV is influenced by the fetal ANS activity and, conversely, also shapes the ANS activity into the postnatal period, at least in part via the afferent fibers of the vagus nerve (Herry et al., 2021).

Vagal modulation, as revealed by RMSSD, and global ANS functioning, as revealed by entropic HRV metrics, can also be affected by the neuroinflammation *in utero* that can occur during labor due to short-term hypoxic acidemia. A key role in the control of inflammation is played by the CAP and a functional neuroimmunological link in the fetus has been shown to improve postnatal health and brain injury (Frasch et al., 2016; Liu et al., 2016). Cholinergic anti-inflammatory pathway also plays a very important role in preventing and ameliorating NEC cases (Garzoni et al., 2013). During labor, especially in cases of prematurity, there are several conditions that can cause NEC: two of these are certainly hypoxia and acidemia (Sharma and Hudak, 2013). Since the vagus is closely related to the intestinal system, severe intestinal inflammation such as NEC can destroy the cells of the vagal nuclei in the brainstem and inhibit CAP (Fritze et al., 2014).

#### Clinical Studies

Labor in humans is certainly associated with physiological changes, most of which are regulated by ANS (Sanghavi and Rutherford, 2014; Soma-Pillay et al., 2016). In this paragraph, we will focus on clinical studies done during both the 1st stage of labor and the 2nd stage of labor.

##### The 1st Stage of Labor

During this stage, various components come into play that create an alternate activity between SNS and PNS (Norman et al., 2011; Musa et al., 2017). For example, the maternal pain and anxiety due to uterine contractions induce an activation of the sympathetic division (Jones and Greiss, 1982), whereas the release of the hormone oxytocin instead activates the parasympathetic division (Gamer and Büchel, 2012).

A possible critical event during the first stage of labor is certainly the risk of cardiac events. By examining cardiac autonomic modulation (CAM), some authors hypothesized that there might be a predominance of activation of the SNS over the PNS. However, SNS hyperactivity during labor is unlikely to manifest like myocardial infarction, as sympathetic CAM increases simultaneously with HRV metrics related to PNS (Musa et al., 2017).

An important factor that is present in several cases of labor is epidural analgesia (Bautista and George, 2020). Its influence on the development of the ANS and the vagus nerve can be understood through a careful examination of fHRV changes. In fact, epidural analgesia itself does not show precise or significant changes in fHRV (Lavin, 1982), but it can interact with other aspects already existing in the mother. Hypotension and uterine hypertonia, for instance, can create significant imbalances in the fHRV during epidural analgesia in labor (Lavin, 1982). Several studies have also shown a major change in fHRV in women given an analgesic agent such as lidocaine (Boehm et al., 1975; Lavin, 1982).

An aspect that should not be underestimated in the administration of epidural analgesia is the position of the mother. fHRV undergoes fewer changes with the use of the full lateral position, compared with the use of wedged supine position (Preston et al., 1993). The reason behind this evidence could be the greater prevention of aortocaval compression given by the full lateral position (Preston et al., 1993). The 1st stage of labor does not always occur naturally and without complications: consequently, there may be an artificial intervention that causes the induction of labor to increase uterine contractions. Labor is usually induced through the administration of synthetic oxytocin (sOT). As previously mentioned, oxytocin plays an important role in activating the PNS and it has been shown to dampen SNS (Gamer and Büchel, 2012). However, the induction of oxytocin did not reveal cardiac complications in women with heart disease (Dogra et al., 2019).

##### The 2nd Stage of Labor

Although the induction of the hormone oxytocin has no cardiac repercussions, it can still influence the development of the vagus nerve and the ANS in the last hours of labor. It has been shown that a pattern of hyperactive uterine contractions (often caused by the administration of sOT), during the last 2 h of labor, is strongly associated with acidemia and hypoxia at birth and with fHR and fHRV alterations (Freidman and Sachtleben, 1978; Woodson et al., 1979; Cibils and Votta, 1993; Ladfors et al., 2002; Jonsson et al., 2008; Verspyck and Sentilhes, 2008; Aye et al., 2014). As previously mentioned, acidemia can induce NEC and consequently inhibit the CAP (Garzoni et al., 2013).

The long-term consequences of developing cardiac control systems and ANS are hence under investigation (Frasch and Giussani, 2020). Moreover, sOT infusion may decrease spontaneous oxytocin release (Jonas et al., 2009), elevating days after birth fetal plasmatic oxytocin concentration, not dampening the HPA axis response, and increasing the CAN system control activity (Yee et al., 2016).

Exposure to sOT may therefore lead to epigenetic remodeling in the infant (Tribe et al., 2018; Uvnäs-Moberg et al., 2019), and the documented presence in animal models of specific binding for oxytocin in the vagal dorsal motor nucleus during early embryonic life (Tribollet et al., 1989) suggests a strict link between oxytocin and vagus nerve.

Drugs aside, the most relevant aspect to be evaluated during the 2nd stage of labor is certainly the mode of delivery.

The major critical events that can affect vagus nerve and ANS development occur in premature births and delivery by CS at term (Hillman et al., 2012; Tribe et al., 2018; Mulkey et al., 2019).

The case of premature births is probably the most challenging. Preterm newborns have an immature ANS, which has great repercussions on cardiac and respiratory functions (Mulkey and du Plessis, 2018). In infants born before 36 WGA, there is also a 90% incidence of NEC, as the premature gastrointestinal tract has increased permeability (Garzoni et al., 2013).

Although CS delivery is far faster than a vaginal birth modality, the fetus is still subjected to significant stress that can create changes in the development of some systems, including the ANS (Tribe et al., 2018). An important difference should be moreover emphasized between the CS delivery following a period of labor and CS delivery prior to the onset of labor. Infants born with CS delivery following a period of labor have a higher and more developed ANS tone compared to those born with CS without labor (Mulkey et al., 2019). Furthermore, labor pain before CS helps prevent respiratory disorders and avoid umbilical cord acidemia, thus protecting the developing ANS (Senturk et al., 2015).

Delivery without labor or preterm birth is associated with lower cortisol, angiotensin, and catecholamines levels compared to labored or full-term delivery. This situation puts newborns at risk of cardiovascular and metabolic dysfunctions, which can then negatively affect the developing brain by inducing inflammation or glucose deficiency (Hillman et al., 2012; Morton and Brodsky, 2016; Mulkey and du Plessis, 2018).

### The Incubator Influence on the ANS

Once born, newborns have to deal with the extrauterine environment that, especially for preterm babies, is constituted by the incubator. Here, environmental factors such as temperature, light, noise, procedures could induce a high amount of stress, which could become toxic, that is, induce severe alterations in the brain and organic development, with long-lasting complications (De Jonckheere and Storme, 2019; Weber and Harrison, 2019).

#### Temperature

As thermoregulation is one of the most important ANS functions, the temperature can easily affect ANS development. Indeed, warm incubators tend to increase SNS tone and reduce PNS activity, whereas the opposite is true for incubators 2°C colder than newborns' temperature (Franco et al., 2003; Stéphan-Blanchard et al., 2013). Therefore, temperature that constantly deviates from newborns' one can lead to excessive ANS activation. Indeed, extreme variations of environmental or core temperature can impair the ANS, with all due complications (Fox and Matthews, 1989; Mowery et al., 2011).

Nonetheless, when used correctly, the temperature can also help newborns to correctly develop: indeed, therapeutic hypothermia in newborns with 36 WGA in case of HIE can help reduce brain and injury brain inflammation, thus having positive effects on neurodevelopment (Massaro et al., 2017; Lemyre and Chau, 2018).

#### Noise

If born prematurely, newborns lose the low-frequency maternal sounds that are essential for the correct maturation of their hearing system, with negative consequences on brain maturation and speech development (McMahon et al., 2012).

Furthermore, without uterus protection, newborns can be particularly sensitive to sounds, which usually overcome the 45 dB limit recommended by the American Academy of Pediatrics (Almadhoob and Ohlsson, 2020), thus resulting in toxic stress (Weber and Harrison, 2019). The consequences can be quite severe: from apnea to hypoxemia, from sleep disruption to decreased growth, from immunity impairment to neuroendocrine alterations (Almadhoob and Ohlsson, 2020).

Therefore, every possible procedure aimed to protect babies from harmful noises or to introduce positive sounds including the human voice, especially maternal voice (even recorded), and songs, can result in important neuroprotective effects (McMahon et al., 2012; Weber and Harrison, 2019). It is noteworthy that earmuffs show ambiguous results, that is, they can both help oxygenation and sleep and provoke stress (Zahr and de Traversay, 1995; Aita et al., 2013; Almadhoob and Ohlsson, 2020).

#### Light

As newborns pass the majority of their time sleeping, excessive light due to incubators not properly covered can negatively affect the newborns' cardiorespiratory regulation, thus increase stress level and altering ANS development (Ozawa et al., 2010; Weber and Harrison, 2019), in addition, to induce all the negative effects of sleep deficiency. Moreover, excessive light can impair visual development, thus increasing SNS and HPA axis activation (Weber and Harrison, 2019). On the other side, properly covering incubators or using eye masks facilitate a quiet sleep state characterized by a stable respiratory function (Shiroiwa et al., 1986; Venkataraman et al., 2018).

#### Electromagnetic Fields

Newborns can also be subject to EMFs, from the incubator or the care unit environment. Electromagnetic field coming from the incubator power influences the ANS as revealed by HRV analysis as LF/HF increases, whereas HF decreases (Bellieni et al., 2008). The farther away newborns stay from the incubator power, the less the ANS is affected (Bellieni et al., 2008; Passi et al., 2017).

Although incubator EMF seems to be under the normative value, EMFs could become dangerous when the incubator, and thus the newborn, is surrounded by other instruments or staff's devices that, through their own EMF, can give rise to electromagnetic interference (Besset et al., 2020). This phenomenon has the potential of altering ANS in three ways, especially in preterm or vulnerable infants. Firstly, by directly affecting brain development, as the skull has a low bone density that fails in adequately protecting newborns' brain from EMF (Besset et al., 2020). Secondly, by suppressing melatonin secretion, thus altering the immune system and, potentially, the CAP, with all the negative consequences already mentioned. Lastly, instrumental malfunctioning can induce life-threatening conditions (Carvajal de la Osa et al., 2020).

#### Incorrect Oxygenation

Correct oxygenation is paramount for the newborn's development and survival (Ho et al., 2020). Indeed, in preterm infants, both too high or too low oxygenation can be dangerous for the nervous system. For instance, too high oxygenation can easily induce lung injury, thus increasing inflammation and stressing the already immature CAP, whereas too low oxygenation can cause neurodevelopmental impairment and HIE (Rantakari et al., 2021).

It is noteworthy that delayed umbilical cord clamping, that is, delaying the procedure by 60–180 s seems to improve both peripheral and cerebral oxygenation, in addition to several hemodynamics parameters, which could reveal a positive effect on ANS function and development (Bruckner et al., 2021).

#### Posture

Babies' position is another factor that could impact ANS.

Indeed, a prone position improves oxygenation, increases PNS tone, and lowers both salivary cortisol and stress behavior (Gomes et al., 2019). Nevertheless, prone sleeping is considered a risk factor for SIDS and it shows a lower short-term variability in HRV (Lucchini et al., 2016). It is thus noteworthy that, in the case of prone sleeping position, between the 2nd and 4th months after birth, both blood pressure and cerebral oxygenation seem to decrease, whereas there is a propensity for cortical arousal, maybe to counter the fall in pressure and oxygenation. This would explain why ANS impairment, for instance, due to prematurity, can make newborns more vulnerable to SIDS (Horne, 2018). As supporting evidence, complications including fetal growth restriction alter sleep stages by reducing active sleep (Aldrete-Cortez et al., 2019).

Supine position with manual restraint for flexion seems to have positive effects on ANS as measured through HRV (Gomes et al., 2019). In the same way, side position during feeding seems to have stabilizing effects on both ANS, as revealed by HR, and oxygenation in preterm newborns (Thoyre et al., 2012), although results are conflicting (Raczyńska and Gulczyńska, 2019).

#### Respect for the Baby's Sleep

Sleep impairment due to prematurity or environmental factors can lead to global neurodevelopmental complications (e.g., attention deficit, motor disability, and low cognitive abilities), as newborns show weaker functional connectivity networks during such impaired sleep (Uchitel et al., 2021).

Drugs including theophylline, caffeine, and methylxanthine, used to support respiratory functions have the potential of altering sleep quality and induce drowsiness. In the same way, improper light and noise can disrupt newborns' sleep duration, especially in preterm and very preterm ones (Gogou et al., 2019). But maybe, more than anything, medical procedures tend to impair babies' sleep the most, in particular since in preterm newborns many interventions are perceived as noxious and painful, thus heightening the stress response (Holsti et al., 2006; Weber and Harrison, 2019). Luckily, applying gentle touch, an “intervention” that stimulates the vagus nerve via C-tactile fibers, to medical care has the potential to regulate ANS function (Manzotti et al., 2019) and, thus, it is conceivable it could have protective effects on sleep. Otherwise, even clustered-care could help reduce the time the babies' sleep is disrupted (Holsti et al., 2006).

## Clinical Implications for the Management of Fetuses and Newborns

“*The newborn is not new, and neither is the fetus”*.

*Paraphrased from a personal communication with Dr. Barry Schifrin*

Our current clinical management of fetal and maternal health is not individualized and intermittent, suffering from a pattern of catching up with clinical symptoms instead of using biometric data to predict and steer developmental trajectories and health outcomes for the pregnant mother and her fetus during the delivery and after to their fullest potential. The fear of missing fetal injury during labor has led to soaring CS rates and the failure of the EFM has resulted in the misguided dissent over its utility in general throwing the proverbial baby with the bathwater.

What is amiss is the understanding and acting upon the fact that each fetus and newborn are not truly new, but, rather, come with weeks and months of experience *in utero*. Can we learn to assess this individual experience?

In this review, we highlighted the growing understanding of the vagus nerve's developmental physiology, the largest and perhaps most important homeostatic (or, better, homeokinetic) regulatory system in our body (Macklem and Seely, 2010; Herry et al., 2019). The exciting take-home message is that much of this development can be gauged non-invasively during pregnancy using readily available bioelectric sensors such as ECG or, less qualitatively well, but still useful, using ultrasound-derived HR estimation, the mainstay of today's CTG and EFM technologies that is in sore need of technological disruption.

What could and should we do to disrupt this status quo and enable individualized insights into the various developmental effects the vagus nerve maturation and pleiotropic physiology provide?

Research is increasingly pointing toward the existence of an “HRV code,” that is, specific HRV spatio-temporal alterations related to specific organic alterations (e.g., hypoxia, cerebral, cardiac, or gut pathologies) (Herry et al., 2019; Frasch, 2020), to the impact of external factors (e.g., smoking, physical activity, diabetes), and to the fetal brain and nervous development (Hoyer et al., 2012, 2013a, 2015, 2017, 2019). Indeed, fHRV can be affected by both neural factors (i.e., ANS) and non-neural factors, e.g., metabolic and organic functions (DiPietro et al., 2015).

Therefore, knowing that the ANS-organ connections are established so early in the embryo could offer useful insight for comprehending the complex role of the vagus nerve and autonomic ganglia in the developing embryo/fetus. Currently, ANS development is usually assessed during the 3rd trimester, when vagus nerve myelination begins (Mulkey and du Plessis, 2018). However, the literature shows that the ANS can be assessed through fHRV from 15 to 20 WGA (Hoyer et al., 2012, 2013b, 2015, 2019; Shuffrey et al., 2019). Moreover, the heartbeat can be heard through ultrasound as early as the 6th WGA, the HR accelerates constantly during the first 8 WGA (Rodgers et al., 2015) and it was recently discovered that an intrinsic fHRV (i.e., due to sino-atrial node rhythms) exists—it can affect the global fHRV and be influenced by pregnancy events (e.g., hypoxia) (Frasch et al., 2020a) and it shows a specific “signature,” that is, specific HRV metrics (Herry et al., 2019). Maternal and transabdominal ECG recorded in the early third trimester carry information about the chronic exposure of the mother–fetus dyad to stress detectable by deep learning, a form of artificial intelligence techniques (Sarkar et al., 2021). Therefore, it would be worth it to improve fetal cardiac monitoring technology in order to detect ECG and fHRV even in the first weeks of pregnancy.

Such an advancement could improve the embryo's health assessment during early pregnancy, thus going well-beyond detecting embryonic–fetal malformations and toward individualized prospective functional assessments. For instance, although we know that folate deficiency can impede the neural tube closure or that alcohol can induce dramatic brain modifications (Moore et al., 2016), we still lack knowledge about more subtle influences, that is, the precise effects of just some alcohol during the first weeks of pregnancy or the early effects of maternal stress (Avalos et al., 2014; Caspers Conway et al., 2014; Antonelli et al., 2021). On the other hand, we are learning that certain factors (e.g., choline supplementation) may protect the fetus from excessive HPA axis activation and have long-lasting (into adulthood) effects on cognition through neural and epigenetic modifications (Korsmo et al., 2019). Furthermore, some studies have already shown the feasibility of correlating external factors related to maternal lifestyle or “internal” factors such as FM to fHRV changes as early as 20–28 WGA (DiPietro et al., 2007; Hoyer et al., 2019; Shuffrey et al., 2019).

As these findings are available for the 2nd and 3rd trimester, assessing fHRV in the 1st trimester may shed more light on what can protect or damage the ANS in these early formative stages of development and the consequences of its alterations.

Taken together, non-invasive assessment of maternal and fetal health via HRV monitoring throughout the pregnancy, from its earliest stages, will enable an individualized risk profiling and outcome projection during the latter stages of gestation and during delivery, a true disruption of the status quo that will improve maternal and perinatal health outcomes.

## Data Availability Statement

The original contributions presented in the study are included in the article/supplementary material, further inquiries can be directed to the corresponding authors.

## Author Contributions

FC, MC, SV, CV, and AM conceptualized the paper. FC, MC, SV, CV, MA, and MF wrote the first draft. All authors reviewed, edited and approved the final version of the paper.

## Conflict of Interest

MF has patents on aECG (WO2018160890A1) and EEG technologies for fetal monitoring (US9215999). The remaining authors declare that the research was conducted in the absence of any commercial or financial relationships that could be construed as a potential conflict of interest.

## Publisher's Note

All claims expressed in this article are solely those of the authors and do not necessarily represent those of their affiliated organizations, or those of the publisher, the editors and the reviewers. Any product that may be evaluated in this article, or claim that may be made by its manufacturer, is not guaranteed or endorsed by the publisher.

## Abbreviations

ABP: arterial blood pressure
ANS: autonomic nervous system
ASD: autism spectrum disorders
CAM: cardiac autonomic modulation
CAN: central autonomic network
CAP: cholinergic anti-inflammatory pathway
CNS: central nervous system
CRH: corticotropin-releasing hormone
CS: cesarean section
CTG: cardiotocography
ECG: electrocardiography
EEG: electroencephalography
EFM: electronic fetal monitoring
EMF: electromagnetic field
ENS: enteric nervous system
FASD: fetal alcohol spectrum disorders
fHR: fetal heart rate
fHRV: fetal heart rate variability
FM: fetal movement
GM: general movement
HIE: hypoxic-ischemic encephalopathy
HPA: hypothalamic-pituitary-adrenal
HR: heart rate
HRV: heart rate variability
NAS: neonatal abstinence syndrome
NC: neural crest
NEC: necrotizing enterocolitis
NOWS: neonatal opioid withdrawal syndrome
PAE: prenatal alcohol exposure
PGP: pelvic girdle pain
PNE: prenatal nicotine exposure
PNS: parasympathetic nervous system
REM: rapid eye movements
SDB: sleep-disordered breathing
sGC: synthetic glucocorticoid
SIDS: sudden infant death syndrome
SNS: sympathetic nervous system
sOT: synthetic oxytocin
SSRI: selective serotonin reuptake inhibitor
VAS: vibroacoustic stimulation
WGA: week gestational age
ZIKV: Zika virus.

## References

[B1] Aagaard K. Ma J. Antony K. M. Ganu R. Petrosino J. Versalovic J. (2014). The placenta harbors a unique microbiome. Sci. Transl. Med. 6:237ra65. 10.1126/scitranslmed.3008599 24848255 PMC4929217

[B2] Abdel-Haq R. Schlachetzki J. C. M. Glass C. K. Mazmanian S. K. (2018). Microbiome–microglia connections via the gut–brain axis. J. Exp. Med. 216, 41–59. 10.1084/jem.20180794 30385457 PMC6314531

[B3] Abrams R. M. Gerhardt K. J. (2000). The acoustic environment and physiological responses of the fetus. J. Perinatol. 20, S31–S36. 10.1038/sj.jp.7200445 11190698

[B4] Aidy S. E. Dinan T. G. Cryan J. F. (2015). Gut microbiota: the conductor in the orchestra of immune–neuroendocrine communication. Clin. Ther. 37, 954–967. 10.1016/j.clinthera.2015.03.002 25846319

[B5] Aita M. Johnston C. Goulet C. Oberlander T. F. Snider L. (2013). Intervention minimizing preterm infants' exposure to NICU light and noise. Clin. Nurs. Res. 22, 337–358. 10.1177/1054773812469223 23275433

[B6] Al Alam D. Danopoulos S. Grubbs B. Ali N. A. B. M. MacAogain M. Chotirmall S. H.. (2020). Human fetal lungs harbor a microbiome signature. Am. J. Respir. Crit. Care Med. 201, 1002–1006. 10.1164/rccm.201911-2127LE 31898918 PMC7159424

[B7] Aldrete-Cortez V. Poblano A. Tafoya S. A. Ramírez-García L. A. Casasola C. (2019). Fetal growth restriction: from polyvagal theory to developmental impairments? Brain Dev. 41, 769–775. 10.1016/j.braindev.2019.04.014 31056231

[B8] Alfirevic Z. Devane D. Gyte G. M. Cuthbert A. (2017). Continuous cardiotocography (CTG) as a form of electronic fetal monitoring (EFM) for fetal assessment during labour. Cochrane Database Syst. Rev. 2:CD006066. 10.1002/14651858.CD006066.pub3 16856111

[B9] Allen L. H. (2001). Biological mechanisms that might underlie iron's effects on fetal growth and preterm birth. J. Nutr. 131, 581S−589S. 10.1093/jn/131.2.581S 11160591

[B10] Almadhoob A. Ohlsson A. (2020). Sound reduction management in the neonatal intensive care unit for preterm or very low birth weight infants. Cochrane Database Syst. Rev. 1:CD010333. 10.1002/14651858.CD010333.pub3 31986231 PMC6989790

[B11] AlSaif S. Mumtaz S. Wray S. (2015). A short review of adipokines, smooth muscle and uterine contractility. Life Sci. 125, 2–8. 10.1016/j.lfs.2015.02.001 25711427

[B12] Al-Shargabi T. Govindan R. B. Dave R. Metzler M. Wang Y. du Plessis A.. (2017). Inflammatory cytokine response and reduced heart rate variability in newborns with hypoxic-ischemic encephalopathy. J. Perinatol. 37, 668–672. 10.1038/jp.2017.15 28252659 PMC5446303

[B13] Amarasekara R. Jayasekara R. W. Senanayake H. Dissanayake V. H. W. (2015). Microbiome of the placenta in pre-eclampsia supports the role of bacteria in the multifactorial cause of pre-eclampsia. J. Obstet. Gynaecol. Res. 41, 662–669. 10.1111/jog.12619 25492799

[B14] Antonelli M. C. Frasch M. G. Rumi M. Sharma R. Zimmermann P. Molinet M. S.. (2021). Early biomarkers and intervention programs for the infant exposed to prenatal stress. Curr. Neuropharmacol. 10.2174/1570159X19666210125150955. [Epub ahead of print]. 33550974 PMC9199558

[B15] Antony K. M. Ma J. Mitchell K. B. Racusin D. A. Versalovic J. Aagaard K. (2015). The preterm placental microbiome varies in association with excess maternal gestational weight gain. Am. J. Obstet. Gynecol. 212, 653.e1–653.e16. 10.1016/j.ajog.2014.12.041 25557210 PMC4892181

[B16] Aplin J. D. Myers J. E. Timms K. Westwood M. (2020). Tracking placental development in health and disease. Nat. Rev. Endocrinol. 16, 479–494. 10.1038/s41574-020-0372-6 32601352

[B17] Avalos L. A. Roberts S. C. Kaskutas L. A. Block G. Li D.-K. (2014). Volume and type of alcohol during early pregnancy and the risk of miscarriage. Subst. Use Misuse 49, 1437–1445. 10.3109/10826084.2014.912228 24810392 PMC4183196

[B18] Aye C. Y. L. Redman C. W. G. Georgieva A. (2014). The effect of augmentation of labour with syntocinon on the fetal CTG using objective computerised analysis: a nested case-control study. Eur. J. Obstet. Gynecol. Reprod. Biol. 176, 112–118. 10.1016/j.ejogrb.2014.02.032 24655553

[B19] Baker T. G. Scrimgeour J. B. (1980). Development of the gonad in normal and anencephalic human fetuses. Reproduction 60, 193–199. 10.1530/jrf.0.0600193 7431319

[B20] Bale T. L. (2016). The placenta and neurodevelopment: sex differences in prenatal vulnerability. Dialogues Clin. Neurosci. 18, 459–464. 10.31887/DCNS.2016.18.4/tbale 28179817 PMC5286731

[B21] Barlow-Anacker A. J. Fu M. Erickson C. S. Bertocchini F. Gosain A. (2017). Neural crest cells contribute an astrocyte-like glial population to the spleen. Sci. Rep. 7:45645. 10.1038/srep45645 28349968 PMC5368681

[B22] Bassols J. Serino M. Carreras-Badosa G. Burcelin R. Blasco-Baque V. Lopez-Bermejo A.. (2016). Gestational diabetes is associated with changes in placental microbiota and microbiome. Pediatr. Res. 80, 777–784. 10.1038/pr.2016.155 27490741

[B23] Bautista L. George R. B. (2020). Epidural analgesia in labour. CMAJ 192:E509. 10.1503/cmaj.191372 32575042 PMC7234272

[B24] Beauchaine T. P. Gatzke-Kopp L. Mead H. K. (2007). Polyvagal theory and developmental psychopathology: emotion dysregulation and conduct problems from preschool to adolescence. Biol. Psychol. 74, 174–184. 10.1016/j.biopsycho.2005.08.008 17045726 PMC1801075

[B25] Bekedam D. J. Mulder E. J. H. Snijders R. J. M. Visser G. H. A. (1991). The effects of maternal hyperoxia on fetal breathing movements, body movements and heart rate variation in growth retarded fetuses. Early Hum. Dev. 27, 223–232. 10.1016/0378-3782(91)90196-A 1802673

[B26] Bellieni C. V. Acampa M. Maffei M. Maffei S. Perrone S. Pinto I.. (2008). Electromagnetic fields produced by incubators influence heart rate variability in newborns. Arch. Dis. Child. Fetal Neonatal Ed. 93, F298–F301. 10.1136/adc.2007.132738 18450804

[B27] Benarroch E. E. (1993). The central autonomic network: functional organization, dysfunction, and perspective. Mayo Clin. Proc. 68, 988–1001. 10.1016/s0025-6196(12)62272-1 8412366

[B28] Benny P. Al-Akwaa F. M. Dirkx C. Schlueter R. J. Wolfgruber T. K. Chern I. Y.. (2019). Placenta Microbiome Diversity Is Associated with Maternal Pre-Pregnancy Obesity and Placenta Biogeography. Rochester, NY: Social Science Research Network. 10.2139/ssrn.3441806

[B29] Bernardes J. Gonçalves H. Ayres-de-Campos D. Rocha A. P. (2008). Linear and complex heart rate dynamics vary with sex in relation to fetal behavioural states. Early Hum. Dev. 84, 433–439. 10.1016/j.earlhumdev.2007.12.001 18248921

[B30] Berntson G. G. Cacioppo J. T. (2004). Heart rate variability: stress and psychiatric conditions, in Dynamic Electrocardiography, eds Malik M. Camm A. J. (Oxford, UK: Blackwell Publishing), 57–64. 10.1002/9780470987483.ch7

[B31] Besset D. Selmaoui B. Tourneux P. Leke A. Delanaud S. de Seze R.. (2020). Environmental radiofrequency electromagnetic field levels in a department of pediatrics. Environ. Res. 181:108894. 10.1016/j.envres.2019.108894 31740038

[B32] Bhide A. Acharya G. (2018). Sex differences in fetal heart rate and variability assessed by antenatal computerized cardiotocography. Acta Obstet. Gynecol. Scand. 97, 1486–1490. 10.1111/aogs.13437 30091138

[B33] Boehm F. H. Woodruff L. F. Growdon J. H. (1975). The effect of lumbar epidural anesthesia on fetal heart rate baseline variability. Anesth. Analg. 54, 779–782. 10.1213/00000539-197511000-00027 1239217

[B34] Bonaz B. Sinniger V. Pellissier S. (2021). Therapeutic potential of vagus nerve stimulation for inflammatory bowel diseases. Front. Neurosci. 15:650971. 10.3389/fnins.2021.650971 33828455 PMC8019822

[B35] Bordeleau M. Fernández de Cossío L. Chakravarty M. M. Tremblay M.-È. (2021). From maternal diet to neurodevelopmental disorders: a story of neuroinflammation. Front. Cell. Neurosci. 14:612705. 10.3389/fncel.2020.612705 33536875 PMC7849357

[B36] Bornstein M. H. DiPietro J. A. Hahn C.-S. Painter K. Haynes O. M. Costigan K. A. (2002). Prenatal cardiac function and postnatal cognitive development: an exploratory study. Infancy 3, 475–494. 10.1207/S15327078IN0304_04

[B37] Borovikova L. V. Ivanova S. Zhang M. Yang H. Botchkina G. I. Watkins L. R.. (2000). Vagus nerve stimulation attenuates the systemic inflammatory response to endotoxin. Nature 405, 458–462. 10.1038/35013070 10839541

[B38] Borre Y. E. O'Keeffe G. W. Clarke G. Stanton C. Dinan T. G. Cryan J. F. (2014). Microbiota and neurodevelopmental windows: implications for brain disorders. Trends Mol. Med. 20, 509–518. 10.1016/j.molmed.2014.05.002 24956966

[B39] Borsani E. Della Vedova A. M. Rezzani R. Rodella L. F. Cristini C. (2019). Correlation between human nervous system development and acquisition of fetal skills: an overview. Brain Dev. 41, 225–233. 10.1016/j.braindev.2018.10.009 30389271

[B40] Brändle J. Preissl H. Draganova R. Ortiz E. Kagan K. O. Abele H.. (2015). Heart rate variability parameters and fetal movement complement fetal behavioral states detection via magnetography to monitor neurovegetative development. Front. Hum. Neurosci. 9:147. 10.3389/fnhum.2015.00147 25904855 PMC4388008

[B41] Brar B. Jackson D. Nat M. Patil P. Iriye B. Planinic P. (2020). Antenatal interventions based upon fetal surveillance of the daily opioid-exposed fetus: a descriptive analysis. J. Matern. Fetal Neonatal Med. 33, 909–912. 10.1080/14767058.2018.1509067 30078349 PMC12134651

[B42] Brown A. Johnston R. (2013). Maternal experience of musculoskeletal pain during pregnancy and birth outcomes: significance of lower back and pelvic pain. Midwifery 29, 1346–1351. 10.1016/j.midw.2013.01.002 23452662

[B43] Brown C. A. Lee C. T. Hains S. M. J. Kisilevsky B. S. (2008). Maternal heart rate variability and fetal behavior in hypertensive and normotensive pregnancies. Biol. Res. Nurs. 10, 134–144. 10.1177/1099800408322942 18829597

[B44] Bruckner M. Katheria A. C. Schmölzer G. M. (2021). Delayed cord clamping in healthy term infants: more harm or good? Semin. Fetal. Neonatal Med. 26:101221. 10.1016/j.siny.2021.101221 33653601

[B45] Bryant J. Jamil R. T. Thistle J. (2020). Fetal movement, in StatPearls (Treasure Island, FL: StatPearls Publishing). Available online at: http://www.ncbi.nlm.nih.gov/books/NBK470566/ (accessed February 22, 2021).

[B46] Burgio E. (2015). Environment and fetal programming: the origins of some current “pandemics.” J. Pediatr. Neonatal Individ. Med. 4:e040237. 10.7363/040237

[B47] Busnel M. Granier-Deferre C. Lecanuet J. (1992). Fetal audition. Ann. N. Y. Acad. Sci. 662, 118–134. 10.1111/j.1749-6632.1992.tb22857.x 1456635

[B48] Buss C. Davis E. P. Class Q. A. Gierczak M. Pattillo C. Glynn L. M.. (2009). Maturation of the human fetal startle response: evidence for sex-specific maturation of the human fetus. Early Hum. Dev. 85, 633–638. 10.1016/j.earlhumdev.2009.08.001 19726143 PMC2767415

[B49] Buyon J. P. (1998). The effects of pregnancy on autoimmune diseases. J. Leukoc. Biol. 63, 281–287. 10.1002/jlb.63.3.281 9500514

[B50] Bystrova K. (2009). Novel mechanism of human fetal growth regulation: a potential role of lanugo, vernix caseosa and a second tactile system of unmyelinated low-threshold C-afferents. Med. Hypotheses 72, 143–146. 10.1016/j.mehy.2008.09.033 19004563

[B51] Cahill A. G. Mathur A. M. Smyser C. D. Mckinstry R. C. Roehl K. A. López J. D.. (2017). Neurologic injury in acidemic term infants. Am. J. Perinatol. 34, 668–675. 10.1055/s-0036-1597135 27926973 PMC6283275

[B52] Cañez M. S. Reed K. L. Shenker L. (1987). Effect of maternal magnesium sulfate treatment on fetal heart rate variability. Am. J. Perinatol. 4, 167–170. 10.1055/s-2007-999765 3566885

[B53] Cao B. Stout M. J. Lee I. Mysorekar I. U. (2014). Placental microbiome and its role in preterm birth. NeoReviews 15, e537–e545. 10.1542/neo.15-12-e537 25635174 PMC4307601

[B54] Capogna G. (2001). Effect of epidural analgesia on the fetal heart rate. Eur. J. Obstet. Gynecol. Reprod. Biol. 98, 160–164. 10.1016/S0301-2115(01)00363-3 11574125

[B55] Caputo C. Wood E. Jabbour L. (2016). Impact of fetal alcohol exposure on body systems: a systematic review. Birth Defects Res. C Embryo Today Rev. 108, 174–180. 10.1002/bdrc.21129 27297122

[B56] Cardosi R. J. Chez R. A. (1998). Magnesium sulfate, maternal hypothermia, and fetal bradycardia with loss of heart rate variability. Obstet. Gynecol. 92, 691–693. 10.1016/S0029-7844(98)00212-9 9764667

[B57] Carvajal de la Osa J. Santana González J. Herrera Galán M. Sánchez Grau A. Pérez Almirall I. (2020). Environmental magnetic field in a neonatal intensive care unit. A relevant verification. Arch. Argent. Pediatr. 118, e246–e251. 10.5546/aap.2020.eng.e246 32470260

[B58] Casagrande D. Gugala Z. Clark S. M. Lindsey R. W. (2015). Low back pain and pelvic girdle pain in pregnancy. J. Am. Acad. Orthop. Surg. 23, 539–549. 10.5435/JAAOS-D-14-00248 26271756

[B59] Caspers Conway K. M. Romitti P. A. Holmes L. Olney R. S. Richardson S. D. the National Birth Defects Prevention Study (2014). Maternal periconceptional alcohol consumption and congenital limb deficiencies: alcohol consumption and limb deficiencies. Birt. Defects Res. A Clin. Mol. Teratol. 100, 863–876. 10.1002/bdra.23292 25132072 PMC4427046

[B60] Castel A. Burns P. M. Benito J. Liu H. L. Kuthiala S. Durosier L. D.. (2020). Recording and manipulation of vagus nerve electrical activity in chronically instrumented unanesthetized near term fetal sheep. *ArXiv200808625 Q-Bio*. Available online at: http://arxiv.org/abs/2008.08625 (accessed May 13, 2021). 10.1016/j.jneumeth.2021.109257 34139266

[B61] Caton Alissa R. Bell Erin M. Druschel Charlotte M. Werler Martha M. Lin Angela E. Browne Marilyn L.. (2009). Antihypertensive medication use during pregnancy and the risk of cardiovascular malformations. Hypertension 54, 63–70. 10.1161/HYPERTENSIONAHA.109.129098 19433779 PMC4913772

[B62] Cerritelli F. Cardone D. Pirino A. Merla A. Scoppa F. (2020). Does osteopathic manipulative treatment induce autonomic changes in healthy participants? A thermal imaging study. Front. Neurosci. 14:887. 10.3389/fnins.2020.00887 33013294 PMC7461826

[B63] Chang J. J. Pien G. W. Duntley S. P. Macones G. A. (2010). Sleep deprivation during pregnancy and maternal and fetal outcomes: is there a relationship? Sleep Med. Rev. 14, 107–114. 10.1016/j.smrv.2009.05.001 19625199 PMC2824023

[B64] Chang Y. P. (2014). Evidence for adverse effect of perinatal glucocorticoid use on the developing brain. Korean J. Pediatr. 57:101. 10.3345/kjp.2014.57.3.101 24778691 PMC4000755

[B65] Chen J. S. Kandle P. F. Murray I. Fitzgerald L. A. Sehdev J. S. (2021). Physiology, pain, in StatPearls (Treasure Island, FL: StatPearls Publishing). Available online at: http://www.ncbi.nlm.nih.gov/books/NBK539789/ (accessed April 23, 2021).

[B66] Chiera M. Cerritelli F. Casini A. Barsotti N. Boschiero D. Cavigioli F.. (2020). Heart rate variability in the perinatal period: a critical and conceptual review. Front. Neurosci. 14:561186. 10.3389/fnins.2020.561186 33071738 PMC7544983

[B67] Christian P. Stewart C. P. (2010). Maternal micronutrient deficiency, fetal development, and the risk of chronic disease. J. Nutr. 140, 437–445. 10.3945/jn.109.116327 20071652

[B68] Christifano D. N. Taylor M. K. Carlson S. E. Colombo J. Gustafson K. M. (2020). Higher maternal weight is related to poorer fetal autonomic function. J. Dev. Orig. Health Dis. 12, 354–356. 10.1017/S2040174420000653 32662379 PMC13250823

[B69] Chung W. C. J. Auger A. P. (2013). Gender differences in neurodevelopment and epigenetics. Pflüg. Arch. Eur. J. Physiol. 465, 573–584. 10.1007/s00424-013-1258-4 23503727 PMC3654067

[B70] Cibils L. A. Votta R. (1993). Clinical significance of fetal heart rate patterns during labor. IX: prolonged pregnancy. J. Perinat. Med. 21, 107–116. 10.1515/jpme.1993.21.2.107 8515353

[B71] Clarkson C. E. Adams N. (2018). A qualitative exploration of the views and experiences of women with pregnancy related pelvic girdle pain. Physiotherapy 104, 338–346. 10.1016/j.physio.2018.05.001 30032932

[B72] Cogan R. Spinnato J. A. (1986). Pain and discomfort thresholds in late pregnancy. Pain 27, 63–68. 10.1016/0304-3959(86)90223-X 3785964

[B73] Committee on Fetus and Newborn and Section on Anesthesiology and Pain Medicine (2016). Prevention and management of procedural pain in the neonate: an update. Pediatrics 137:e20154271. 10.1542/peds.2015-4271 26810788

[B74] Conradt E. Crowell S. E. Lester B. M. (2018). Early life stress and environmental influences on the neurodevelopment of children with prenatal opioid exposure. Neurobiol. Stress 9, 48–54. 10.1016/j.ynstr.2018.08.005 30151420 PMC6108075

[B75] Conradt E. Flannery T. Aschner J. L. Annett R. D. Croen L. A. Duarte C. S.. (2019). Prenatal opioid exposure: neurodevelopmental consequences and future research priorities. Pediatrics 144::e20190128. 10.1542/peds.2019-0128 31462446 PMC6759228

[B76] Conway C. R. Sheline Y. I. Chibnall J. T. George M. S. Fletcher J. W. Mintun M. A. (2006). Cerebral blood flow changes during vagus nerve stimulation for depression. Psychiatry Res. Neuroimaging 146, 179–184. 10.1016/j.pscychresns.2005.12.007 16510266

[B77] Corsello S. M. Paragliola R. M. (2019). Evaluation and management of endocrine hypertension during pregnancy. Endocrinol. Metab. Clin. North Am. 48, 829–842. 10.1016/j.ecl.2019.08.011 31655779

[B78] Crudo A. Suderman M. Moisiadis V. G. Petropoulos S. Kostaki A. Hallett M.. (2013). Glucocorticoid programming of the fetal male hippocampal epigenome. Endocrinology 154, 1168–1180. 10.1210/en.2012-1980 23389956

[B79] Davis D. C. (1996). The discomforts of pregnancy. J. Obstet. Gynecol. Neonatal Nurs. 25, 73–81. 10.1111/j.1552-6909.1996.tb02516.x 8627406

[B80] De Jonckheere J. Storme L. (2019). NIPE is related to parasympathetic activity. Is it also related to comfort? J. Clin. Monit. Comput. 33, 747–748. 10.1007/s10877-019-00276-1 30758689

[B81] De las Cuevas C. Sanz E. J. (2006). Safety of selective serotonin reuptake inhibitors in pregnancy. Curr. Drug Saf. 1, 17–24. 10.2174/157488606775252593 18690911

[B82] De Rogalski Landrot I. Roche F. Pichot V. Teyssier G. Gaspoz J.-M. Barthelemy J.-C.. (2007). Autonomic nervous system activity in premature and full-term infants from theoretical term to 7 years. Auton. Neurosci. 136, 105–109. 10.1016/j.autneu.2007.04.008 17556047

[B83] de Vries J. I. P. Visser G. H. A. Prechtl H. F. R. (1982). The emergence of fetal behaviour. I. Qualitative aspects. Early Hum. Dev. 7, 301–322. 10.1016/0378-3782(82)90033-0 7169027

[B84] de Vries J. I. P. Visser G. H. A. Prechtl H. F. R. (1985). The emergence of fetal behaviour. II. Quantitative aspects. Early Hum. Dev. 12, 99–120. 10.1016/0378-3782(85)90174-4 3905353

[B85] Dejmek J. Selevan S. G. Benes I. Solanský I. Srám R. J. (1999). Fetal growth and maternal exposure to particulate matter during pregnancy. Environ. Health Perspect. 107, 475–480. 10.1289/ehp.99107475 10339448 PMC1566587

[B86] Derbyshire S. W. Bockmann J. C. (2020). Reconsidering fetal pain. J. Med. Ethics 46, 3–6. 10.1136/medethics-2019-105701 31937669

[B87] Dereymaeker A. Pillay K. Vervisch J. De Vos M. Van Huffel S. Jansen K.. (2017). Review of sleep-EEG in preterm and term neonates. Early Hum. Dev. 113, 87–103. 10.1016/j.earlhumdev.2017.07.003 28711233 PMC6342258

[B88] Desrosiers T. A. Lawson C. C. Meyer R. E. Stewart P. A. Waters M. A. Correa A.. (2015). Assessed occupational exposure to chlorinated, aromatic and Stoddard solvents during pregnancy and risk of fetal growth restriction. Occup. Environ. Med. 72, 587–593. 10.1136/oemed-2015-102835 26076683 PMC4572733

[B89] Diamond B. Tracey K. J. (2011). Mapping the immunological homunculus. Proc. Natl. Acad. Sci. U.S.A. 108, 3461–3462. 10.1073/pnas.1100329108 21368214 PMC3048141

[B90] Dierckx B. Tulen J. H. M. van den Berg M. P. Tharner A. Jaddoe V. W. Moll H. A.. (2009). Maternal psychopathology influences infant heart rate variability: generation R study. Psychosom. Med. 71, 313–321. 10.1097/PSY.0b013e318198a82c 19251873

[B91] Dimitrijević L. Bjelaković B. Colović H. Mikov A. Živković V. Kocić M.. (2016). Assessment of general movements and heart rate variability in prediction of neurodevelopmental outcome in preterm infants. Early Hum. Dev. 99, 7–12. 10.1016/j.earlhumdev.2016.05.014 27372636

[B92] DiPietro J. A. Bornstein M. H. Hahn C.-S. Costigan K. Achy-Brou A. (2007). Fetal heart rate and variability: stability and prediction to developmental outcomes in early childhood. Child Dev. 78, 1788–1798. 10.1111/j.1467-8624.2007.01099.x 17988321 PMC2267766

[B93] DiPietro J. A. Caulfield L. E. Costigan K. A. Merialdi M. Nguyen R. H. N. Zavaleta N.. (2004). Fetal neurobehavioral development: a tale of two cities. Dev. Psychol. 40, 445–456. 10.1037/0012-1649.40.3.445 15122969

[B94] DiPietro J. A. Costigan K. A. Voegtline K. M. (2015). Studies in fetal behavior: revisited, renewed, and reimagined. Monogr. Soc. Res. Child Dev. 80, vii−94. 10.1111/mono.v80.3 26303396 PMC4835043

[B95] DiPietro J. A. Davis M. F. Costigan K. A. Barr D. B. (2014). Fetal heart rate and motor activity associations with maternal organochlorine levels: results of an exploratory study. J. Expo. Sci. Environ. Epidemiol. 24, 474–481. 10.1038/jes.2013.19 23591698 PMC4513653

[B96] Dogra Y. Suri V. Aggarwal N. Dogra R. K. (2019). Induction of labor with oxytocin in pregnancy with low-risk heart disease: a randomized controlled trial. Turk. J. Obstet. Gynecol. 16, 213–218. 10.4274/tjod.galenos.2019.59932 32231850 PMC7090258

[B97] Doussard-Roosevelt J. A. McClenny B. D. Porges S. W. (2001). Neonatal cardiac vagal tone and school-age developmental outcome in very low birth weight infants. Dev. Psychobiol. 38, 56–66. 11150061

[B98] Doussard-Roosevelt J. A. Porges S. W. Scanlon J. W. Alemi B. Scanlon K. B. (1997). Vagal regulation of heart rate in the prediction of developmental outcome for very low birth weight preterm infants. Child Dev. 68, 173–186. 10.2307/1131844 9179997

[B99] Doyle R. M. Harris K. Kamiza S. Harjunmaa U. Ashorn U. Nkhoma M.. (2017). Bacterial communities found in placental tissues are associated with severe chorioamnionitis and adverse birth outcomes. PLoS ONE 12:e0180167. 10.1371/journal.pone.0180167 28700642 PMC5507499

[B100] DuPont T. L. Chalak L. F. Morriss M. C. Burchfield P. J. Christie L. Sánchez P. J. (2013). Short-term outcomes of newborns with perinatal acidemia who are not eligible for systemic hypothermia therapy. J. Pediatr. 162, 35–41. 10.1016/j.jpeds.2012.06.042 22871488 PMC3712522

[B101] Durosier L. D. Green G. Batkin I. Seely A. J. Ross M. G. Richardson B. S.. (2014). Sampling rate of heart rate variability impacts the ability to detect acidemia in ovine fetuses near-term. Front. Pediatr. 2:38. 10.3389/fped.2014.00038 24829897 PMC4017161

[B102] Edlow A. G. (2017). Maternal obesity and neurodevelopmental and psychiatric disorders in offspring. Prenat. Diagn. 37, 95–110. 10.1002/pd.4932 27684946 PMC5572633

[B103] Edwards N. Middleton P. G. Blyton D. M. Sullivan C. E. (2002). Sleep disordered breathing and pregnancy. Thorax 57, 555–558. 10.1136/thorax.57.6.555 12037233 PMC1746354

[B104] Eid R. S. Chaiton J. A. Lieblich S. E. Bodnar T. S. Weinberg J. Galea L. A. M. (2019). Early and late effects of maternal experience on hippocampal neurogenesis, microglia, and the circulating cytokine milieu. Neurobiol. Aging 78, 1–17. 10.1016/j.neurobiolaging.2019.01.021 30825663

[B105] Faa G. Manchia M. Pintus R. Gerosa C. Marcialis M. A. Fanos V. (2016). Fetal programming of neuropsychiatric disorders. Birth Defects Res. C Embryo Today Rev. 108, 207–223. 10.1002/bdrc.21139 27774781

[B106] Facco F. L. Kramer J. Ho K. H. Zee P. C. Grobman W. A. (2010). Sleep disturbances in pregnancy. Obstet. Gynecol. 115, 77–83. 10.1097/AOG.0b013e3181c4f8ec 20027038

[B107] Fagard J. Esseily R. Jacquey L. O'Regan K. Somogyi E. (2018). Fetal origin of sensorimotor behavior. Front. Neurorobotics 12:23. 10.3389/fnbot.2018.00023 29875649 PMC5974044

[B108] Fairchild K. D. Srinivasan V. Randall Moorman J. Gaykema R. P. A. Goehler L. E. (2010). Pathogen-induced heart rate changes associated with cholinergic nervous system activation. Am. J. Physiol. Regul. Integr. Comp. Physiol. 300, R330–R339. 10.1152/ajpregu.00487.2010 21068197 PMC3043803

[B109] Fedele L. Brand T. (2020). The intrinsic cardiac nervous system and its role in cardiac pacemaking and conduction. J. Cardiovasc. Dev. Dis. 7, 54. 10.3390/jcdd7040054 33255284 PMC7712215

[B110] Fischer L. A. Demerath E. Bittner-Eddy P. Costalonga M. (2019). Placental colonization with periodontal pathogens: the potential missing link. Am. J. Obstet. Gynecol. 221, 383.e3–392.e3. 10.1016/j.ajog.2019.04.029 31051120 PMC6821581

[B111] Flenady V. Ellwood D. Bradford B. Coory M. Middleton P. Gardener G.. (2019). Beyond the headlines: fetal movement awareness is an important stillbirth prevention strategy. Women Birth 32, 1–2. 10.1016/j.wombi.2018.12.001 30563769

[B112] Fox G. P. P. Matthews T. G. (1989). Autonomic dysfunction at different ambient temperatures in infants at risk of sudden infant death syndrome. Lancet 334, 1065–1067. 10.1016/S0140-6736(89)91080-5 2572796

[B113] Franco P. Verheulpen D. Valente F. Kelmanson I. de Broca A. Scaillet S.. (2003). Autonomic responses to sighs in healthy infants and in victims of sudden infant death. Sleep Med. 4, 569–577. 10.1016/S1389-9457(03)00107-2 14607352

[B114] Frasch M. G. (2018). Saving the brain one heartbeat at a time: perspectives. J. Physiol. 596, 5503–5504. 10.1113/JP275776 29604062 PMC6265536

[B115] Frasch M. G. (2020). Heart rate variability code: does it exist and can we hack it? ArXiv200108264 Q-Bio. Available online at: http://arxiv.org/abs/2001.08264 (accessed April 23, 2021). 10.3390/bioengineering10070822 PMC10375964 37508849

[B116] Frasch M. G. Baier C. J. Antonelli M. C. Metz G. A. S. (2018a). Perinatal psychoneuroimmunology: protocols for the study of prenatal stress and its effects on fetal and postnatal brain development, in Psychoneuroimmunology Methods in Molecular Biology, ed Yan Q. (New York, NY: Springer New York), 353–376. 10.1007/978-1-4939-7828-1_19 29705857

[B117] Frasch M. G. Boylan G. B. Wu H.-T. Devane D. (2017). Commentary: Computerised interpretation of fetal heart rate during labour (INFANT): a randomised controlled trial. Front. Physiol. 8:721. 10.3389/fphys.2017.00721 29033845 PMC5625115

[B118] Frasch M. G. Burns P. Benito J. Cortes M. Cao M. Fecteau G.. (2018b). Sculpting the sculptors: methods for studying the fetal cholinergic signaling on systems and cellular scales. Methods Mol. Biol. 1781, 341–352. 10.1007/978-1-4939-7828-1_18 29705856

[B119] Frasch M. G. Giussani D. A. (2020). Impact of chronic fetal hypoxia and inflammation on cardiac pacemaker cell development. Cells 9:733. 10.3390/cells9030733 32192015 PMC7140710

[B120] Frasch M. G. Herry C. L. Niu Y. Giussani D. A. (2020a). First evidence that intrinsic fetal heart rate variability exists and is affected by hypoxic pregnancy. J. Physiol. 598, 249–263. 10.1113/JP278773 31802494

[B121] Frasch M. G. Keen A. E. Gagnon R. Ross M. G. Richardson B. S. (2011). Monitoring fetal electrocortical activity during labour for predicting worsening acidemia: a prospective study in the ovine fetus near term. PLoS ONE 6:e22100. 10.1371/journal.pone.0022100 21789218 PMC3137606

[B122] Frasch M. G. Lobmaier S. M. Stampalija T. Desplats P. Pallarés M. E. Pastor V.. (2020b). Non-invasive biomarkers of fetal brain development reflecting prenatal stress: an integrative multi-scale multi-species perspective on data collection and analysis. Neurosci. Biobehav. Rev. 117, 165–183. 10.1016/j.neubiorev.2018.05.026 29859198

[B123] Frasch M. G. Müller T. Hoyer D. Weiss C. Schubert H. Schwab M. (2009a). Nonlinear properties of vagal and sympathetic modulations of heart rate variability in ovine fetus near term. Am. J. Physiol. Regul. Integr. Comp. Physiol. 296, R702–R707. 10.1152/ajpregu.90474.2008 19109371

[B124] Frasch M. G. Müller T. Weiss C. Schwab K. Schubert H. Schwab M. (2009b). Heart rate variability analysis allows early asphyxia detection in ovine fetus. Reprod. Sci. 16, 509–517. 10.1177/1933719108327597 19164481

[B125] Frasch M. G. Müller T. Wicher C. Weiss C. Löhle M. Schwab K.. (2007). Fetal body weight and the development of the control of the cardiovascular system in fetal sheep. J. Physiol. 579, 893–907. 10.1113/jphysiol.2006.124800 17218361 PMC2151374

[B126] Frasch M. G. Shen C. Wu H.-T. Mueller A. Neuhaus E. Bernier R. A.. (2021). Brief report: can a composite heart rate variability biomarker shed new insights about autism spectrum disorder in school-aged children? J. Autism Dev. Disord. 51, 346–356. 10.1007/s10803-020-04467-7 32449059

[B127] Frasch M. G. Snir G. Antonelli M. C. (2019). Autism spectrum disorder: a neuro-immunometabolic hypothesis of the developmental origins. ArXiv190905198 Q-Bio. Available online at: http://arxiv.org/abs/1909.05198 (accessed May 13, 2021). 10.3390/biology12070914 PMC10375982 37508346

[B128] Frasch M. G. Szynkaruk M. Prout A. P. Nygard K. Cao M. Veldhuizen R.. (2016). Decreased neuroinflammation correlates to higher vagus nerve activity fluctuations in near-term ovine fetuses: a case for the afferent cholinergic anti-inflammatory pathway? J. Neuroinflammation 13:103. 10.1186/s12974-016-0567-x 27165310 PMC4894374

[B129] Freidman E. A. Sachtleben M. R. (1978). Effect of oxytocin and oral prostaglandin E2 on uterine contractility and fetal heart rate patterns. Am. J. Obstet. Gynecol. 130, 403–407. 10.1016/0002-9378(78)90280-6 629283

[B130] Fritze D. Zhang W. Li J.-Y. Chai B. Mulholland M. (2014). Thrombin mediates vagal apoptosis and dysfunction in inflammatory bowel disease. J. Gastrointest. Surg. 18, 1495–1506. 10.1007/s11605-014-2565-6 24916589

[B131] Fuhler G. M. (2020). The immune system and microbiome in pregnancy. Best Pract. Res. Clin. Gastroenterol. 44–45:101671. 10.1016/j.bpg.2020.101671 32359685

[B132] Gale C. R. Robinson S. M. Harvey N. C. Javaid M. K. Jiang B. Martyn C. N.. (2008). Maternal vitamin D status during pregnancy and child outcomes. Eur. J. Clin. Nutr. 62, 68–77. 10.1038/sj.ejcn.1602680 17311057 PMC2629513

[B133] Gallo-Padilla D. Gallo-Padilla C. Gallo-Vallejo F. J. Gallo-Vallejo J. L. (2016). [Low back pain during pregnancy. Multidisciplinary approach]. Semergen 42, e59–e64. 10.1016/j.semerg.2015.06.005 26239672

[B134] Gallup A. C. Church A. M. Pelegrino A. J. (2016). Yawn duration predicts brain weight and cortical neuron number in mammals. Biol. Lett. 12:20160545. 10.1098/rsbl.2016.0545 27703056 PMC5095190

[B135] Gallup A. C. Eldakar O. T. (2013). The thermoregulatory theory of yawning: what we know from over 5 years of research. Front. Neurosci. 6:188. 10.3389/fnins.2012.00188 23293583 PMC3534187

[B136] Gamer M. Büchel C. (2012). Oxytocin specifically enhances valence-dependent parasympathetic responses. Psychoneuroendocrinology 37, 87–93. 10.1016/j.psyneuen.2011.05.007 21641726

[B137] Gartstein M. A. Skinner M. K. (2018). Prenatal influences on temperament development: the role of environmental epigenetics. Dev. Psychopathol. 30, 1269–1303. 10.1017/S0954579417001730 29229018 PMC5997513

[B138] Garzoni L. Faure C. Frasch M. G. (2013). Fetal cholinergic anti-inflammatory pathway and necrotizing enterocolitis: the brain-gut connection begins *in utero*. Front. Integr. Neurosci. 7:57. 10.3389/fnint.2013.00057 23964209 PMC3737662

[B139] Gemmel M. De Lacalle S. Mort S. C. Hill L. A. Charlier T. D. Pawluski J. L. (2019). Perinatal fluoxetine has enduring sexually differentiated effects on neurobehavioral outcomes related to social behaviors. Neuropharmacology 144, 70–81. 10.1016/j.neuropharm.2018.10.009 30326241

[B140] Gernand A. D. Schulze K. J. Stewart C. P. West K. P. Christian P. (2016). Micronutrient deficiencies in pregnancy worldwide: health effects and prevention. Nat. Rev. Endocrinol. 12, 274–289. 10.1038/nrendo.2016.37 27032981 PMC4927329

[B141] Gintzler A. R. (1980). Endorphin-mediated increases in pain threshold during pregnancy. Science 210, 193–195. 10.1126/science.7414330 7414330

[B142] Glover V. (2015). Prenatal stress and its effects on the fetus and the child: possible underlying biological mechanisms, in Perinatal Programming of Neurodevelopment Advances in Neurobiology, ed Antonelli M. C. (New York, NY: Springer New York), 269–283. 10.1007/978-1-4939-1372-5_13 25287545

[B143] Goeden N. Velasquez J. Arnold K. A. Chan Y. Lund B. T. Anderson G. M.. (2016). Maternal inflammation disrupts fetal neurodevelopment via increased placental output of serotonin to the fetal brain. J. Neurosci. 36, 6041–6049. 10.1523/JNEUROSCI.2534-15.2016 27251625 PMC4887568

[B144] Gogou M. Haidopoulou K. Pavlou E. (2019). Sleep and prematurity: sleep outcomes in preterm children and influencing factors. World J. Pediatr. 15, 209–218. 10.1007/s12519-019-00240-8 30830664

[B145] Gokozan H. N. Baig F. Corcoran S. Catacutan F. P. Gygli P. E. Takakura A. C.. (2016). Area postrema undergoes dynamic postnatal changes in mice and humans: dynamic postnatal changes of area postrema. J. Comp. Neurol. 524, 1259–1269. 10.1002/cne.23903 26400711 PMC4747799

[B146] Gold N. Frasch M. G. (2021). Fetal cerebral perfusion is better than fetal acidaemia for the prediction of brain injury and might be assessable by sophisticated fetal heart rate metrics. BJOG Int. J. Obstet. Gynaecol. 128:1443. 10.1111/1471-0528.16674 33666319

[B147] Gold N. Herry C. L. Wang X. Frasch M. G. (2021). Fetal cardiovascular decompensation during labor predicted from the individual heart rate tracing: a machine learning approach in near-term fetal sheep model. Front. Pediatr. 9:593889. 10.3389/fped.2021.593889 34026680 PMC8132964

[B148] Goldaber K. G. Gilstrap L. C. Leveno K. J. Dax J. S. McIntire D. D. (1991). Pathologic fetal acidemia. Obstet. Gynecol. 78, 1103–1107 1945216

[B149] Goldenberg R. L. Culhane J. F. Iams J. D. Romero R. (2008). Epidemiology and causes of preterm birth. Lancet Lond. Engl. 371, 75–84. 10.1016/S0140-6736(08)60074-4 18177778 PMC7134569

[B150] Goldstein D. S. (2006). Adrenaline and the Inner World: An Introduction to Scientific Integrative Medicine. Baltimore, MD: Johns Hopkins University Press.

[B151] Gomes E. L. D. F. D. Santos C. M. D. Santos A. D. C. S. Silva A. G. D. França M. A. M. Romanini D. S.. (2019). Autonomic responses of premature newborns to body position and environmental noise in the neonatal intensive care unit. Rev. Bras. Ter. Intensiva 31, 296–302. 10.5935/0103-507X.20190054 31618347 PMC7005950

[B152] Gomez-Arango L. F. Barrett H. L. McIntyre H. D. Callaway L. K. Morrison M. Nitert M. D. (2017). Contributions of the maternal oral and gut microbiome to placental microbial colonization in overweight and obese pregnant women. Sci. Rep. 7:2860. 10.1038/s41598-017-03066-4 28588199 PMC5460277

[B153] Gonçalves H. Amorim-Costa C. Ayres-de-Campos D. Bernardes J. (2017). Gender-specific evolution of fetal heart rate variability throughout gestation: a study of 8823 cases. Early Hum. Dev. 115, 38–45. 10.1016/j.earlhumdev.2017.09.002 28889037

[B154] Gong W. Liang Q. Zheng D. Zhong R. Wen Y. Wang X. (2017). Congenital heart defects of fetus after maternal exposure to organic and inorganic environmental factors: a cohort study. Oncotarget 8, 100717–100723. 10.18632/oncotarget.20110 29246015 PMC5725057

[B155] Gordon C. (2004). Pregnancy and autoimmune diseases. Best Pract. Res. Clin. Rheumatol. 18, 359–379. 10.1016/j.berh.2004.02.012 15158746

[B156] Gotsch F. Romero R. Kusanovic J. P. Mazaki-Tovi S. Pineles B. L. Erez O.. (2007). The fetal inflammatory response syndrome. Clin. Obstet. Gynecol. 50, 652–683. 10.1097/GRF.0b013e31811ebef6 17762416

[B157] Grandjean P. Murata K. Budtz-Jørgensen E. Weihe P. (2004). Cardiac autonomic activity in methylmercury neurotoxicity: 14-year follow-up of a Faroese birth cohort. J. Pediatr. 144, 169–176. 10.1016/j.jpeds.2003.10.058 14760255

[B158] Graven S. N. Browne J. V. (2008). Sleep and brain development: the critical role of sleep in fetal and early neonatal brain development. Newborn Infant Nurs. Rev. 8, 173–179. 10.1053/j.nainr.2008.10.008

[B159] Gribble M. O. Cheng A. Berger R. D. Rosman L. Guallar E. (2015). Mercury exposure and heart rate variability: a systematic review. Curr. Environ. Health Rep. 2, 304–314. 10.1007/s40572-015-0053-0 26231507 PMC4800007

[B160] Gutke A. Betten C. Degerskär K. Pousette S. Olsén M. F. (2015). Treatments for pregnancy-related lumbopelvic pain: a systematic review of physiotherapy modalities. Acta Obstet. Gynecol. Scand. 94, 1156–1167. 10.1111/aogs.12681 26018758

[B161] Haley D. W. Handmaker N. S. Lowe J. (2006). Infant stress reactivity and prenatal alcohol exposure. Alcohol. Clin. Exp. Res. 30, 2055–2064. 10.1111/j.1530-0277.2006.00251.x 17117971

[B162] Han V. X. Patel S. Jones H. F. Nielsen T. C. Mohammad S. S. Hofer M. J.. (2021). Maternal acute and chronic inflammation in pregnancy is associated with common neurodevelopmental disorders: a systematic review. Transl. Psychiatry 11, 1–12. 10.1038/s41398-021-01198-w 33479207 PMC7820474

[B163] Hanamsagar R. Bilbo S. D. (2016). Sex differences in neurodevelopmental and neurodegenerative disorders: focus on microglial function and neuroinflammation during development. J. Steroid Biochem. Mol. Biol. 160, 127–133. 10.1016/j.jsbmb.2015.09.039 26435451 PMC4829467

[B164] Handal A. J. Harlow S. D. Breilh J. Lozoff B. (2008). Occupational exposure to pesticides during pregnancy and neurobehavioral development of infants and toddlers. Epidemiology 19, 851–859. 10.1097/EDE.0b013e318187cc5d 18813021

[B165] Hantoushzadeh S. Anvari Aliabad R. Norooznezhad A. H. (2020). Antibiotics, inflammation, and preterm labor: a missed conclusion. J. Inflamm. Res. 13, 245–254. 10.2147/JIR.S248382 32547156 PMC7261809

[B166] Haraldsdottir K. Watson A. M. Goss K. N. Beshish A. G. Pegelow D. F. Palta M.. (2018). Impaired autonomic function in adolescents born preterm. Physiol. Rep. 6:e13620. 10.14814/phy2.13620 29595875 PMC5875539

[B167] Harrison N. A. Cooper E. Voon V. Miles K. Critchley H. D. (2013). Central autonomic network mediates cardiovascular responses to acute inflammation: relevance to increased cardiovascular risk in depression? Brain. Behav. Immun. 31, 189–196. 10.1016/j.bbi.2013.02.001 23416033 PMC3701839

[B168] Hasain Z. Mokhtar N. M. Kamaruddin N. A. Mohamed Ismail N. A. Razalli N. H. Gnanou J. V.. (2020). Gut microbiota and gestational diabetes mellitus: a review of host-gut microbiota interactions and their therapeutic potential. Front. Cell. Infect. Microbiol. 10:188. 10.3389/fcimb.2020.00188 32500037 PMC7243459

[B169] Heazell A. E. P. Budd J. Li M. Cronin R. Bradford B. McCowan L. M. E.. (2018). Alterations in maternally perceived fetal movement and their association with late stillbirth: findings from the Midland and North of England stillbirth case–control study. BMJ Open 8:e020031. 10.1136/bmjopen-2017-020031 29982198 PMC6042603

[B170] Herry C. L. Burns P. Desrochers A. Fecteau G. Durosier L. D. Cao M.. (2019). Vagal contributions to fetal heart rate variability: an omics approach. Physiol. Meas. 40:065004. 10.1088/1361-6579/ab21ae 31091517

[B171] Herry C. L. Soares H. M. F. Schuler-Faccini L. Frasch M. G. (2021). Machine learning model on heart rate variability metrics identifies asymptomatic toddlers exposed to Zika virus during pregnancy. Physiol. Meas. 42:ac010e. 10.1088/1361-6579/ac010e 33984844

[B172] Hill M. A. (2021a). Cardiovascular System - Spleen Development - Embryology. Available online at: https://embryology.med.unsw.edu.au/embryology/index.php/Neural_System_Development (accessed April 23, 2021).

[B173] Hill M. A. (2021b). Neural System Development - Embryology. Available online at: https://embryology.med.unsw.edu.au/embryology/index.php/Neural_System_Development (Accessed April 23, 2021).

[B174] Hill M. A. (2021c). Respiratory System Development - Embryology. Available online at: https://embryology.med.unsw.edu.au/embryology/index.php/Neural_System_Development (Accessed April 23, 2021).

[B175] Hill M. A. (2021d). Timeline Human Development - Embryology. Available online at: https://embryology.med.unsw.edu.au/embryology/index.php/Neural_System_Development (accessed April 23, 2021).

[B176] Hillman N. H. Kallapur S. G. Jobe A. H. (2012). Physiology of transition from intrauterine to extrauterine life. Clin. Perinatol. 39, 769–783. 10.1016/j.clp.2012.09.009 23164177 PMC3504352

[B177] Hilton M. F. Umali M. U. Czeisler C. A. Wyatt J. K. Shea S. A. (2000). Endogenous circadian control of the human autonomic nervous system. Comput. Cardiol. 27, 197–200. 10.1109/CIC.2000.898490 14632012

[B178] Ho J. J. Subramaniam P. Davis P. G. (2020). Continuous positive airway pressure (CPAP) for respiratory distress in preterm infants. Cochrane Database Syst. Rev. 10:CD002271. 10.1002/14651858.CD002271.pub3 33058208 PMC8094155

[B179] Holsti L. Grunau R. E. Whifield M. F. Oberlander T. F. Lindh V. (2006). Behavioral responses to pain are heightened after clustered care in preterm infants born between 30 and 32 weeks gestational age. Clin. J. Pain 22, 757–764. 10.1097/01.ajp.0000210921.10912.47 17057556 PMC1851898

[B180] Hon E. H. Lee S. T. (1963). Electronic evaluation of the fetal heart rate. VIII. Patterns preceding fetal death, further observations. Am. J. Obstet. Gynecol. 87, 814–826. 14085784

[B181] Horne R. S. (2018). Autonomic cardiorespiratory physiology and arousal of the fetus and infant, in SIDS Sudden Infant and Early Childhood Death: The Past, the Present and the Future, eds Duncan J. R. Byard R. W. (Adelaide, AU: University of Adelaide Press). Available online at: http://www.ncbi.nlm.nih.gov/books/NBK513398/ (accessed April 23, 2021).

[B182] Howland M. A. Sandman C. A. Davis E. P. Glynn L. M. (2020). Prenatal maternal psychological distress and fetal developmental trajectories: associations with infant temperament. Dev. Psychopathol. 32, 1685–1695. 10.1017/S095457942000142X 33427168 PMC8643070

[B183] Hoyer D. Kowalski E.-M. Schmidt A. Tetschke F. Nowack S. Rudolph A.. (2014). Fetal autonomic brain age scores, segmented heart rate variability analysis, and traditional short term variability. Front. Hum. Neurosci. 8:948. 10.3389/fnhum.2014.00948 25505399 PMC4243554

[B184] Hoyer D. Nowack S. Bauer S. Tetschke F. Ludwig S. Moraru L.. (2012). Fetal development assessed by heart rate patterns—time scales of complex autonomic control. Comput. Biol. Med. 42, 335–341. 10.1016/j.compbiomed.2011.05.003 21621201

[B185] Hoyer D. Nowack S. Bauer S. Tetschke F. Rudolph A. Wallwitz U.. (2013a). Fetal development of complex autonomic control evaluated from multiscale heart rate patterns. Am. J. Physiol. Regul. Integr. Comp. Physiol. 304, R383–R392. 10.1152/ajpregu.00120.2012 23269479

[B186] Hoyer D. Schmidt A. Gustafson K. M. Lobmaier S. M. Lakhno I. van Leeuwen P.. (2019). Heart rate variability categories of fluctuation amplitude and complexity: diagnostic markers of fetal development and its disturbances. Physiol. Meas. 40:064002. 10.1088/1361-6579/ab205f 31071684 PMC7020698

[B187] Hoyer D. Schneider U. Kowalski E.-M. Schmidt A. Witte O. W. Schleußner E.. (2015). Validation of functional fetal autonomic brain age score fABAS in 5 min short recordings. Physiol. Meas. 36, 2369–2378. 10.1088/0967-3334/36/11/2369 26489779

[B188] Hoyer D. Tetschke F. Jaekel S. Nowack S. Witte O. W. Schleußner E.. (2013b). Fetal functional brain age assessed from universal developmental indices obtained from neuro-vegetative activity patterns. PLOS ONE 8:e74431. 10.1371/journal.pone.0074431 24058564 PMC3776847

[B189] Hoyer D. Zebrowski J. Cysarz D. Gonçalves H. Pytlik A. Amorim-Costa C.. (2017). Monitoring fetal maturation—objectives, techniques and indices of autonomic function. Physiol. Meas. 38, R61–R88. 10.1088/1361-6579/aa5fca 28186000 PMC5628752

[B190] Hutchins E. J. Kunttas E. Piacentino M. L. Howard A. G. A. Bronner M. E. Uribe R. A. (2018). Migration and diversification of the vagal neural crest. Dev. Biol. 444, S98–S109. 10.1016/j.ydbio.2018.07.004 29981692 PMC6320731

[B191] Hutchison J. Mahdy H. Hutchison J. (2021). Stages of labor, in StatPearls (Treasure Island, FL: StatPearls Publishing). Available online at: http://www.ncbi.nlm.nih.gov/books/NBK544290/ (accessed April 8, 2021).

[B192] Ingemarsson I. Lamont R. F. (2003). An update on the controversies of tocolytic therapy for the prevention of preterm birth. Acta Obstet. Gynecol. Scand. 82, 1–9. 10.1080/j.1600-0412.2003.820101.x 12580832

[B193] Jansson L. M. DiPietro J. A. Velez M. Elko A. Williams E. Milio L.. (2011). Fetal neurobehavioral effects of exposure to methadone or buprenorphine. Neurotoxicol. Teratol. 33, 240–243. 10.1016/j.ntt.2010.09.003 20868741 PMC3040781

[B194] Jarczok M. N. Aguilar-Raab C. Koenig J. Kaess M. Borniger J. C. Nelson R. J.. (2018). The Heart's rhythm ‘n’ blues: sex differences in circadian variation patterns of vagal activity vary by depressive symptoms in predominantly healthy employees. Chronobiol. Int. 35, 896–909. 10.1080/07420528.2018.1439499 29543518

[B195] Jarrell J. (2017). Pregnancy-related pain. J. Obstet. Gynecol. Can. 39, 769–771. 10.1016/j.jogc.2017.05.029 28733064

[B196] Jonas W. Johansson L. M. Nissen E. Ejdebäck M. Ransjö-Arvidson A. B. Uvnäs-Moberg K. (2009). Effects of intrapartum oxytocin administration and epidural analgesia on the concentration of plasma oxytocin and prolactin, in response to suckling during the second day postpartum. Breastfeed. Med. Off. J. Acad. Breastfeed. Med. 4, 71–82. 10.1089/bfm.2008.0002 19210132

[B197] Jones C. M. Greiss F. C. (1982). The effect of labor on maternal and fetal circulating catecholamines. Am. J. Obstet. Gynecol. 144, 149–153. 10.1016/0002-9378(82)90616-0 7114122

[B198] Jonsson M. Nordén-Lindeberg S. Ostlund I. Hanson U. (2008). Acidemia at birth, related to obstetric characteristics and to oxytocin use, during the last two hours of labor. Acta Obstet. Gynecol. Scand. 87, 745–750. 10.1080/00016340802220352 18607817

[B199] Kadhim H. Tabarki B. Verellen G. De Prez C. Rona A. M. Sébire G. (2001). Inflammatory cytokines in the pathogenesis of periventricular leukomalacia. Neurology 56, 1278–1284. 10.1212/wnl.56.10.1278 11376173

[B200] Kamendi H. Stephens C. Dergacheva O. Wang X. Huang Z.-G. Bouairi E.. (2006). Prenatal nicotine exposure alters the nicotinic receptor subtypes that modulate excitation of parasympathetic cardiac neurons in the nucleus ambiguus from primarily α3β2 and/or α6βX to α3β4. Neuropharmacology 51, 60–66. 10.1016/j.neuropharm.2006.03.001 16690087

[B201] Kamendi H. W. Cheng Q. Dergacheva O. Gorini C. Jameson H. S. Wang X.. (2009). Abolishment of serotonergic neurotransmission to cardiac vagal neurons during and after hypoxia and hypercapnia with prenatal nicotine exposure. J. Neurophysiol. 101, 1141–1150. 10.1152/jn.90680.2008 19091927 PMC3815238

[B202] Kamitomo M. Sameshima H. Ikenoue T. Nishibatake M. (2000). Fetal cardiovascular function during prolonged magnesium sulfate tocolysis. J. Perinat. Med. 28, 377–382. 10.1515/JPM.2000.048 11125928

[B203] Kapaya H. Broughton-Pipkin F. Hayes-Gill B. Loughna P. V. (2015). Smoking in pregnancy affects the fetal heart: possible links to future cardiovascular disease. J. Matern. Fetal Neonatal Med. 28, 1664–1668. 10.3109/14767058.2014.964202 25212975

[B204] Kariniemi V. ÄMmälä P. (1981). Effects of intramuscular pethidine on fetal heart rate variability during labour. BJOG Int. J. Obstet. Gynaecol. 88, 718–720. 10.1111/j.1471-0528.1981.tb01271.x 7248229

[B205] Karita K. Iwata T. Maeda E. Sakamoto M. Murata K. (2018). Assessment of cardiac autonomic function in relation to methylmercury neurotoxicity. Toxics 6:38. 10.3390/toxics6030038 30036985 PMC6161203

[B206] Kasahara Y. Yoshida C. Nakanishi K. Fukase M. Suzuki A. Kimura Y. (2020). Alterations in the autonomic nerve activities of prenatal autism model mice treated with valproic acid at different developmental stages. Sci. Rep. 10:17722. 10.1038/s41598-020-74662-0 33082409 PMC7576159

[B207] Kasahara Y. Yoshida C. Saito M. Kimura Y. (2021). Assessments of heart rate and sympathetic and parasympathetic nervous activities of normal mouse fetuses at different stages of fetal development using fetal electrocardiography. Front. Physiol. 12:652828. 10.3389/fphys.2021.652828 33897461 PMC8061630

[B208] Kentish S. J. Frisby C. L. Kennaway D. J. Wittert G. A. Page A. J. (2013). Circadian variation in gastric vagal afferent mechanosensitivity. J. Neurosci. 33, 19238–19242. 10.1523/JNEUROSCI.3846-13.2013 24305819 PMC6618780

[B209] Khedun S. M. Maharaj B. Moodley J. (2000). Effects of antihypertensive drugs on the unborn child. Paediatr. Drugs 2, 419–436. 10.2165/00128072-200002060-00002 11127843

[B210] Kisilevsky B. S. Low J. A. (1998). Human fetal behavior: 100 years of study. Dev. Rev. 18, 1–29. 10.1006/drev.1998.0452

[B211] Koletzko B. Cremer M. Flothkötter M. Graf C. Hauner H. Hellmers C.. (2018). Diet and lifestyle before and during pregnancy – practical recommendations of the germany-wide healthy start – young family network. Geburtsh. Frauenh. 78, 1262–1282. 10.1055/a-0713-1058 30655650 PMC6294644

[B212] Koletzko B. Godfrey K. M. Poston L. Szajewska H. van Goudoever J. B. de Waard M.. (2019). Nutrition during pregnancy, lactation and early childhood and its implications for maternal and long-term child health: the early nutrition project recommendations. Ann. Nutr. Metab. 74, 93–106. 10.1159/000496471 30673669 PMC6397768

[B213] Koolhaas J. M. Bartolomucci A. Buwalda B. de Boer S. F. Flügge G. Korte S. M.. (2011). Stress revisited: a critical evaluation of the stress concept. Neurosci. Biobehav. Rev. 35, 1291–1301. 10.1016/j.neubiorev.2011.02.003 21316391

[B214] Korsmo H. W. Jiang X. Caudill M. A. (2019). Choline: exploring the growing science on its benefits for moms and babies. Nutrients 11:1823. 10.3390/nu11081823 31394787 PMC6722688

[B215] Kozar M. Tonhajzerova I. Mestanik M. Matasova K. Zibolen M. Calkovska A.. (2018). Heart rate variability in healthy term newborns is related to delivery mode: a prospective observational study. BMC Pregnancy Childbirth 18:264. 10.1186/s12884-018-1900-4 29945544 PMC6020381

[B216] Krueger C. Garvan C. (2019). Cardiac orienting to auditory stimulation in the fetus. SAGE Open Nurs. 5:2377960819861486. 10.1177/2377960819861486 33415245 PMC7774446

[B217] Kruepunga N. Hikspoors J. P. J. M. Hülsman C. J. M. Mommen G. M. C. Köhler S. E. Lamers W. H. (2020). Development of extrinsic innervation in the abdominal intestines of human embryos. J. Anat. 237, 655–671. 10.1111/joa.13230 32598482 PMC7495293

[B218] Kruepunga N. Hikspoors J. P. J. M. Hülsman C. J. M. Mommen G. M. C. Köhler S. E. Lamers W. H. (2021). Development of the sympathetic trunks in human embryos. J. Anat., joa. 13415. 10.1111/joa.13415 33641166 PMC8197954

[B219] Kuperman A. A. Zimmerman A. Hamadia S. Ziv O. Gurevich V. Fichtman B.. (2020). Deep microbial analysis of multiple placentas shows no evidence for a placental microbiome. BJOG Int. J. Obstet. Gynaecol. 127, 159–169. 10.1111/1471-0528.15896 31376240

[B220] Lacasaña M. Vázquez-Grameix H. Borja-Aburto V. H. Blanco-Muñoz J. Romieu I. Aguilar-Garduño C.. (2006). Maternal and paternal occupational exposure to agricultural work and the risk of anencephaly. Occup. Environ. Med. 63, 649–656. 10.1136/oem.2005.023333 16873458 PMC2078046

[B221] Ladfors L. Thiringer K. Niklasson A. Odeback A. Thornberg E. (2002). Influence of maternal, obstetric and fetal risk factors on the prevalence of birth asphyxia at term in a Swedish urban population. Acta Obstet. Gynecol. Scand. 81, 909–917. 10.1080/j.1600-0412.2002.811003.x 12366480

[B222] Lange S. Van Leeuwen P. Geue D. Hatzmann W. Grönemeyer D. (2005). Influence of gestational age, heart rate, gender and time of day on fetal heart rate variability. Med. Biol. Eng. Comput. 43, 481–486. 10.1007/BF02344729 16255430

[B223] Langlois P. H. Hoyt A. T. Lupo P. J. Lawson C. C. Waters M. A. Desrosiers T. A.. (2012). Maternal occupational exposure to polycyclic aromatic hydrocarbons and risk of neural tube defect-affected pregnancies. Birt. Defects Res. A Clin. Mol. Teratol. 94, 693–700. 10.1002/bdra.23045 22807044 PMC5048886

[B224] Lattimore K. A. Donn S. M. Kaciroti N. Kemper A. R. Neal C. R. Vazquez D. M. (2005). Selective serotonin reuptake inhibitor (SSRI) use during pregnancy and effects on the fetus and newborn: a meta-analysis. J. Perinatol. 25, 595–604. 10.1038/sj.jp.7211352 16015372

[B225] Lauder A. P. Roche A. M. Sherrill-Mix S. Bailey A. Laughlin A. L. Bittinger K.. (2016). Comparison of placenta samples with contamination controls does not provide evidence for a distinct placenta microbiota. Microbiome 4:29. 10.1186/s40168-016-0172-3 27338728 PMC4917942

[B226] Laursen M. F. Bahl M. I. Michaelsen K. F. Licht T. R. (2017). First Foods and Gut Microbes. Front. Microbiol. 8:356. 10.3389/fmicb.2017.00356 28321211 PMC5337510

[B227] Lavin J. P. (1982). The effects of epidural anesthesia on electronic fetal heart rate monitoring. Clin. Perinatol. 9, 55–62 7067326

[B228] Lecanuet J. P. Granier-Deferre C. Jacquet A. Y. Busnel M. C. (1992). Decelerative cardiac responsiveness to acoustical stimulation in the near term fetus. Q. J. Exp. Psychol. B 44, 279–303. 10.1080/02724999208250616 1598423

[B229] Ledger W. J. Blaser M. J. (2013). Are we using too many antibiotics during pregnancy? BJOG Int. J. Obstet. Gynaecol. 120, 1450–1452. 10.1111/1471-0528.12371 24118809 PMC4492536

[B230] Lee K. A. Gay C. L. (2004). Sleep in late pregnancy predicts length of labor and type of delivery. Am. J. Obstet. Gynecol. 191, 2041–2046. 10.1016/j.ajog.2004.05.086 15592289

[B231] Leiby J. S. McCormick K. Sherrill-Mix S. Clarke E. L. Kessler L. R. Taylor L. J.. (2018). Lack of detection of a human placenta microbiome in samples from preterm and term deliveries. Microbiome 6:196. 10.1186/s40168-018-0575-4 30376898 PMC6208038

[B232] Lemyre B. Chau V. (2018). Hypothermia for newborns with hypoxic-ischemic encephalopathy. Paediatr. Child Health 23, 285–291. 10.1093/pch/pxy028 30657134 PMC6007306

[B233] Liu H. L. Garzoni L. Herry C. Durosier L. D. Cao M. Burns P.. (2016). Can monitoring fetal intestinal inflammation using heart rate variability analysis signal incipient necrotizing enterocolitis of the neonate? Pediatr. Crit. Care Med. 17, e165–e176. 10.1097/PCC.0000000000000643 26914621

[B234] Lobmaier S. M. Müller A. Zelgert C. Shen C. Su P. C. Schmidt G.. (2020). Fetal heart rate variability responsiveness to maternal stress, non-invasively detected from maternal transabdominal ECG. Arch. Gynecol. Obstet. 301, 405–414. 10.1007/s00404-019-05390-8 31781889

[B235] Low J. A. Cox M. J. Karchmar E. J. McGrath M. J. Pancham S. R. Piercy W. N. (1981). The effect of maternal, labor, and fetal factors upon fetal heart rate during the intrapartum period. Am. J. Obstet. Gynecol. 139, 306–310. 10.1016/0002-9378(81)90015-6 7468699

[B236] Lucchini M. Fifer W. P. Sahni R. Signorini M. G. (2016). Novel heart rate parameters for the assessment of autonomic nervous system function in premature infants. Physiol. Meas. 37, 1436–1446. 10.1088/0967-3334/37/9/1436 27480495 PMC5021440

[B237] Lu-Culligan A. Iwasaki A. (2020). The role of immune factors in shaping fetal neurodevelopment. Annu. Rev. Cell Dev. Biol. 36, 441–468. 10.1146/annurev-cellbio-021120-033518 32722920 PMC9034439

[B238] Lutterodt M. C. Kähler P. Kragstrup J. Nicolaisdottir D. R. Siersma V. Ertmann R. K. (2019). Examining to what extent pregnancy-related physical symptoms worry women in the first trimester of pregnancy: a cross-sectional study in general practice. BJGP Open 3:bjgpopen19X101674. 10.3399/bjgpopen19X101674 31719117 PMC6995862

[B239] Mackenzie J. Murray E. Lusher J. (2018). Women's experiences of pregnancy related pelvic girdle pain: a systematic review. Midwifery 56, 102–111. 10.1016/j.midw.2017.10.011 29096278

[B240] Macklem P. T. Seely A. (2010). Towards a definition of life. Perspect. Biol. Med. 53, 330–340. 10.1353/pbm.0.0167 20639603

[B241] Malpas S. C. Purdie G. L. (1990). Circadian variation of heart rate variability. Cardiovasc. Res. 24, 210–213. 10.1093/cvr/24.3.210 2346954

[B242] Manzotti A. Cerritelli F. Esteves J. E. Lista G. Lombardi E. La Rocca S.. (2019). Dynamic touch reduces physiological arousal in preterm infants: a role for c-tactile afferents? Dev. Cogn. Neurosci. 39:100703. 10.1016/j.dcn.2019.100703 31487608 PMC6969366

[B243] Martens D. S. Capozzoli M. Cox B. Nawrot T. S. (2019). Early biological aging and fetal exposure to high and low ambient temperature: a birth cohort study. Environ. Health Perspect. 127:117001. 10.1289/EHP5153 31691586 PMC6927502

[B244] Marzbanrad F. Khandoker A. H. Kimura Y. Palaniswami M. Clifford G. D. (2017). Assessment of fetal development using cardiac valve intervals. Front. Physiol. 8:313. 10.3389/fphys.2017.00313 28567021 PMC5434138

[B245] Massaro A. N. Campbell H. E. Metzler M. Al-Shargabi T. Wang Y. du Plessis A.. (2017). Effect of temperature on heart rate variability in neonatal intensive care unit patients with hypoxic ischemic encephalopathy. Pediatr. Crit. Care Med. 18, 349–354. 10.1097/PCC.0000000000001094 28198757 PMC5402340

[B246] Mattingly J. E. D'Alessio J. Ramanathan J. (2003). Effects of obstetric analgesics and anesthetics on the neonate. Pediatr. Drugs 5, 615–627. 10.2165/00148581-200305090-00004 12956618

[B247] McCarthy J. J. Leamon M. H. Finnegan L. P. Fassbender C. (2017). Opioid dependence and pregnancy: minimizing stress on the fetal brain. Am. J. Obstet. Gynecol. 216, 226–231. 10.1016/j.ajog.2016.10.003 27729254

[B248] McEwen B. S. Nasca C. Gray J. D. (2016). Stress effects on neuronal structure: hippocampus, amygdala, and prefrontal cortex. Neuropsychopharmacol. Off. Publ. Am. Coll. Neuropsychopharmacol. 41, 3–23. 10.1038/npp.2015.171 26076834 PMC4677120

[B249] McKenna D. S. Ventolini G. Neiger R. Downing C. (2006). Gender-related differences in fetal heart rate during first trimester. Fetal Diagn. Ther. 21, 144–147. 10.1159/000089065 16354993

[B250] McMahon E. Wintermark P. Lahav A. (2012). Auditory brain development in premature infants: the importance of early experience: McMahon et al. Ann. N. Y. Acad. Sci. 1252, 17–24. 10.1111/j.1749-6632.2012.06445.x 22524335

[B251] Mercer B. M. Carr T. L. Beazley D. D. Crouse D. T. Sibai B. M. (1999). Antibiotic use in pregnancy and drug-resistant infant sepsis. Am. J. Obstet. Gynecol. 181, 816–821. 10.1016/S0002-9378(99)70307-8 10521735

[B252] Metzler M. Govindan R. Al-Shargabi T. Vezina G. Andescavage N. Wang Y.. (2017). Pattern of brain injury and depressed heart rate variability in newborns with hypoxic ischemic encephalopathy. Pediatr. Res. 82, 438–443. 10.1038/pr.2017.94 28376079 PMC5570625

[B253] Meyer U. Yee B. K. Feldon J. (2007). The neurodevelopmental impact of prenatal infections at different times of pregnancy: the earlier the worse? Neuroscientist 13, 241–256. 10.1177/1073858406296401 17519367

[B254] Micheli K. Komninos I. Bagkeris E. Roumeliotaki T. Koutis A. Kogevinas M.. (2011). Sleep patterns in late pregnancy and risk of preterm birth and fetal growth restriction. Epidemiology 22, 738–744. 10.1097/EDE.0b013e31822546fd 21734587

[B255] Miljković Z. Sabo A. Stanulović M. Jakovljević V. Grujić I. (2001). [Drug use during pregnancy, labor and the puerperium and after the drug use in pregnancy study]. Med. Pregl. 54, 34–37. 11432320

[B256] Mindell J. A. Cook R. A. Nikolovski J. (2015). Sleep patterns and sleep disturbances across pregnancy. Sleep Med. 16, 483–488. 10.1016/j.sleep.2014.12.006 25666847

[B257] Mirmiran M. Maas Y. G. H. Ariagno R. L. (2003). Development of fetal and neonatal sleep and circadian rhythms. Sleep Med. Rev. 7, 321–334. 10.1053/smrv.2002.0243 14505599

[B258] Montagna A. Nosarti C. (2016). Socio-emotional development following very preterm birth: pathways to psychopathology. Front. Psychol. 7:80. 10.3389/fpsyg.2016.00080 26903895 PMC4751757

[B259] Moore K. L. Persaud T. V. N. Torchia M. G. (2016). The Developing Human: Clinically Oriented Embryology, 10th Edn. Philadelphia, PA: Elsevier.

[B260] Moors S. Staaks K. J. J. Westerhuis M. E. M. H. Dekker L. R. C. Verdurmen K. M. J. Oei S. G.. (2020). Heart rate variability in hypertensive pregnancy disorders: a systematic review. Pregnancy Hypertens. 20, 56–68. 10.1016/j.preghy.2020.03.003 32179490

[B261] Moreira Neto R. Porovic S. (2018). Clinical study of fetal neurobehavior by the KANET test. J. Perinat. Med. 46, 631–639. 10.1515/jpm-2016-0414 29306932

[B262] Morris G. S. Zhou Q. Hegsted M. Keenan M. J. (1995). Maternal consumption of a low vitamin D diet retards metabolic and contractile development in the neonatal rat heart. J. Mol. Cell. Cardiol. 27, 1245–1250. 10.1016/s0022-2828(05)82386-7 8531206

[B263] Morrison J. L. Berry M. J. Botting K. J. Darby J. R. T. Frasch M. G. Gatford K. L.. (2018). Improving pregnancy outcomes in humans through studies in sheep. Am. J. Physiol. Regul. Integr. Comp. Physiol. 315, R1123–R1153. 10.1152/ajpregu.00391.2017 30325659

[B264] Morrison J. L. Regnault T. R. H. (2016). Nutrition in pregnancy: optimising maternal diet and fetal adaptations to altered nutrient supply. Nutrients 8:342. 10.3390/nu8060342 27271666 PMC4924183

[B265] Morrison J. L. Riggs K. W. Rurak D. W. Morrison J. L. Riggs K. W. Rurak D. W. (2005). Fluoxetine during pregnancy: impact on fetal development. Reprod. Fertil. Dev. 17, 641–650. 10.1071/RD05030 16263070

[B266] Morsi A. DeFranco D. Witchel S. F. (2018). The hypothalamic-pituitary-adrenal axis and the fetus. Horm. Res. Paediatr. 89, 380–387. 10.1159/000488106 29874660

[B267] Morton S. U. Brodsky D. (2016). Fetal physiology and the transition to extrauterine life. Clin. Perinatol. 43, 395–407. 10.1016/j.clp.2016.04.001 27524443 PMC4987541

[B268] Mowery N. T. Morris J. A. Jenkins J. M. Ozdas A. Norris P. R. (2011). Core temperature variation is associated with heart rate variability independent of cardiac index: a study of 278 trauma patients. J. Crit. Care 26, 534.e9–534.e17. 10.1016/j.jcrc.2010.11.008 21376520

[B269] Mulder E. J. H. Derks J. B. Visser G. H. A. (2004). Effects of antenatal betamethasone administration on fetal heart rate and behavior in twin pregnancy. Pediatr. Res. 56, 35–39. 10.1203/01.PDR.0000130476.97700.2B 15128914

[B270] Mulkey S. B. du Plessis A. (2018). The critical role of the central autonomic nervous system in fetal-neonatal transition. Semin. Pediatr. Neurol. 28, 29–37. 10.1016/j.spen.2018.05.004 30522725 PMC6432941

[B271] Mulkey S. B. du Plessis A. J. (2019). Autonomic nervous system development and its impact on neuropsychiatric outcome. Pediatr. Res. 85, 120–126. 10.1038/s41390-018-0155-0 30166644 PMC6353676

[B272] Mulkey S. B. Hitchings L. Persaud R. Kota S. Maxwell G. L. Baker R.. (2021). Cerebral cortical autonomic connectivity in low-risk term newborns. Clin. Auton. Res. 31, 415–424. 10.1007/s10286-021-00793-7 33718981 PMC8603422

[B273] Mulkey S. B. Kota S. Govindan R. B. Al-Shargabi T. Swisher C. B. Eze A.. (2019). The effect of labor and delivery mode on electrocortical and brainstem autonomic function during neonatal transition. Sci. Rep. 9:11020. 10.1038/s41598-019-47306-1 31363124 PMC6667470

[B274] Mulkey S. B. Kota S. Swisher C. B. Hitchings L. Metzler M. Wang Y.. (2018). Autonomic nervous system depression at term in neurologically normal premature infants. Early Hum. Dev. 123, 11–16. 10.1016/j.earlhumdev.2018.07.003 30025221 PMC6136650

[B275] Müller F. O'Rahilly R. (1989). The human brain at stage 16, including the initial evagination of the neurohypophysis. Anat. Embryol. (Berl.) 179, 551–569. 10.1007/BF00315698 2751117

[B276] Multon O. Senat M.-V. Minoui S. Hue M.-V. Frydman R. Ville Y. (1997). Effect of antenatal betamethasone and dexamethasone administration on fetal heart rate variability in growth-retarded fetuses. Fetal Diagn. Ther. 12, 170–177. 10.1159/000264461 9313077

[B277] Murata K. Sakamoto M. Nakai K. Dakeishi M. Iwata T. Liu X.-J.. (2006). Subclinical effects of prenatal methylmercury exposure on cardiac autonomic function in Japanese children. Int. Arch. Occup. Environ. Health 79, 379–386. 10.1007/s00420-005-0064-5 16365750

[B278] Musa S. M. Adam I. Hassan N. G. Rayis D. A. Lutfi M. F. (2017). Maternal heart rate variability during the first stage of labor. Front. Physiol. 8:774. 10.3389/fphys.2017.00774 29062281 PMC5640770

[B279] Nalli C. Iodice A. Andreoli L. Galli J. Lojacono A. Motta M.. (2017). Long-term neurodevelopmental outcome of children born to prospectively followed pregnancies of women with systemic lupus erythematosus and/or antiphospholipid syndrome. Lupus 26, 552–558. 10.1177/0961203317694960 28394231

[B280] Nemoda Z. Massart R. Suderman M. Hallett M. Li T. Coote M.. (2015). Maternal depression is associated with DNA methylation changes in cord blood T lymphocytes and adult hippocampi. Transl. Psychiatry 5:e545. 10.1038/tp.2015.32 25849984 PMC4462598

[B281] Nensi A. De Silva D. A. von Dadelszen P. Sawchuck D. Synnes A. R. Crane J.. (2014). Effect of magnesium sulphate on fetal heart rate parameters: a systematic review. J. Obstet. Gynaecol. Can. 36, 1055–1064. 10.1016/S1701-2163(15)30382-0 25668040

[B282] Newbern J. M. (2015). Molecular control of the neural crest and peripheral nervous system development. Curr. Top. Dev. Biol. 111, 201–231. 10.1016/bs.ctdb.2014.11.007 25662262 PMC4517674

[B283] Nguyen T. A. Chow T. Riggs W. Rurak D. (2019). Postnatal outcomes in lambs exposed antenatally and acutely postnatally to fluoxetine. Pediatr. Res. 85, 1032–1040. 10.1038/s41390-019-0309-8 30739124

[B284] Noben L. Verdurmen K. M. J. Warmerdam G. J. J. Vullings R. Oei S. G. van Laar J. O. E. H. (2019). The fetal electrocardiogram to detect the effects of betamethasone on fetal heart rate variability. Early Hum. Dev. 130, 57–64. 10.1016/j.earlhumdev.2019.01.011 30677639

[B285] Nordenstam F. Norman M. Wickström R. (2019). Blood pressure and heart rate variability in preschool children exposed to smokeless tobacco in fetal life. J. Am. Heart Assoc. 8:e012629. 10.1161/JAHA.119.012629 31615305 PMC6898855

[B286] Norman G. J. Cacioppo J. T. Morris J. S. Malarkey W. B. Berntson G. G. Devries A. C. (2011). Oxytocin increases autonomic cardiac control: moderation by loneliness. Biol. Psychol. 86, 174–180. 10.1016/j.biopsycho.2010.11.006 21126557

[B287] Nugent B. M. Bale T. L. (2015). The omniscient placenta: metabolic and epigenetic regulation of fetal programming. Front. Neuroendocrinol. 39, 28–37. 10.1016/j.yfrne.2015.09.001 26368654 PMC4681645

[B288] Nuriel-Ohayon M. Neuman H. Koren O. (2016). Microbial changes during pregnancy, birth, and infancy. Front. Microbiol. 7:1031. 10.3389/fmicb.2016.01031 27471494 PMC4943946

[B289] Oberlander T. F. Gingrich J. A. Ansorge M. S. (2009). Sustained neurobehavioral effects of exposure to SSRI antidepressants during development: molecular to clinical evidence. Clin. Pharmacol. Ther. 86, 672–677. 10.1038/clpt.2009.201 19890255 PMC3963518

[B290] Oberlander T. F. Jacobson S. W. Weinberg J. Grunau R. E. Molteno C. D. Jacobson J. L. (2010). Prenatal alcohol exposure alters biobehavioral reactivity to pain in newborns. Alcohol. Clin. Exp. Res. 34, 681–692. 10.1111/j.1530-0277.2009.01137.x 20121718 PMC4833444

[B291] Oguch O. Steer P. (1998). Gender does not affect fetal heart rate variation. Br. J. Obstet. Gynaecol. 105, 1312–1314. 10.1111/j.1471-0528.1998.tb10011.x 9883924

[B292] Olaniyi K. S. Moodley J. Mahabeer Y. Mackraj I. (2020). Placental microbial colonization and its association with pre-eclampsia. Front. Cell. Infect. Microbiol. 10:413. 10.3389/fcimb.2020.00413 32903432 PMC7434969

[B293] Oliveira V. von Rosenberg W. Montaldo P. Adjei T. Mendoza J. Shivamurthappa V.. (2019). Early postnatal heart rate variability in healthy newborn infants. Front. Physiol. 10:922. 10.3389/fphys.2019.00922 31440164 PMC6692663

[B294] Ouellet-Morin I. Dionne G. Lupien S. J. Muckle G. Côté S. Pérusse D.. (2011). Prenatal alcohol exposure and cortisol activity in 19-month-old toddlers: an investigation of the moderating effects of sex and testosterone. Psychopharmacology (Berl.) 214, 297–307. 10.1007/s00213-010-1955-z 20717651

[B295] Oyebode F. Rastogi A. Berrisford G. Coccia F. (2012). Psychotropics in pregnancy: safety and other considerations. Pharmacol. Ther. 135, 71–77. 10.1016/j.pharmthera.2012.03.008 22483705

[B296] Ozawa M. Sasaki M. Kanda K. (2010). Effect of procedure light on the physiological responses of preterm infants: effects of procedure light on neonates. Jpn. J. Nurs. Sci. 7, 76–83. 10.1111/j.1742-7924.2010.00142.x 20618679

[B297] Parris K. M. Amabebe E. Cohen M. C. Anumba D. O. (2021). Placental microbial–metabolite profiles and inflammatory mechanisms associated with preterm birth. J. Clin. Pathol. 74, 10–18. 10.1136/jclinpath-2020-206536 32796048

[B298] Passi R. Doheny K. K. Gordin Y. Hinssen H. Palmer C. (2017). Electrical grounding improves vagal tone in preterm infants. Neonatology 112, 187–192. 10.1159/000475744 28601861 PMC5542808

[B299] Patural H. Pichot V. Flori S. Giraud A. Franco P. Pladys P.. (2019). Autonomic maturation from birth to 2 years: normative values. Heliyon 5:e01300. 10.1016/j.heliyon.2019.e01300 30899829 PMC6407160

[B300] Pavlov V. A. Tracey K. J. (2012). The vagus nerve and the inflammatory reflex—linking immunity and metabolism. Nat. Rev. Endocrinol. 8, 743–754. 10.1038/nrendo.2012.189 23169440 PMC4082307

[B301] Peaceman A. M. Meyer B. A. Thorp J. A. Parisi V. M. Creasy R. K. (1989). The effect of magnesium sulfate tocolysis on the fetal biophysical profile. Am. J. Obstet. Gynecol. 161, 771–774. 10.1016/0002-9378(89)90399-2 2782360

[B302] Peleg D. Goldman J. A. (1980). Fetal heart rate acceleration in response to light Stimulation äs a clinical measure of fetal well-being. A preliminary report. J. Perinat. Med. 8, 38–41 7365670 10.1515/jpme.1980.8.1.38

[B303] Pelzer E. Gomez-Arango L. F. Barrett H. L. Nitert M. D. (2017). Review: Maternal health and the placental microbiome. Placenta 54, 30–37. 10.1016/j.placenta.2016.12.003 28034467

[B304] Persson M. Winkvist A. Dahlgren L. Mogren I. (2013). Struggling with daily life and enduring pain”: a qualitative study of the experiences of pregnant women living with pelvic girdle pain. BMC Pregnancy Childbirth 13:111. 10.1186/1471-2393-13-111 23668823 PMC3661342

[B305] Peterson L. S. Stelzer I. A. Tsai A. S. Ghaemi M. S. Han X. Ando K.. (2020). Multiomic immune clockworks of pregnancy. Semin. Immunopathol. 42, 397–412. 10.1007/s00281-019-00772-1 32020337 PMC7508753

[B306] Pien G. W. Schwab R. J. (2004). Sleep disorders during pregnancy. Sleep 27, 1405–1417. 10.1093/sleep/27.7.1405 15586794

[B307] Podymow T. August P. (2008). Update on the use of antihypertensive drugs in pregnancy. Hypertension 51, 960–969. 10.1161/HYPERTENSIONAHA.106.075895 18259046

[B308] Podymow T. August P. (2011). Antihypertensive drugs in pregnancy. Semin. Nephrol. 31, 70–85. 10.1016/j.semnephrol.2010.10.007 21266266

[B309] Porges S. W. (2011). The Polyvagal Theory: Neurophysiological Foundations of Emotions, Attachment, Communication, and Self-Regulation, 1st Edn. New York, NY: W. W. Norton.

[B310] Porges S. W. Furman S. A. (2011). The early development of the autonomic nervous system provides a neural platform for social behavior: a polyvagal perspective. Infant Child Dev. 20, 106–118. 10.1002/icd.688 21516219 PMC3079208

[B311] Praud J.-P. Miura Y. Frasch M. G. (2017). Animal models for the study of neonatal disease, in Animal Models for the Study of Human Disease, ed Michael Conn P. (London: Academic Press) 805–837. 10.1016/B978-0-12-809468-6.00031-0

[B312] Prechtl H. F. (1990). Qualitative changes of spontaneous movements in fetus and preterm infant are a marker of neurological dysfunction. Early Hum. Dev. 23, 151–158. 10.1016/0378-3782(90)90011-7 2253578

[B313] Preston R. Crosby E. T. Kotarba D. Dudas H. Elliott R. D. (1993). Maternal positioning affects fetal heart rate changes after epidural analgesia for labour. Can. J. Anaesth. J. Can. Anesth. 40, 1136–1141. 10.1007/BF03009602 8281589

[B314] Prince A. L. Ma J. Kannan P. S. Alvarez M. Gisslen T. Harris R. A.. (2016). The placental membrane microbiome is altered among subjects with spontaneous preterm birth with and without chorioamnionitis. Am. J. Obstet. Gynecol. 214, 627.e1–627.e16. 10.1016/j.ajog.2016.01.193 26965447 PMC4909356

[B315] Prins J. R. Eskandar S. Eggen B. J. L. Scherjon S. A. (2018). Microglia, the missing link in maternal immune activation and fetal neurodevelopment; and a possible link in preeclampsia and disturbed neurodevelopment? J. Reprod. Immunol. 126, 18–22. 10.1016/j.jri.2018.01.004 29421625

[B316] Pryor J. R. Maalouf F. I. Krans E. E. Schumacher R. E. Cooper W. O. Patrick S. W. (2017). The opioid epidemic and neonatal abstinence syndrome in the USA: a review of the continuum of care. Arch. Dis. Child. Fetal Neonatal Ed. 102, F183–F187. 10.1136/archdischild-2015-310045 28073819 PMC5730450

[B317] Raczyńska A. Gulczyńska E. (2019). The impact of positioning on bottle-feeding in preterm infants (≤ 34 GA). A comparative study of the semi-elevated and the side-lying position - a pilot study. Dev. Period Med. 23, 117–124. 31280248 10.34763/devperiodmed.20192302.117124 PMC8522368

[B318] Ramsey P. S. Rouse D. J. (2001). Magnesium sulfate as a tocolytic agent. Semin. Perinatol. 25, 236–247. 10.1053/sper.2001.27546 11561911

[B319] Rantakari K. Rinta-Koski O.-P. Metsäranta M. Hollmén J. Särkkä S. Rahkonen P.. (2021). Early oxygen levels contribute to brain injury in extremely preterm infants. Pediatr. Res. 90, 131–139. 10.1038/s41390-021-01460-3 33753894 PMC7984503

[B320] Ray-Griffith S. L. Wendel M. P. Stowe Z. N. Magann E. F. (2018). Chronic pain during pregnancy: a review of the literature. Int. J. Womens Health 10, 153–164. 10.2147/IJWH.S151845 29692634 PMC5901203

[B321] Reich D. A. Govindan R. B. Whitehead M. T. Wang J. Chang T. Kota S.. (2019). The effect of unilateral stroke on autonomic function in the term newborn. Pediatr. Res. 85, 830–834. 10.1038/s41390-019-0320-0 30712058

[B322] Reid N. Moritz K. M. Akison L. K. (2019). Adverse health outcomes associated with fetal alcohol exposure: a systematic review focused on immune-related outcomes. Pediatr. Allergy Immunol. 30, 698–707. 10.1111/pai.13099 31215695

[B323] Rezk A. Y. Abdulqawi K. Mustafa R. M. El-Azm T. M. A. Al-Inany H. (2008). Fetal and neonatal responses following maternal exposure to mobile phones. Saudi Med. J. 29, 218–223. 18246230

[B324] Rivolta M. W. Stampalija T. Casati D. Richardson B. S. Ross M. G. Frasch M. G.. (2014). Acceleration and deceleration capacity of fetal heart rate in an *in-vivo* sheep model. PLoS ONE 9:e104193. 10.1371/journal.pone.0104193 25141131 PMC4139279

[B325] Rodgers S. K. Chang C. DeBardeleben J. T. Horrow M. M. (2015). Normal and abnormal US findings in early first-trimester pregnancy: review of the society of radiologists in ultrasound 2012 consensus panel recommendations. RadioGraphics 35, 2135–2148. 10.1148/rg.2015150092 26562242

[B326] Roşu G. Samoilescu G. Rau M. C. Baltag O. (2016). Aspects regarding the occupational and non-occupational exposure to low frequency and radiofrequency electromagnetic fields, in 2016 International Conference on Applied and Theoretical Electricity (ICATE) (Craiova), 1–6. 10.1109/ICATE.2016.7754700

[B327] Roux S. G. Garnier N. B. Abry P. Gold N. Frasch M. G. (2021). Distance to healthy cardiovascular dynamics from fetal heart rate scale-dependent features in pregnant sheep model of human labor predicts cardiovascular decompensation. ArXiv210207768 Phys. Q-Bio. Available online at: http://arxiv.org/abs/2102.07768 (accessed May 13, 2021)> 10.3389/fped.2021.660476 PMC8369259 34414140

[B328] Russell N. E. Higgins M. F. Kinsley B. F. Foley M. E. McAuliffe F. M. (2016). Heart rate variability in neonates of type 1 diabetic pregnancy. Early Hum. Dev. 92, 51–55. 10.1016/j.earlhumdev.2015.11.003 26658058

[B329] Sabir H. Stannigel H. Schwarz A. Hoehn T. (2010). Perinatal hemorrhagic shock after fetal scalp blood sampling. Obstet. Gynecol. 115, 419–420. 10.1097/AOG.0b013e3181c51aeb 20093865

[B330] Sadler T. W. (2012). Langman Medical Embryology. Philadelphia, PA: Llippincott Williams and Wilkins.

[B331] Sahin F. K. Koken G. Cosar E. Saylan F. Fidan F. Yilmazer M.. (2008). Obstructive sleep apnea in pregnancy and fetal outcome. Int. J. Gynecol. Obstet. 100, 141–146. 10.1016/j.ijgo.2007.08.012 17976624

[B332] Sahota P. K. Jain S. S. Dhand R. (2003). Sleep disorders in pregnancy. Curr. Opin. Pulm. Med. 9, 477–483. 10.1097/00063198-200311000-00005 14534398

[B333] Sanghavi M. Rutherford J. D. (2014). Cardiovascular physiology of pregnancy. Circulation 130, 1003–1008. 10.1161/CIRCULATIONAHA.114.009029 25223771

[B334] Santiago J. R. Nolledo M. S. Kinzler W. Santiago T. V. (2001). Sleep and sleep disorders in pregnancy. Ann. Intern. Med. 134, 396–408. 10.7326/0003-4819-134-5-200103060-00012 11242500

[B335] Sarkar P. Lobmaier S. Fabre B. González D. Mueller A. Frasch M. G.. (2021). Detection of maternal and fetal stress from the electrocardiogram with self-supervised representation learning. ArXiv201102000 Eess Q-Bio. Available online at: http://arxiv.org/abs/2011.02000 (accessed May 13, 2021). 10.1038/s41598-021-03376-8 PMC8683397 34921162

[B336] Satoh T. Sakurai E. Tada H. Masuda T. (2009). Ontogeny of reticular framework of white pulp and marginal zone in human spleen: immunohistochemical studies of fetal spleens from the 17th to 40th week of gestation. Cell Tissue Res. 336, 287–297. 10.1007/s00441-009-0757-2 19255788

[B337] Schifrin B. S. (2020). Electronic fetal monitoring-prevention or rescue? Front. Pediatr. 8:503. 10.3389/fped.2020.00503 32984215 PMC7481352

[B338] Schlatterer S. D. Govindan R. B. Barnett S. D. Al-Shargabi T. Reich D. A. Iyer S.. (2021). Autonomic development in preterm infants is associated with morbidity of prematurity. Pediatr. Res. 1–7. 10.1038/s41390-021-01420-x. [Epub ahead of print]. 33654284 PMC7922721

[B339] Schmidt A. Schneider U. Witte O. W. Schleußner E. Hoyer D. (2014). Developing fetal motor-cardiovascular coordination analyzed from multi-channel magnetocardiography. Physiol. Meas. 35, 1943–1959. 10.1088/0967-3334/35/10/1943 25229562

[B340] Schmidt W. Cseh I. Hara K. Kubli F. (1984). Maternal perception, tocodynamometric findings and real-time ultrasound assessment of total fetal activity. Int. J. Gynaecol. Obstet. Off. Organ Int. Fed. Gynaecol. Obstet. 22, 85–90. 10.1016/0020-7292(84)90018-3 6145643

[B341] Schneebaum Sender N. Govindan R. B. Whitehead M. T. Massaro A. N. Metzler M. Wang J.. (2017). Cerebral modulation of the autonomic nervous system in term infants. J. Perinatol. 37, 558–562. 10.1038/jp.2016.248 28079874

[B342] Schneider U. Bode F. Schmidt A. Nowack S. Rudolph A. Doelcker E.-M.. (2018). Developmental milestones of the autonomic nervous system revealed via longitudinal monitoring of fetal heart rate variability. PLOS ONE 13:e0200799. 10.1371/journal.pone.0200799 30016343 PMC6049949

[B343] Schneider U. Fiedler A. Schröder B. Jaekel S. Stacke A. Hoyer D.. (2010). The effect of antenatal steroid treatment on fetal autonomic heart rate regulation revealed by fetal magnetocardiography (fMCG). Early Hum. Dev. 86, 319–325. 10.1016/j.earlhumdev.2010.05.018 20547444

[B344] Schneider U. Frank B. Fiedler A. Kaehler C. Hoyer D. Liehr M.. (2008). Human fetal heart rate variability-characteristics of autonomic regulation in the third trimester of gestation. J. Perinat. Med. 36. 10.1515/JPM.2008.059 18605969

[B345] Schulz L. C. (2010). The Dutch Hunger Winter and the developmental origins of health and disease. Proc. Natl. Acad. Sci. U.S.A. 107, 16757–16758. 10.1073/pnas.1012911107 20855592 PMC2947916

[B346] Seckl J. R. Meaney M. J. (2004). Glucocorticoid programming. Ann. N. Y. Acad. Sci. 1032, 63–84. 10.1196/annals.1314.006 15677396

[B347] Sehmbi H. D'Souza R. Bhatia A. (2017). Low back pain in pregnancy: investigations, management, and role of neuraxial analgesia and anaesthesia: a systematic review. Gynecol. Obstet. Invest. 82, 417–436. 10.1159/000471764 28514779

[B348] Selander J. Rylander L. Albin M. Rosenhall U. Lewné M. Gustavsson P. (2019). Full-time exposure to occupational noise during pregnancy was associated with reduced birth weight in a nationwide cohort study of Swedish women. Sci. Total Environ. 651, 1137–1143. 10.1016/j.scitotenv.2018.09.212 30360245

[B349] Selye H. (1950). The Physiology and Pathology of Exposure to Stress, 1st Edn. Montreal, QC: Acta Medica Publ.

[B350] Senat M. V. Minoui S. Multon O. Fernandez H. Frydman R. Ville Y. (1998). Effect of dexamethasone and betamethasone on fetal heart rate variability in preterm labour: a randomised study. BJOG Int. J. Obstet. Gynaecol. 105, 749–755. 10.1111/j.1471-0528.1998.tb10206.x 9692416

[B351] Senturk M. B. Cakmak Y. Gündogdu M. Polat M. Atac H. (2015). Does performing cesarean section after onset of labor has positive effect on neonatal respiratory disorders? J. Matern. Fetal Neonatal Med. 29, 2457–2460. 10.3109/14767058.2015.1087499 26381371

[B352] Sharma R. Hudak M. L. (2013). A clinical perspective of necrotizing enterocolitis: past, present, and future. Clin. Perinatol. 40, 27–51. 10.1016/j.clp.2012.12.012 23415262 PMC3575605

[B353] Shea C. A. Rolfe R. A. Murphy P. (2015). The importance of foetal movement for co-ordinated cartilage and bone development *in utero*. Bone Jt. Res. 4, 105–116. 10.1302/2046-3758.47.2000387 26142413 PMC4602203

[B354] Shekhawat H. Shrivastava D. Dhurve K. Shelke U. Deo A. (2020). Effect of epidural labor analgesia on fetal heart rate and neonatal outcome. J. Datta Meghe Inst. Med. Sci. Univ. India 15, 382–386. 10.4103/jdmimsu.jdmimsu_194_20

[B355] Sheng J. A. Bales N. J. Myers S. A. Bautista A. I. Roueinfar M. Hale T. M.. (2020). The hypothalamic-pituitary-adrenal axis: development, programming actions of hormones, and maternal-fetal interactions. Front. Behav. Neurosci. 14:601939. 10.3389/fnbeh.2020.601939 33519393 PMC7838595

[B356] Sherer M. L. Posillico C. K. Schwarz J. M. (2017). An examination of changes in maternal neuroimmune function during pregnancy and the postpartum period. Brain. Behav. Immun. 66, 201–209. 10.1016/j.bbi.2017.06.016 28669797 PMC6348474

[B357] Sherer M. L. Posillico C. K. Schwarz J. M. (2018). The psychoneuroimmunology of pregnancy. Front. Neuroendocrinol. 51, 25–35. 10.1016/j.yfrne.2017.10.006 29110974

[B358] Shiroiwa Y. Kamiya Y. Uchibori S. Inukai K. Kito H. Shibata T.. (1986). Activity, cardiac and respiratory responses of blindfold preterm infants in a neonatal intensive care unit. Early Hum. Dev. 14, 259–265. 10.1016/0378-3782(86)90187-8 3803271

[B359] Shuffrey L. C. Myers M. M. Odendaal H. J. Elliott A. J. du Plessis C. Groenewald C.. (2019). Fetal heart rate, heart rate variability, and heart rate/movement coupling in the safe passage study. J. Perinatol. 39, 608–618. 10.1038/s41372-019-0342-9 30833637 PMC6483837

[B360] Siddiqui S. Fifer W. P. Ordonez-Retamar M. Nugent J. D. Williams I. A. (2017). An antenatal marker of neurodevelopmental outcomes in infants with congenital heart disease. J. Perinatol. 37, 953–957. 10.1038/jp.2017.59 28471442 PMC5578909

[B361] Silasi M. Cardenas I. Kwon J.-Y. Racicot K. Aldo P. Mor G. (2015). Viral infections during pregnancy. Am. J. Reprod. Immunol. 73, 199–213. 10.1111/aji.12355 25582523 PMC4610031

[B362] Simkin J. E. Zhang D. Stamp L. A. Newgreen D. F. (2019). Fine scale differences within the vagal neural crest for enteric nervous system formation. Dev. Biol. 446, 22–33. 10.1016/j.ydbio.2018.11.007 30448439

[B363] Sival D. A. Visser G. H. Prechtl H. F. (1990). Does reduction of amniotic fluid affect fetal movements? Early Hum. Dev. 23, 233–246. 10.1016/0378-3782(90)90014-a 2253581

[B364] Sivojelezova A. Shuhaiber S. Sarkissian L. Einarson A. Koren G. (2005). Citalopram use in pregnancy: prospective comparative evaluation of pregnancy and fetal outcome. Am. J. Obstet. Gynecol. 193, 2004–2009. 10.1016/j.ajog.2005.05.012 16325604

[B365] Sklerov M. Dayan E. Browner N. (2019). Functional neuroimaging of the central autonomic network: recent developments and clinical implications. Clin. Auton. Res. 29, 555–566. 10.1007/s10286-018-0577-0 30470943 PMC6858471

[B366] Smith M. Galbraith W. Blumer J. (2018). Reducing low back and posterior pelvic pain during and after pregnancy using OMT. J. Am. Osteopath. Assoc. 118, 487–488. 10.7556/jaoa.2018.108 29946672

[B367] Snijder C. A. Roeleveld N. te Velde E. Steegers E. A. P. Raat H. Hofman A.. (2012). Occupational exposure to chemicals and fetal growth: the generation R study. Hum. Reprod. 27, 910–920. 10.1093/humrep/der437 22215632 PMC3279127

[B368] Soma-Pillay P. Nelson-Piercy C. Tolppanen H. Mebazaa A. (2016). Physiological changes in pregnancy. Cardiovasc. J. Afr. 27, 89–94. 10.5830/CVJA-2016-021 27213856 PMC4928162

[B369] Sørensen N. Murata K. Budtz-Jørgensen E. Weihe P. Grandjean P. (1999). Prenatal methylmercury exposure as a cardiovascular risk factor at seven years of age. Epidemiology 10, 370–375. 10401870

[B370] Sorokin Y. Dierker L. J. Pillay S. K. Zador I. E. Schreiner M. L. Rosen M. G. (1982). The association between fetal heart rate patterns and fetal movements in pregnancies between 20 and 30 weeks' gestation. Am. J. Obstet. Gynecol. 143, 243–249. 10.1016/0002-9378(82)90812-2 7081342

[B371] Spinillo A. Viazzo F. Colleoni R. Chiara A. Maria Cerbo R. Fazzi E. (2004). Two-year infant neurodevelopmental outcome after single or multiple antenatal courses of corticosteroids to prevent complications of prematurity. Am. J. Obstet. Gynecol. 191, 217–224. 10.1016/j.ajog.2003.12.023 15295369

[B372] Stallworth J. C. Yeh S.-Y. Petrie R. H. (1981). The effect of magnesium sulfate on fetal heart rate variability and uterine activity. Am. J. Obstet. Gynecol. 140, 702–706. 10.1016/0002-9378(81)90208-8 7258244

[B373] Starling P. Charlton K. McMahon A. T. Lucas C. (2015). Fish intake during pregnancy and foetal neurodevelopment–a systematic review of the evidence. Nutrients 7, 2001–2014. 10.3390/nu7032001 25793632 PMC4377896

[B374] Stéphan-Blanchard E. Chardon K. Léké A. Delanaud S. Bach V. Telliez F. (2013). Heart rate variability in sleeping preterm neonates exposed to cool and warm thermal conditions. PLoS ONE 8:e68211. 10.1371/journal.pone.0068211 23840888 PMC3698119

[B375] Stone M. L. Tatum P. M. Weitkamp J.-H. Mukherjee A. B. Attridge J. McGahren E. D.. (2013). Abnormal heart rate characteristics before clinical diagnosis of necrotizing enterocolitis. J. Perinatol. Off. J. Calif. Perinat. Assoc. 33, 847–850. 10.1038/jp.2013.63 23722974 PMC4026091

[B376] Subtil D. Tiberghien P. Devos P. Therby D. Leclerc G. Vaast P.. (2003). Immediate and delayed effects of antenatal corticosteroids on fetal heart rate: a randomized trial that compares betamethasone acetate and phosphate, betamethasone phosphate, and dexamethasone. Am. J. Obstet. Gynecol. 188, 524–531. 10.1067/mob.2003.136 12592266

[B377] Sun S. Spangler K. R. Weinberger K. R. Yanosky J. D. Braun J. M. Wellenius G. A. (2019). Ambient temperature and markers of fetal growth: a retrospective observational study of 29 million U.S. singleton births. Environ. Health Perspect. 127:067005. 10.1289/EHP4648 31162981 PMC6792370

[B378] Suri R. Altshuler L. Hellemann G. Burt V. K. Aquino A. Mintz J. (2007). Effects of antenatal depression and antidepressant treatment on gestational age at birth and risk of preterm birth. Am. J. Psychiatry 164, 1206–1213. 10.1176/appi.ajp.2007.06071172 17671283

[B379] Tan C. M. J. Lewandowski A. J. (2020). The transitional heart: from early embryonic and fetal development to neonatal life. Fetal Diagn. Ther. 47, 373–386. 10.1159/000501906 31533099 PMC7265763

[B380] Task Force of the European Society of Cardiology and the North American Society of Pacing and Electrophysiology (1996). Heart rate variability. Standards of measurement, physiological interpretation, and clinical use. Eur. Heart J. 17, 354–381. 8737210

[B381] Tendais I. Figueiredo B. Gonçalves H. Bernardes J. Ayres-de-Campos D. Montenegro N. (2015). Sex differences in the fetal heart rate variability indices of twins. J. Perinat. Med. 43, 221–225. 10.1515/jpm-2014-0031 24945419

[B382] Thanaboonyawat I. Wataganara T. Boriboonhiransarn D. Viboonchart S. Tontisirin P. (2006). Effect of halogen light in fetal stimulation for fetal well-being assessment. J. Med. Assoc. Thail. 89, 1376–1380. 17100372

[B383] Thayer J. F. Brosschot J. F. (2005). Psychosomatics and psychopathology: looking up and down from the brain. Psychoneuroendocrinology 30, 1050–1058. 10.1016/j.psyneuen.2005.04.014 16005156

[B384] Thome J. Densmore M. Frewen P. A. McKinnon M. C. Théberge J. Nicholson A. A.. (2017). Desynchronization of autonomic response and central autonomic network connectivity in posttraumatic stress disorder. Hum. Brain Mapp. 38, 27–40. 10.1002/hbm.23340 27647521 PMC6866719

[B385] Thoyre S. M. Holditch-Davis D. Schwartz T. A. Melendez Roman C. R. Nix W. (2012). Coregulated approach to feeding preterm infants with lung disease: effects during feeding. Nurs. Res. 61, 242–251. 10.1097/NNR.0b013e31824b02ad 22565102 PMC5758360

[B386] Tong L. Kalish B. T. (2020). The impact of maternal obesity on childhood neurodevelopment. J. Perinatol. 41, 928–939. 10.1038/s41372-020-00871-0 33249428

[B387] Tracey K. J. (2007). Physiology and immunology of the cholinergic antiinflammatory pathway. J. Clin. Invest. 117, 289–296. 10.1172/JCI30555 17273548 PMC1783813

[B388] Tracey K. J. (2009). Reflex control of immunity. Nat. Rev. Immunol. 9, 418–428. 10.1038/nri2566 19461672 PMC4535331

[B389] Tribe R. M. Taylor P. D. Kelly N. M. Rees D. Sandall J. Kennedy H. P. (2018). Parturition and the perinatal period: can mode of delivery impact on the future health of the neonate? J. Physiol. 596, 5709–5722. 10.1113/JP275429 29533463 PMC6265543

[B390] Tribollet E. Charpak S. Schmidt A. Dubois-Dauphin M. Dreifuss J. J. (1989). Appearance and transient expression of oxytocin receptors in fetal, infant, and peripubertal rat brain studied by autoradiography and electrophysiology. J. Neurosci. 9, 1764–1773. 10.1523/JNEUROSCI.09-05-01764.1989 2542479 PMC6569831

[B391] Tsuda K. Iwata S. Mukai T. Shibasaki J. Takeuchi A. Ioroi T.. (2018). Body temperature, heart rate, and short-term outcome of cooled infants. Ther. Hypothermia Temp. Manag. 9, 76–85. 10.1089/ther.2018.0019 30230963 PMC6434598

[B392] Uchitel J. Vanhatalo S. Austin T. (2021). Early development of sleep and brain functional connectivity in term-born and preterm infants. Pediatr. Res. 10.1038/s41390-021-01497-4. [Epub ahead of print]. 33859364

[B393] Ulmer Yaniv A. Salomon R. Waidergoren S. Shimon-Raz O. Djalovski A. Feldman R. (2021). Synchronous caregiving from birth to adulthood tunes humans' social brain. Proc. Natl. Acad. Sci. U.S.A. 118:e2012900118. 10.1073/pnas.2012900118 33785591 PMC8040627

[B394] Urfer-Maurer N. Ludyga S. Stalder T. Brand S. Holsboer-Trachsler E. Gerber M.. (2018). Heart rate variability and salivary cortisol in very preterm children during school age. Psychoneuroendocrinology 87, 27–34. 10.1016/j.psyneuen.2017.10.004 29035709

[B395] Uvnäs-Moberg K. Ekström-Bergström A. Berg M. Buckley S. Pajalic Z. Hadjigeorgiou E.. (2019). Maternal plasma levels of oxytocin during physiological childbirth – a systematic review with implications for uterine contractions and central actions of oxytocin. BMC Pregnancy Childbirth 19:285. 10.1186/s12884-019-2365-9 31399062 PMC6688382

[B396] Van den Bergh B. R. H. Mulder E. J. H. (2012). Fetal sleep organization: a biological precursor of self-regulation in childhood and adolescence? Biol. Psychol. 89, 584–590. 10.1016/j.biopsycho.2012.01.003 22266165

[B397] van Geijn H. P. Jongsma H. W. Doesburg W. H. Lemmens W. A. J. G. de Haan J. Eskes T. K. A. B. (1980). The effect of diazepam administration during pregnancy or labor on the heart rate variability of the newborn infant. Eur. J. Obstet. Gynecol. Reprod. Biol. 10, 187–201. 10.1016/0028-2243(80)90060-X 7189484

[B398] Venkata C. Venkateshiah S. B. (2009). Sleep-disordered breathing during pregnancy. J. Am. Board Fam. Med. 22, 158–168. 10.3122/jabfm.2009.02.080057 19264939

[B399] Venkataraman R. Kamaluddeen M. Amin H. Lodha A. (2018). Is less noise, light and parental/caregiver stress in the neonatal intensive care unit better for neonates? Indian Pediatr. 55, 17–21. 10.1007/s13312-018-1220-9 29396929

[B400] Verbruggen S. W. Kainz B. Shelmerdine S. C. Hajnal J. V. Rutherford M. A. Arthurs O. J.. (2018). Stresses and strains on the human fetal skeleton during development. J. R. Soc. Interface 15:20170593. 10.1098/rsif.2017.0593 29367236 PMC5805961

[B401] Verdurmen K. M. J. Hulsenboom A. D. J. Laar J. O. E. H. van Oei S. G. (2017). Effect of tocolytic drugs on fetal heart rate variability: a systematic review. J. Matern. Fetal Neonatal Med. 30, 2387–2394. 10.1080/14767058.2016.1249844 27756155

[B402] Verdurmen K. M. J. Renckens J. van Laar J. O. E. H. Oei S. G. (2013). The influence of corticosteroids on fetal heart rate variability: a systematic review of the literature. Obstet. Gynecol. Surv. 68, 811–824. 10.1097/OGX.0000000000000007 25102018

[B403] Verdurmen K. M. J. Warmerdam G. J. J. Lempersz C. Hulsenboom A. D. J. Renckens J. Dieleman J. P.. (2018). The influence of betamethasone on fetal heart rate variability, obtained by non-invasive fetal electrocardiogram recordings. Early Hum. Dev. 119, 8–14. 10.1016/j.earlhumdev.2018.02.011 29505915

[B404] Vermani E. Mittal R. Weeks A. (2010). Pelvic girdle pain and low back pain in pregnancy: a review. Pain Pract. Off. J. World Inst. Pain 10, 60–71. 10.1111/j.1533-2500.2009.00327.x 19863747

[B405] Verriotis M. Chang P. Fitzgerald M. Fabrizi L. (2016). The development of the nociceptive brain. Neuroscience 338, 207–219. 10.1016/j.neuroscience.2016.07.026 27457037

[B406] Verspyck E. Sentilhes L. (2008). [Abnormal fetal heart rate patterns associated with different labour managements and intrauterine resuscitation techniques]. J. Gynecol. Obstet. Biol. Reprod. (Paris). 37(Suppl 1), S56–S64. 10.1016/j.jgyn.2007.11.011 18187267

[B407] Viscomi C. M. Hood D. D. Melone P. J. Eisenach J. C. (1990). Fetal heart rate variability after epidural fentanyl during labor. Anesth. Analg. 71, 679–683. 10.1213/00000539-199012000-00018 2240642

[B408] Visser G. H. A. Mulder E. J. H. Prechtl H. F. R. (1992). Studies on developmental neurology in the human fetus. Dev. Pharmacol. Ther. 18, 175–183. 10.1159/000480620 1306806

[B409] Vitetta L. Vitetta G. Hall S. (2018). The brain–intestinal mucosa–appendix– microbiome–brain loop. Diseases 6:23. 10.3390/diseases6020023 29614774 PMC6023457

[B410] Vohr B. R. Davis E. P. Wanke C. A. Krebs N. F. (2017). Neurodevelopment: the impact of nutrition and inflammation during preconception and pregnancy in low-resource settings. Pediatrics 139, S38–S49. 10.1542/peds.2016-2828F 28562247

[B411] Walker R. W. Clemente J. C. Peter I. Loos R. J. F. (2017). The prenatal gut microbiome: are we colonized with bacteria *in utero*? Pediatr. Obes. 12, 3–17. 10.1111/ijpo.12217 28447406 PMC5583026

[B412] Wallingford M. C. C B. Nw C. Mt C. Mg F. (2018). Placental vascular calcification and cardiovascular health: it is time to determine how much of maternal and offspring health is written in stone. Front. Physiol. 9:1044. 10.3389/fphys.2018.01044 30131710 PMC6090024

[B413] Walusinski O. (2006). Yawning: unsuspected avenue for a better understanding of arousal and interoception. Med. Hypotheses 67, 6–14. 10.1016/j.mehy.2006.01.020 16520004

[B414] Walusinski O. (2010). Fetal yawning, in Frontiers of Neurology and Neuroscience, ed. Walusinski O. (Basel: KARGER), 32–41. 10.1159/000307075 20357460

[B415] Walusinski O. (2014). How yawning switches the default-mode network to the attentional network by activating the cerebrospinal fluid flow: how yawning switches the DMN to the attentional network. Clin. Anat. 27, 201–209. 10.1002/ca.22280 23813685

[B416] Wang M. Wang Z.-P. Gao L.-J. Yang H. Zhao Z.-T. (2015). Maternal consumption of non-staple food in the first trimester and risk of neural tube defects in offspring. Nutrients 7, 3067–3077. 10.3390/nu7053067 25919306 PMC4446739

[B417] Wang X. Durosier L. D. Ross M. G. Richardson B. S. Frasch M. G. (2014). Online detection of fetal acidemia during labour by testing synchronization of EEG and heart rate: a prospective study in fetal sheep. PLoS ONE 9:e108119. 10.1371/journal.pone.0108119 25268842 PMC4182309

[B418] Warland J. Dorrian J. Morrison J. L. O'Brien L. M. (2018). Maternal sleep during pregnancy and poor fetal outcomes: a scoping review of the literature with meta-analysis. Sleep Med. Rev. 41, 197–219. 10.1016/j.smrv.2018.03.004 29910107

[B419] Weber A. Harrison T. M. (2019). Reducing toxic stress in the neonatal intensive care unit to improve infant outcomes. Nurs. Outlook 67, 169–189. 10.1016/j.outlook.2018.11.002 30611546 PMC6450772

[B420] Westerhuis M. E. M. H. Kwee A. van Ginkel A. A. Drogtrop A. P. Gyselaers W. J. A. Visser G. H. A. (2007). Limitations of ST analysis in clinical practice: three cases of intrapartum metabolic acidosis. BJOG Int. J. Obstet. Gynaecol. 114, 1194–1201. 10.1111/j.1471-0528.2007.01236.x 17501963

[B421] Wilder R. L. (1998). Hormones, pregnancy, and autoimmune diseases. Ann. N. Y. Acad. Sci. 840, 45–50. 10.1111/j.1749-6632.1998.tb09547.x 9629235

[B422] Wilson J. D. Adams A. J. Murphy P. Eswaran H. Preissl H. (2008). Design of a light stimulator for fetal and neonatal magnetoencephalography. Physiol. Meas. 30, N1–N10. 10.1088/0967-3334/30/1/N01 19104135 PMC2965530

[B423] Woodson R. H. Jones N. G. da Costa Woodson E. Pollock S. Evans M. (1979). Fetal mediators of the relationships between increased pregnancy and labour blood pressure and newborn irritability. Early Hum. Dev. 3, 127–139. 10.1016/0378-3782(79)90002-1 535544

[B424] Yee J. R. Kenkel W. M. Frijling J. L. Dodhia S. Onishi K. G. Tovar S.. (2016). Oxytocin promotes functional coupling between paraventricular nucleus and both sympathetic and parasympathetic cardioregulatory nuclei. Horm. Behav. 80, 82–91. 10.1016/j.yhbeh.2016.01.010 26836772 PMC5768414

[B425] Yiallourou S. R. Sands S. A. Walker A. M. Horne R. S. C. (2012). Maturation of heart rate and blood pressure variability during sleep in term-born infants. Sleep 35, 177–186. 10.5665/sleep.1616 22294807 PMC3250356

[B426] Yurdakök K. (2012). View of environmental pollution and the fetus. J. Pediatr. Neonatal Individ. Med. 1, 33–42. 10.7363/010116

[B427] Zafarghandi N. Hadavand S. Davati A. Mohseni S. M. Kimiaiimoghadam F. Torkestani F. (2012). The effects of sleep quality and duration in late pregnancy on labor and fetal outcome. J. Matern. Fetal Neonatal Med. 25, 535–537. 10.3109/14767058.2011.600370 21827377

[B428] Zahr L. K. de Traversay J. (1995). Premature infant responses to noise reduction by earmuffs: effects on behavioral and physiologic measures. J. Perinatol. Off. J. Calif. Perinat. Assoc. 15, 448–455. 8648453

[B429] Zeskind P. S. Gingras J. L. (2006). Maternal cigarette-smoking during pregnancy disrupts rhythms in fetal heart rate. J. Pediatr. Psychol. 31, 5–14. 10.1093/jpepsy/jsj031 15905420

[B430] Zeskind P. S. Stephens L. E. (2004). Maternal selective serotonin reuptake inhibitor use during pregnancy and newborn neurobehavior. Pediatrics 113, 368–375. 10.1542/peds.113.2.368 14754951

[B431] Zhang J. Cai W.-W. Lee D. J. (1992). Occupational hazards and pregnancy outcomes. Am. J. Ind. Med. 21, 397–408. 10.1002/ajim.4700210312 1585950

[B432] Zhang X. Sliwowska J. H. Weinberg J. (2005). Prenatal alcohol exposure and fetal programming: effects on neuroendocrine and immune function. Exp. Biol. Med. 230, 376–388. 10.1177/15353702-0323006-05 15956767

[B433] Zheng J. Xiao X. Zhang Q. Mao L. Yu M. Xu J. (2015). The placental microbiome varies in association with low birth weight in full-term neonates. Nutrients 7, 6924–6937. 10.3390/nu7085315 26287241 PMC4555154

[B434] Zhou L. Xiao X. (2018). The role of gut microbiota in the effects of maternal obesity during pregnancy on offspring metabolism. Biosci. Rep. 38:BSR20171234. 10.1042/BSR20171234 29208770 PMC5897743

[B435] Zizzo A. R. Kirkegaard I. Hansen J. Uldbjerg N. Mølgaard H. (2020). Fetal heart rate variability is affected by fetal movements: a systematic review. Front. Physiol. 11:578898. 10.3389/fphys.2020.578898 33101059 PMC7554531

